# Supplementary material for: Discovery of 4-Anilinoquinolinylchalcone Derivatives as Potential NRF2 Activators
Source: Molecules. 2020 Jul 8;25(14):3133. doi: 10.3390/molecules25143133 (PMC7396997; doi:10.3390/molecules25143133)
Supplement: Supplementary file 1 [file molecules-25-03133-s001.pdf]

Supporting Information

for

Discovery of 4-anilinoquinolinylchalaone derivatives as potential NRF2 activators

Yu-Tse Kao[1], Yi-Siao Chen[2,3], Kai-Wei Tang[4], Jin-Ching Lee[3,5,6] Chih-Hua Tseng[4], Cherng-Chyi Tzeng[1], Chia-Hung Yen[3*], and Yeh-Long Chen [1,6,7*]

[1]Department of Medicinal and Applied Chemistry, College of Life Science, Kaohsiung Medical University, Kaohsiung City 807, Taiwan

[2]Ph.D. Program in Environmental and Occupational Medicine, College of Medicine, Kaohsiung Medical University, Kaohsiung City 807, Taiwan

[3]Graduate Institute of Natural Products, College of Pharmacy, Kaohsiung Medical University, Kaohsiung City 807, Taiwan

[4]School of Pharmacy, College of Pharmacy, Kaohsiung Medical University, Kaohsiung City 807, Taiwan

[5]Department of Biotechnology, College of Life Science, Kaohsiung Medical University, Kaohsiung City 807, Taiwan

[6]Drug Development and Value Creation Research Center, Kaohsiung Medical University, Kaohsiung 807, Taiwan

[7]Department of Medical Research, Kaohsiung Medical University Hospital, Kaohsiung Medical University, Kaohsiung 807, Taiwan

* Corresponding author. Tel.: +886 7 3121101 ext 2684; fax: +886 7 3125339.

E-mail addresses: yeloch@kmu.edu.tw; chyen@kmu.edu.tw

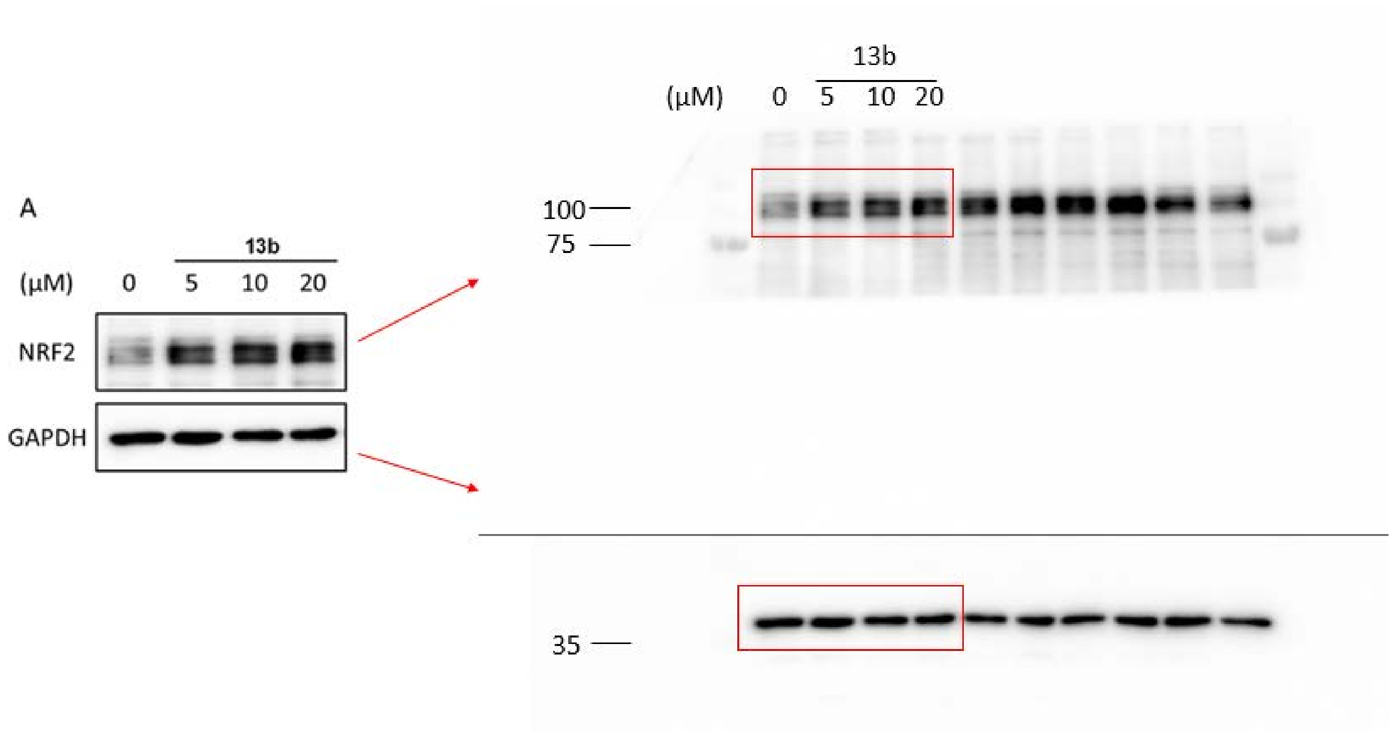

Figure S1. Densitometric analysis of Western Blot (compare Figure 5A).

Compound purity is determined by high performance liquid chromatography (HPLC).

HPLC methods used the following: HITACHI Chromaster 5110 (Hitachi High-Technologies, Tokyo, Japan) with UV detection at 254 nm and auto sampler; column, Mightysil RP-18GP (250×4.6 mm, 5 μm, Kento chemical, Tokyo, japan); mobile phase, method A: MeOH / 0.01M KH_2PO_4 (50:50), pH = 2.64; method B. MeOH / 0.01M KH_2PO_4 (50:50), pH = 2.94; method C. MeOH / 0.01M KH_2PO_4 (50:50), pH = 3.47; method D. MeOH / 0.01M KH_2PO_4 (60:40), pH = 2.88; method E. MeOH / 0.01M KH_2PO_4 (60:40), pH = 2.94; method F. MeOH / 0.01M KH_2PO_4 (60:40), pH = 3.47; and method G. MeOH / 0.01M KH_2PO_4 (80:20), pH = 3.47; and the flow rate was 1 mL/min; sample injection, 100 μM (1.0 mg dissolved in 0.5 mL DMSO, diluted with MeOH to 1 mL, then 0.5 mL compound was diluted with MeOH to 1 mL).

Chromaster System Manager Report

Analyzed Date and Time: 2020/03/17 01:58 下午 Reported Date and Time: 2020/03/17 02:15:14 下午

Processed Date and Time: 2020/03/17 02:13 下午

Data Path: C:\WIN32APP\CHROMASTER\TKW\DATA\0123\

Processing Method: KYT

System (acquisition): Sys 1 Series: 0123

Application(data): TKW Vial Number: 1

Sample Name: 5427 Vial Type: UNK

Injection from this vial: 1 of 1 Volume: 20.0 ul

Sample Description:

Chrom Type: Chromaster Channel : 1

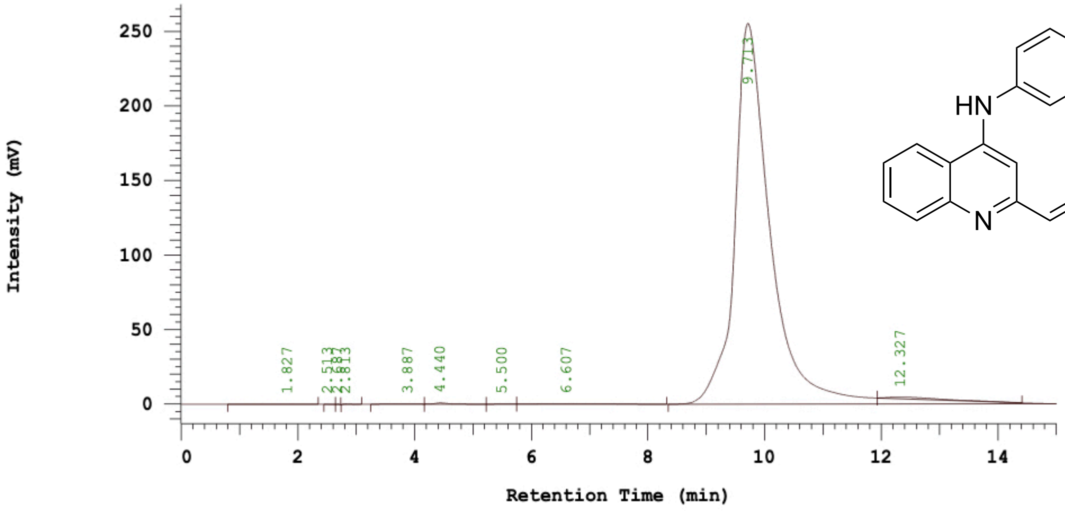

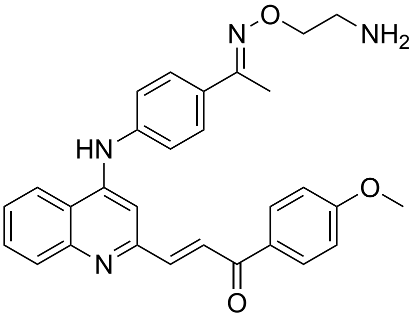

Processing Method: KYT
Method Developer: Hitachi
Pump 1: 5110
Pump 1 Solvent A: Pump 1 Solvent B:
Pump 1 Solvent C: Pump 1 Solvent D:
Method Description: MeOH / 10mM KH2PO4 = 50 / 50 (pH = 2.94)

Chrom Type: Chromaster Channel : 1
Peak Quantitation: AREA
Calculation Method: AREA%

No.	RT	Area	Conc 1	BC
1	1.827	2380	0.021	BB
2	2.513	964	0.009	BV
3	2.687	1108	0.010	VB
4	2.813	4496	0.040	BB
5	3.887	5470	0.048	BV
6	4.440	14873	0.132	VB
7	5.500	2716	0.024	BV
8	6.607	36782	0.326	VB
9	9.713	11132752	98.662	BV
10	12.327	82172	0.728	TBB
		11283713	100.000	

Peak rejection level: 0

Chromaster System Manager Report

Analyzed Date and Time: 2020/03/17 11:16 上午
Reported Date and Time: 2020/03/17 11:34:18 上午
Processed Date and Time: 2020/03/17 11:31 上午
Data Path: C:\WIN32APP\CHROMASTER\TKW\DATA\0121\
Processing Method: KYT

System (acquisition): Sys 1
Series: 0121
Application(data): TKW
Vial Number: 1
Sample Name: 5428
Vial Type: UNK
Injection from this vial: 1 of 1
Volume: 20.0 ul
Sample Description:

Chrom Type: Chromaster Channel : 1

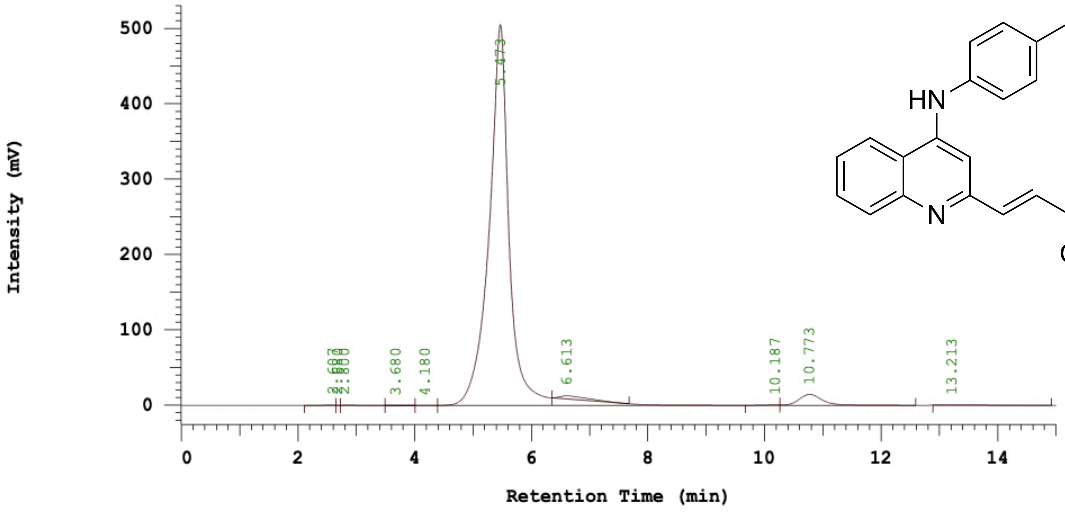

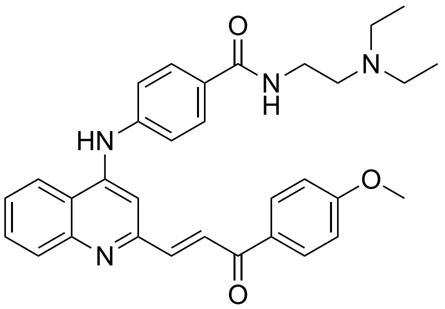

Processing Method: KYT
Method Developer: Hitachi
Pump 1: 5110
Pump 1 Solvent A:
Pump 1 Solvent B:
Pump 1 Solvent C:
Pump 1 Solvent D:
Method Description: MeOH / 10mM KH2PO4 = 50/50 (pH = 2.94)

Chrom Type: Chromaster Channel : 1
Peak Quantitation: AREA
Calculation Method: AREA%

No.	RT	Area	Conc 1	BC
1	2.607	4931	0.038	BV
2	2.680	1045	0.008	VB
3	2.800	16123	0.123	BV
4	3.680	324	0.002	TBB
5	4.180	3268	0.025	VV
6	5.473	12466577	95.389	VV
7	6.613	155121	1.187	TBB
8	10.187	10385	0.079	VV
9	10.773	406201	3.108	VB
10	13.213	5248	0.040	BB
		13069223	100.000	

Peak rejection level: 0

Chromaster System Manager Report

Analyzed Date and Time: 2020/03/13 12:06 下午
Reported Date and Time: 2020/03/13 02:00:27 下午
Processed Date and Time: 2020/03/13 12:21 下午
Data Path: C:\WIN32APP\CHROMASTER\TKW\DATA\0119\
Processing Method: KYT

System (acquisition): Sys 1
Series: 0119
Application(data): TKW
Vial Number: 1
Sample Name: 5429
Vial Type: UNK
Injection from this vial: 1 of 1
Volume: 20.0 ul
Sample Description:

Chrom Type: Chromaster Channel : 1

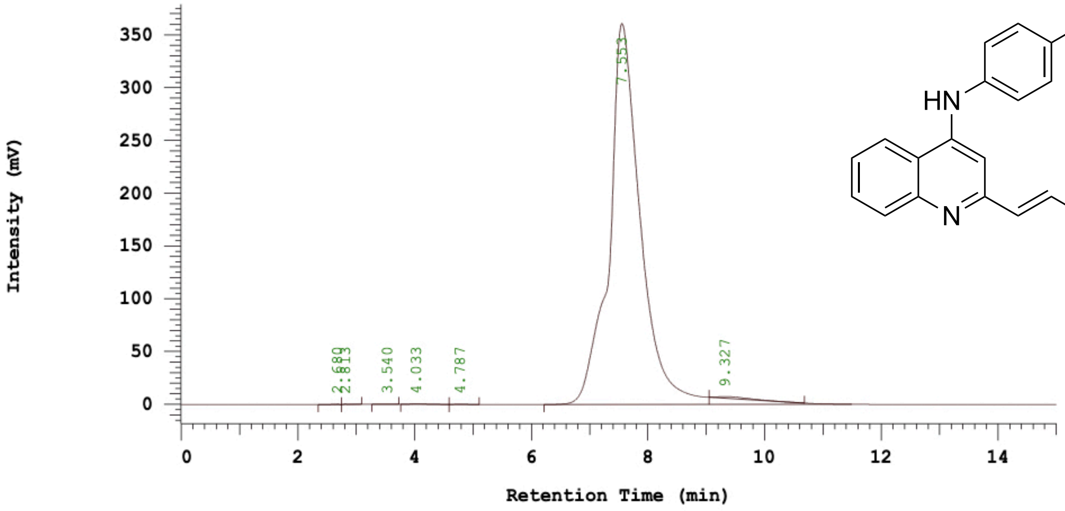

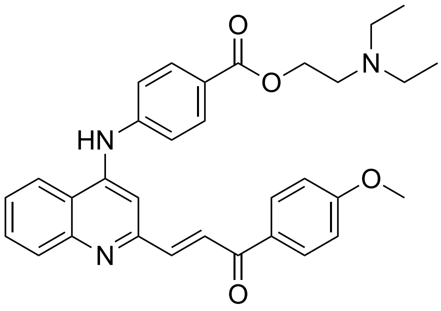

Processing Method: KYT
Method Developer: Hitachi
Pump 1: 5110
Pump 1 Solvent A:
Pump 1 Solvent B:
Pump 1 Solvent C:
Pump 1 Solvent D:
Method Description: MeOH / 10mM KH2PO4 = 50%/50% (pH = 2.64)

Chrom Type: Chromaster Channel : 1
Peak Quantitation: AREA
Calculation Method: AREA%

No.	RT	Area	Conc 1	BC
1	2.680	3777	0.027	BB
2	2.813	3699	0.027	BB
3	3.540	1215	0.009	BB
4	4.033	12913	0.093	BB
5	4.787	2646	0.019	BB
6	7.553	13812465	99.350	BV
7	9.327	66077	0.475	TBB
		13902792	100.000	

Peak rejection level: 0

CSM: TKW Series: 0140 Report Name: original System: Sys 1

Chromaster System Manager Report

Analyzed Date and Time: 2020/03/18 08:30 下午

Reported Date and Time: 2020/03/18 08:46:53 下午

Processed Date and Time: 2020/03/18 08:45 下午

Data Path: C:\WIN32APP\CHROMASTER\TKW\DATA\0140\

Processing Method: KYT

System (acquisition): Sys 1
Application(data): TKW
Sample Name: 5430
Injection from this vial: 1 of 1
Sample Description:

Series: 0140
Vial Number: 1
Vial Type: UNK
Volume: 20.0 ul

Chrom Type: Chromaster Channel : 1

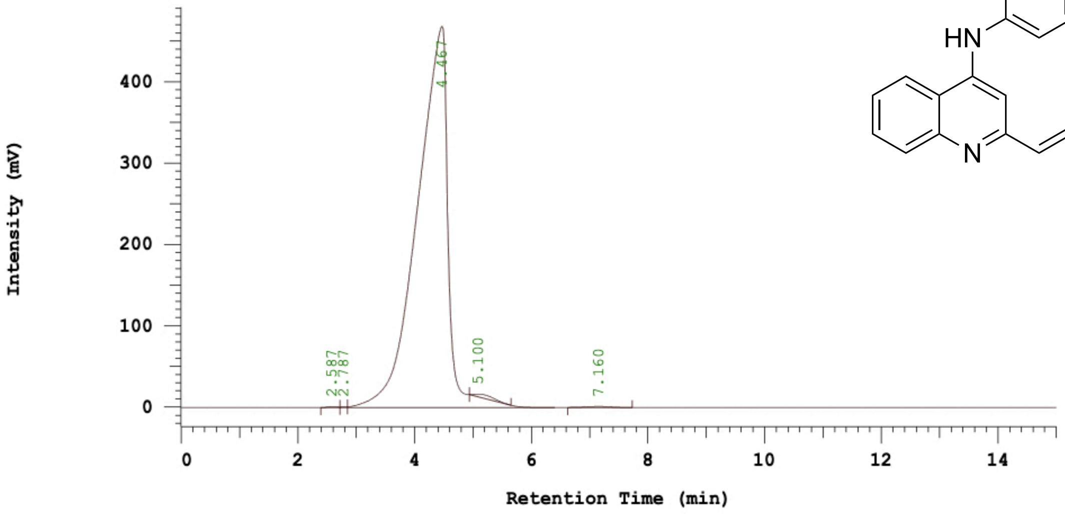

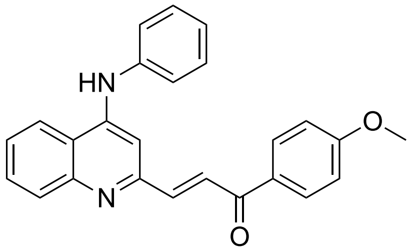

Processing Method: KYT
Method Developer: Hitachi
Pump 1: 5110
Pump 1 Solvent A:
Pump 1 Solvent B:
Pump 1 Solvent C:
Pump 1 Solvent D:
Method Description: MeOH / 10mM KH2PO4 = 80/20 (pH = 3.47)

Chrom Type: Chromaster Channel : 1
Peak Quantitation: AREA
Calculation Method: AREA%

No.	RT	Area	Conc 1	BC
1	2.587	12788	0.074	BV
2	2.787	6747	0.039	VV
3	4.467	17184526	99.103	VV
4	5.100	102368	0.590	TBB
5	7.160	33551	0.193	BB
		17339980	100.000	

Peak rejection level: 0

Chromaster System Manager Report

Analyzed Date and Time: 2020/03/09 02:16 下午 Reported Date and Time: 2020/03/09 04:03:48 下午
Processed Date and Time: 2020/03/09 02:36 下午
Data Path: C:\WIN32APP\CHROMASTER\TKW\DATA\0110\
Processing Method: KYT
System (acquisition): Sys 1 Series: 0110
Application(data): TKW Vial Number: 2
Sample Name: 5431 Vial Type: UNK
Injection from this vial: 1 of 1 Volume: 20.0 ul
Sample Description:

Chrom Type: Chromaster Channel : 1

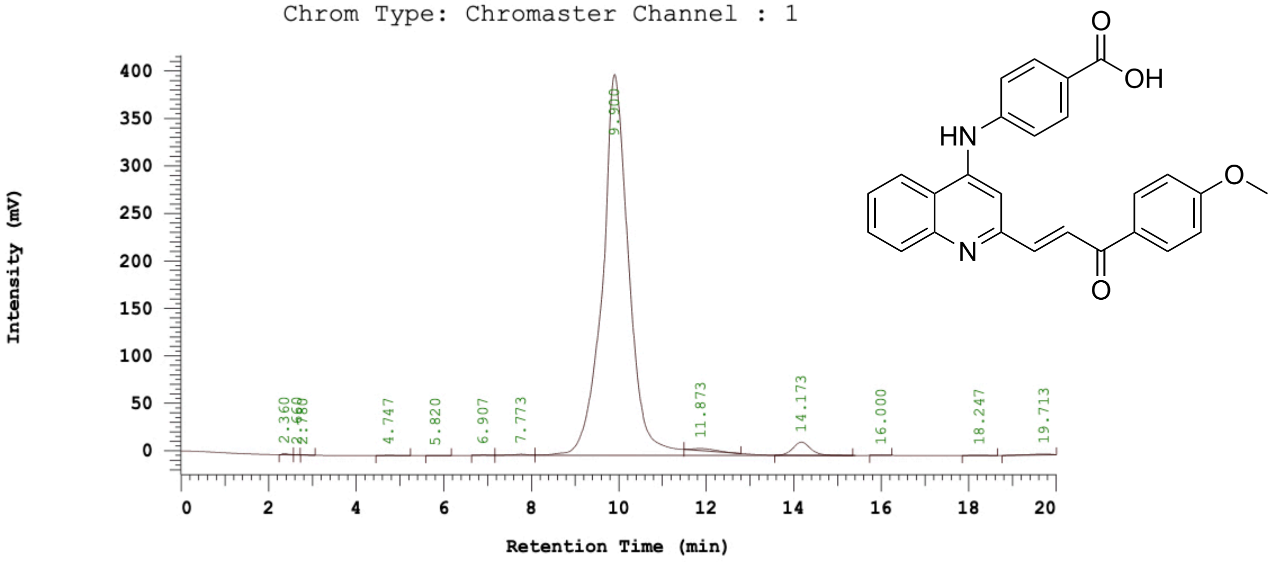

Processing Method: KYT
Method Developer: Hitachi
Pump 1: 5110
Pump 1 Solvent A: Pump 1 Solvent B:
Pump 1 Solvent C: Pump 1 Solvent D:
Method Description: MOH:10mM KH2PO4 = 6/4 (pH=2.88)

Chrom Type: Chromaster Channel : 1
Peak Quantitation: AREA
Calculation Method: AREA%

No.	RT	Area	Conc 1	BC
1	2.360	14047	0.079	BV
2	2.660	3847	0.022	VB
3	2.780	2970	0.017	BB
4	4.747	5133	0.029	BB
5	5.820	1373	0.008	BB
6	6.907	5710	0.032	BV
7	7.773	32009	0.181	VV
8	9.900	17118695	96.695	VV
9	11.873	86413	0.488	TBB
10	14.173	393135	2.221	TBB
11	16.000	1797	0.010	BB
12	18.247	4540	0.026	BB
13	19.713	34198	0.193	BB
		17703867	100.000	

Chromaster System Manager Report

Analyzed Date and Time: 2020/03/18 07:54 下午
Reported Date and Time: 2020/03/18 08:29:16 下午
Processed Date and Time: 2020/03/18 08:09 下午
Data Path: C:\WIN32APP\CHROMASTER\TKW\DATA\0138\
Processing Method: KYT

System (acquisition): Sys 1
Series: 0138
Application(data): TKW
Vial Number: 1
Sample Name: 5433
Vial Type: UNK
Injection from this vial: 1 of 1
Volume: 20.0 ul
Sample Description:

Chrom Type: Chromaster Channel : 1

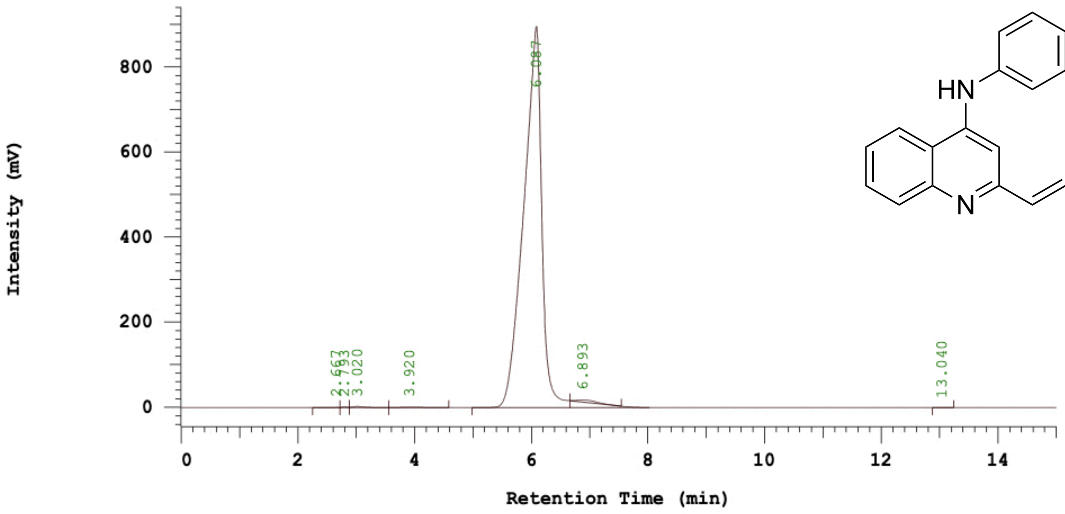

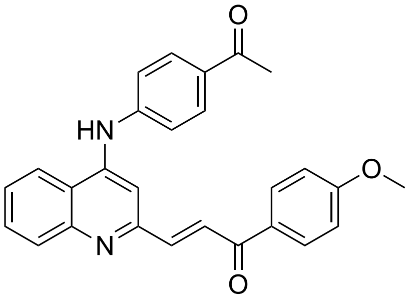

Processing Method: KYT
Method Developer: Hitachi
Pump 1: 5110
Pump 1 Solvent A:
Pump 1 Solvent B:
Pump 1 Solvent C:
Pump 1 Solvent D:
Method Description: MeOH / 10mM KH2PO4 = 80/20 (pH = 3.47)

Chrom Type: Chromaster Channel : 1
Peak Quantitation: AREA
Calculation Method: AREA%

No.	RT	Area	Conc 1	BC
1	2.667	4975	0.025	BB
2	2.793	10077	0.050	BV
3	3.020	36673	0.183	VV
4	3.920	14434	0.072	VB
5	6.087	19811727	99.033	BV
6	6.893	126237	0.631	TBB
7	13.040	1018	0.005	BB
		20005141	100.000	

Peak rejection level: 0

Chromaster System Manager Report

Analyzed Date and Time: 2020/03/18 02:29 下午
Reported Date and Time: 2020/03/18 02:52:45 下午
Processed Date and Time: 2020/03/18 02:49 下午
Data Path: C:\WIN32APP\CHROMASTER\TKW\DATA\0130\
Processing Method: KYT

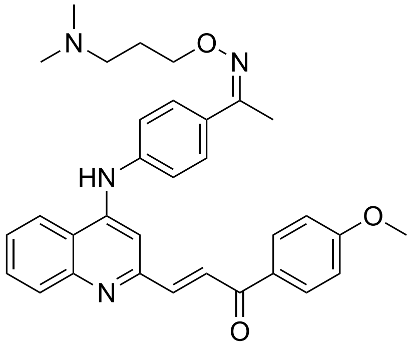

System (acquisition): Sys 1
Application(data): TKW
Sample Name: 5435
Injection from this vial: 1 of 1
Sample Description:

Series: 0130
Vial Number: 1
Vial Type: UNK
Volume: 20.0 ul

Chrom Type: Chromaster Channel : 1

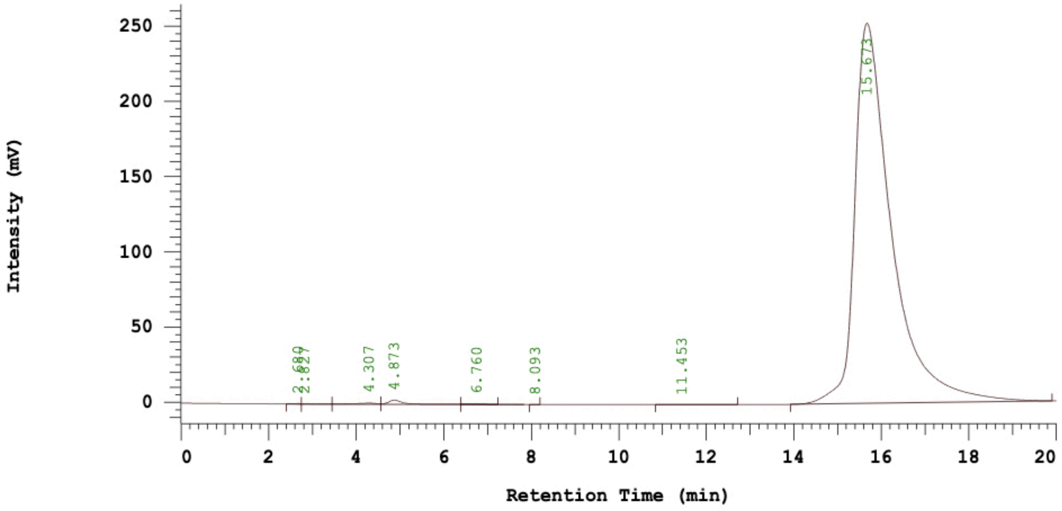

Processing Method: KYT
Method Developer: Hitachi
Pump 1: 5110
Pump 1 Solvent A:
Pump 1 Solvent B:
Pump 1 Solvent C:
Pump 1 Solvent D:
Method Description: MeOH / 10mM KH2PO4 = 50 / 50 (pH = 3.47)

Chrom Type: Chromaster Channel : 1
Peak Quantitation: AREA
Calculation Method: AREA%

No.	RT	Area	Conc 1	BC
1	2.680	5045	0.034	BB
2	2.827	12626	0.085	BV
3	4.307	34824	0.235	VV
4	4.873	75745	0.510	VV
5	6.760	4042	0.027	TBB
6	8.093	80	0.001	BB
7	11.453	2535	0.017	BB
8	15.673	14713840	99.092	BB
		14848737	100.000	

Peak rejection level: 0

Chromaster System Manager Report

Analyzed Date and Time: 2020/03/17 03:25 下午
Reported Date and Time: 2020/03/17 03:52:40 下午
Processed Date and Time: 2020/03/17 03:45 下午
Data Path: C:\WIN32APP\CHROMASTER\TKW\DATA\0126\
Processing Method: KYT

System (acquisition): Sys 1
Series: 0126
Application(data): TKW
Vial Number: 1
Sample Name: 5436
Vial Type: UNK
Injection from this vial: 1 of 1
Volume: 20.0 ul
Sample Description:

Chrom Type: Chromaster Channel : 1

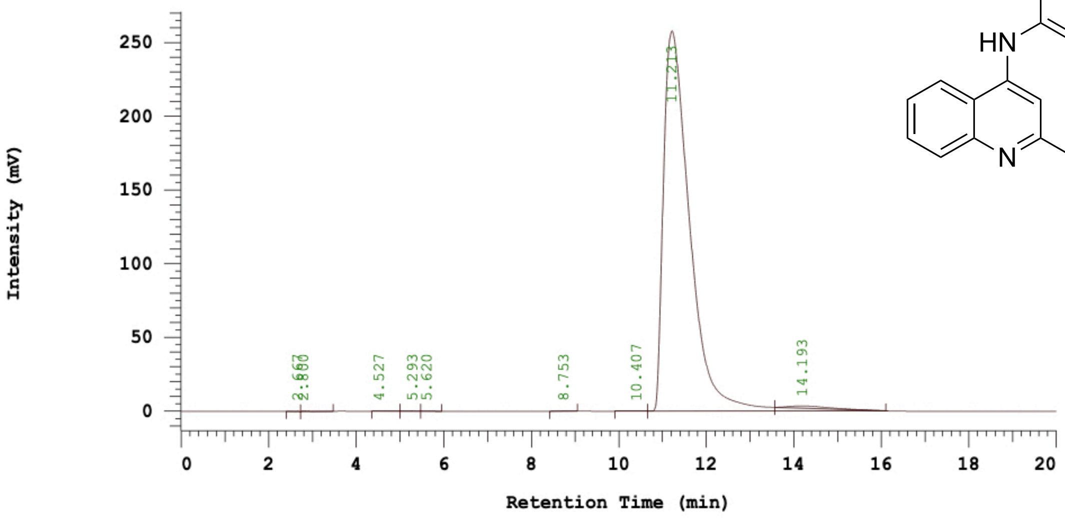

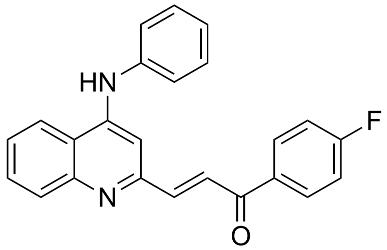

Processing Method: KYT
Method Developer: Hitachi
Pump 1: 5110
Pump 1 Solvent A:
Pump 1 Solvent B:
Pump 1 Solvent C:
Pump 1 Solvent D:
Method Description: MeOH / 10mM KH2PO4 = 60 / 40 (pH = 2.94)

Chrom Type: Chromaster Channel : 1
Peak Quantitation: AREA
Calculation Method: AREA%

No.	RT	Area	Conc 1	BC
1	2.667	2546	0.023	BB
2	2.800	8490	0.078	BB
3	4.527	2259	0.021	BV
4	5.293	4399	0.040	VV
5	5.620	3715	0.034	VB
6	8.753	3612	0.033	BB
7	10.407	1630	0.015	BB
8	11.213	10804203	98.822	BV
9	14.193	102117	0.934	TBB
		10932971	100.000	

Peak rejection level: 0

Chromaster System Manager Report

Analyzed Date and Time: 2020/03/09 11:23 上午
Reported Date and Time: 2020/03/09 04:03:12 下午
Processed Date and Time: 2020/03/09 11:38 上午
Data Path: C:\WIN32APP\CHROMASTER\TKW\DATA\0109\
Processing Method: KYT

System (acquisition): Sys 1
Series: 0109
Application(data): TKW
Vial Number: 1
Sample Name: 5437
Vial Type: UNK
Injection from this vial: 1 of 1
Volume: 20.0 ul
Sample Description:

Chrom Type: Chromaster Channel : 1

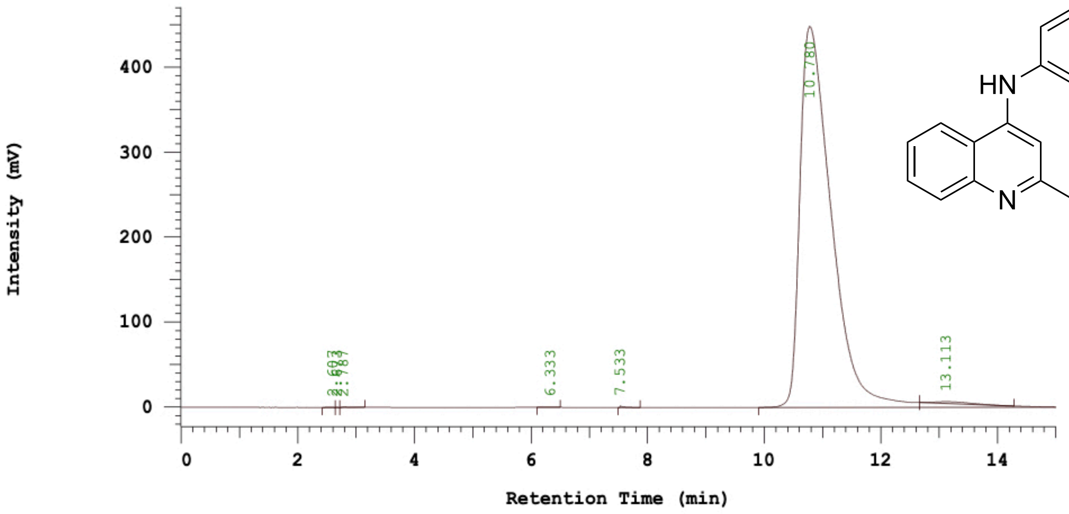

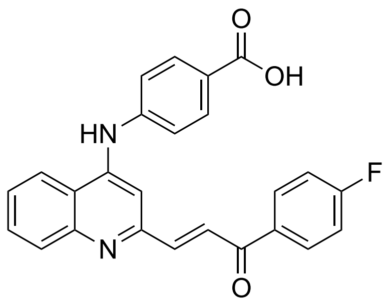

Processing Method: KYT
Method Developer: Hitachi
Pump 1: 5110
Pump 1 Solvent A:
Pump 1 Solvent B:
Pump 1 Solvent C:
Pump 1 Solvent D:
Method Description: MOH:10mM KH2PO4 = 6/4 (pH=3.47)

Chrom Type: Chromaster Channel : 1
Peak Quantitation: AREA
Calculation Method: AREA%

No.	RT	Area	Conc 1	BC
1	2.607	1474	0.009	BV
2	2.673	1036	0.006	VB
3	2.787	6304	0.037	BB
4	6.333	981	0.006	BB
5	7.533	6560	0.038	BB
6	10.780	17034394	99.311	BV
7	13.113	101778	0.593	TBB
		17152527	100.000	

Peak rejection level: 0

Chromaster System Manager Report

Analyzed Date and Time: 2020/03/18 08:12 下午

Reported Date and Time: 2020/03/18 08:29:40 下午

Processed Date and Time: 2020/03/18 08:27 下午

Data Path: C:\WIN32APP\CHROMASTER\TKW\DATA\0139\

Processing Method: KYT

System (acquisition): Sys 1

Series: 0139

Application(data): TKW

Vial Number: 1

Sample Name: 3438

Vial Type: UNK

Injection from this vial: 1 of 1

Volume: 20.0 ul

Sample Description:

Chrom Type: Chromaster Channel : 1

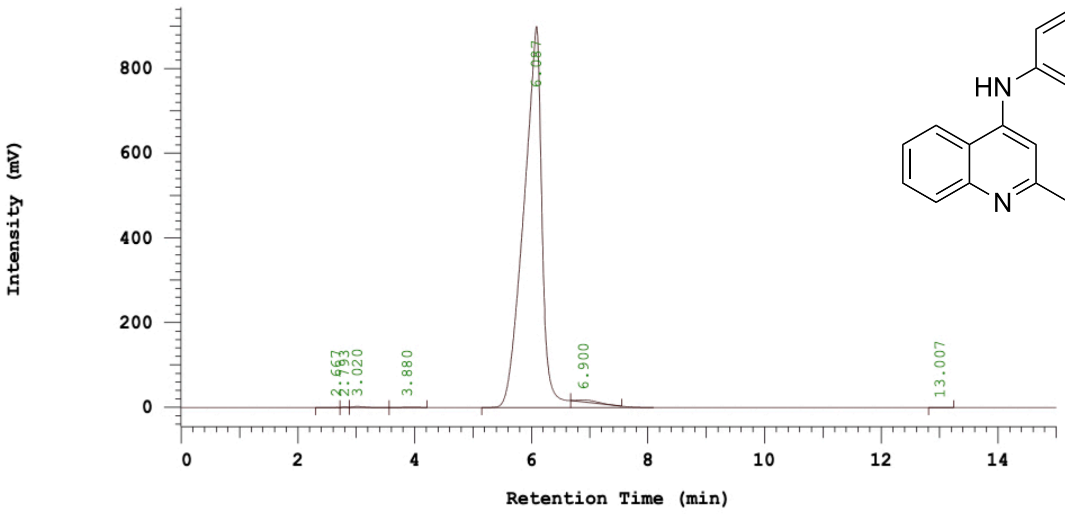

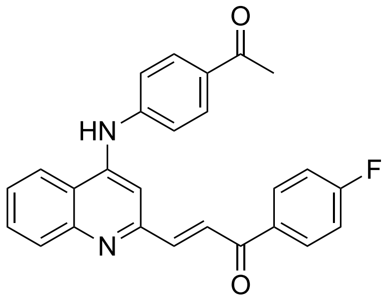

Processing Method: KYT

Method Developer: Hitachi

Pump 1: 5110

Pump 1 Solvent A:

Pump 1 Solvent B:

Pump 1 Solvent C:

Pump 1 Solvent D:

Method Description: MeOH / 10mM KH2PO4 = 80/20 (pH = 3.47)

Chrom Type: Chromaster Channel : 1

Peak Quantitation: AREA

Calculation Method: AREA%

No.	RT	Area	Conc 1	BC
1	2.667	4272	0.021	BB
2	2.793	9853	0.049	BV
3	3.020	35140	0.174	VB
4	3.880	7130	0.035	BB
5	6.087	20035274	99.087	BV
6	6.900	126775	0.627	TBB
7	13.007	1353	0.007	BB
		20219797	100.000	

Peak rejection level: 0

Chromaster System Manager Report

Analyzed Date and Time: 2020/03/13 12:22 下午
Reported Date and Time: 2020/03/13 02:00:28 下午
Processed Date and Time: 2020/03/13 12:37 下午
Data Path: C:\WIN32APP\CHROMASTER\TKW\DATA\0119\
Processing Method: KYT

System (acquisition): Sys 1
Series: 0119
Application(data): TKW
Vial Number: 2
Sample Name: 5439
Vial Type: UNK
Injection from this vial: 1 of 1
Volume: 20.0 ul
Sample Description:

Chrom Type: Chromaster Channel : 1

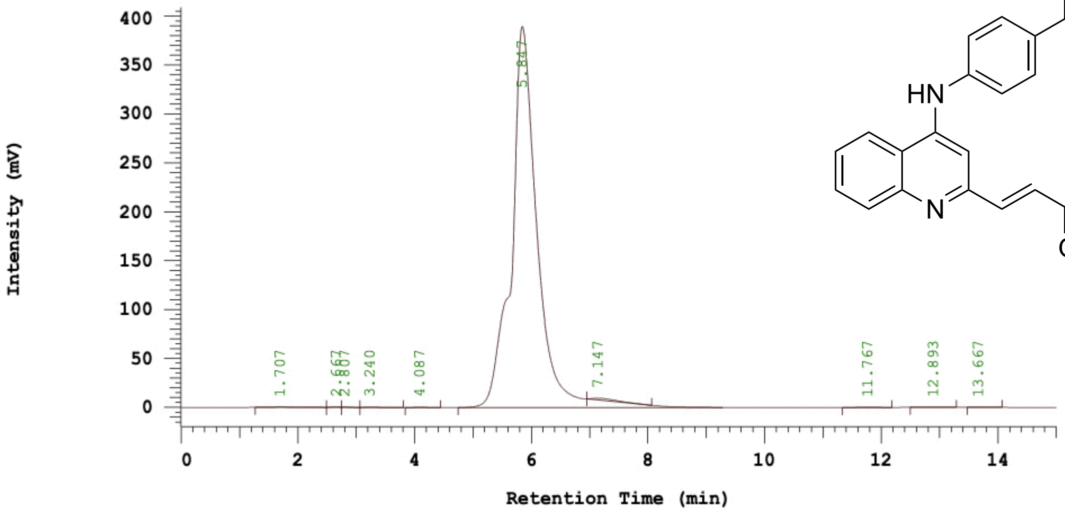

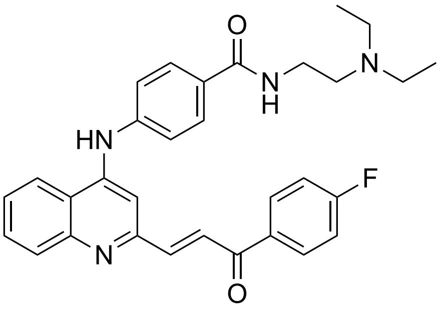

Processing Method: KYT
Method Developer: Hitachi
Pump 1: 5110
Pump 1 Solvent A:
Pump 1 Solvent B:
Pump 1 Solvent C:
Pump 1 Solvent D:
Method Description: MeOH / 10mM KH2PO4 = 50%/50% (pH = 2.64)

Chrom Type: Chromaster Channel : 1
Peak Quantitation: AREA
Calculation Method: AREA%

No.	RT	Area	Conc 1	BC
1	1.707	10808	0.090	BB
2	2.667	7049	0.058	BV
3	2.807	3815	0.032	VV
4	3.240	5194	0.043	VB
5	4.087	4597	0.038	BB
6	5.847	11945354	99.117	BV
7	7.147	63643	0.528	TBB
8	11.767	5972	0.050	BB
9	12.893	2737	0.023	BB
10	13.667	2624	0.022	BB
		12051793	100.000	

Peak rejection level: 0

Chromaster System Manager Report

Analyzed Date and Time: 2020/03/13 12:38 下午
Reported Date and Time: 2020/03/13 02:00:28 下午
Processed Date and Time: 2020/03/13 12:53 下午
Data Path: C:\WIN32APP\CHROMASTER\TKW\DATA\0119\
Processing Method: KYT

System (acquisition): Sys 1
Series: 0119
Application(data): TKW
Vial Number: 3
Sample Name: 5440
Vial Type: UNK
Injection from this vial: 1 of 1
Volume: 20.0 ul
Sample Description:

Chrom Type: Chromaster Channel : 1

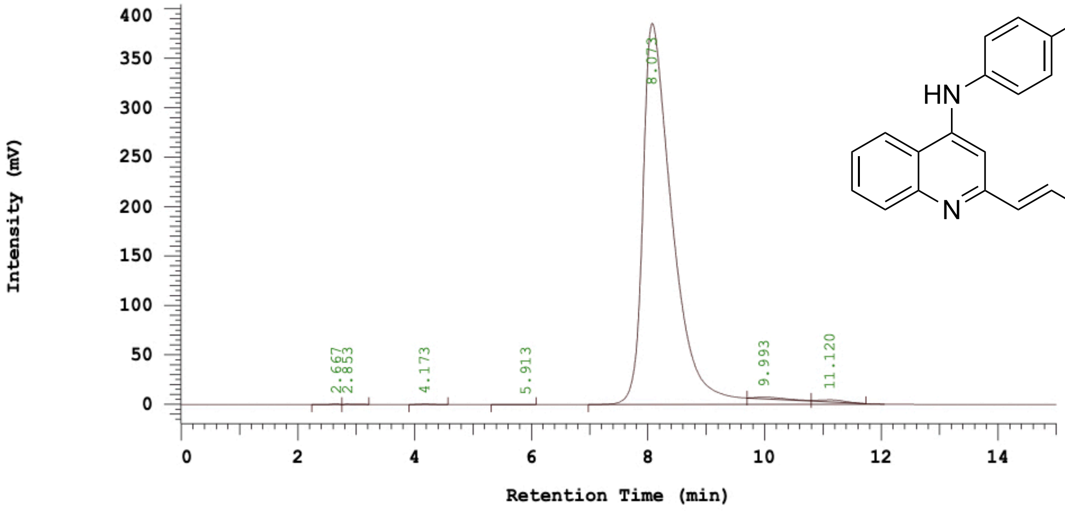

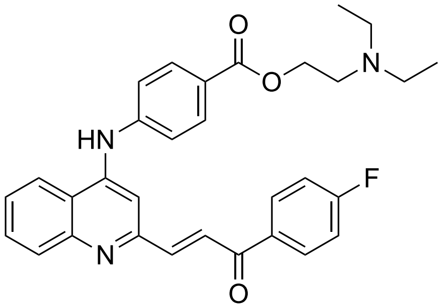

Processing Method: KYT
Method Developer: Hitachi
Pump 1: 5110
Pump 1 Solvent A:
Pump 1 Solvent B:
Pump 1 Solvent C:
Pump 1 Solvent D:
Method Description: MeOH / 10mM KH2PO4 = 50%/50% (pH = 2.64)

Chrom Type: Chromaster Channel : 1
Peak Quantitation: AREA
Calculation Method: AREA%

No.	RT	Area	Conc 1	BC
1	2.667	6793	0.048	BB
2	2.853	5276	0.038	BB
3	4.173	6644	0.047	BB
4	5.913	1198	0.009	BB
5	8.073	13883088	98.896	BV
6	9.993	66283	0.472	TBV
7	11.120	68847	0.490	TVB
		14038129	100.000	

Peak rejection level: 0

Chromaster System Manager Report

Analyzed Date and Time: 2020/03/13 01:27 下午 | Reported Date and Time: 2020/03/13 02:00:28 下午

Processed Date and Time: 2020/03/13 01:42 下午

Data Path: C:\WIN32APP\CHROMASTER\TKW\DATA\0119\
Processing Method: KYT

System (acquisition): Sys 1 | Series: 0119
Application(data): TKW | Vial Number: 6
Sample Name: 5441 | Vial Type: UNK
Injection from this vial: 1 of 1 | Volume: 20.0 ul
Sample Description:

Chrom Type: Chromaster Channel : 1

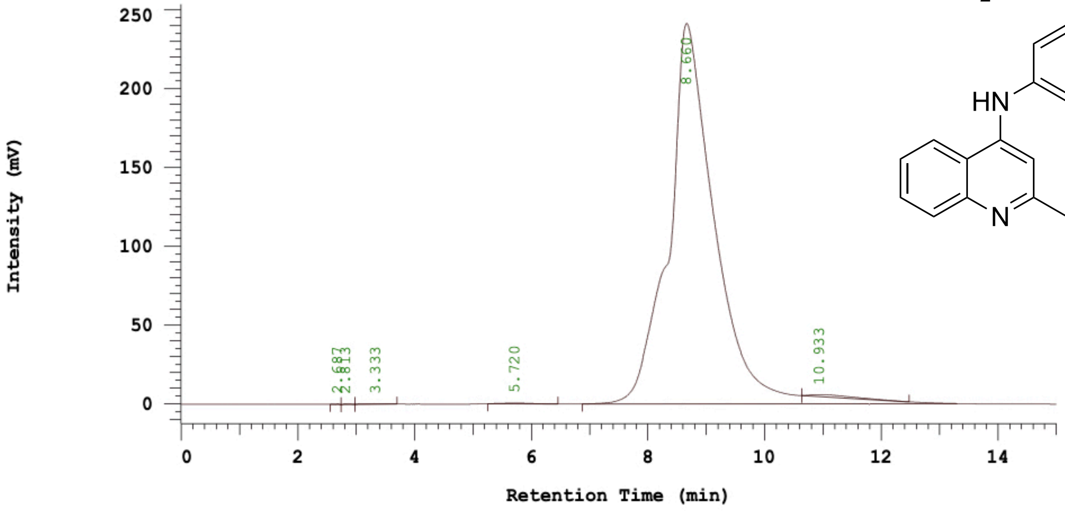

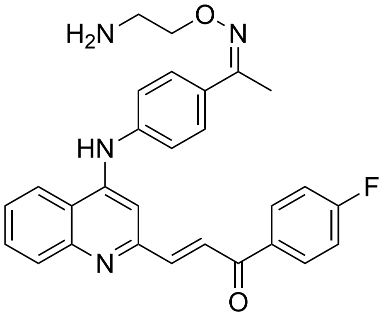

Processing Method: KYT
Method Developer: Hitachi
Pump 1: 5110
Pump 1 Solvent A: Pump 1 Solvent B:
Pump 1 Solvent C: Pump 1 Solvent D:
Method Description: MeOH / 10mM KH2PO4 = 50%/50% (pH = 2.64)

Chrom Type: Chromaster Channel : 1
Peak Quantitation: AREA
Calculation Method: AREA%

No.	RT	Area	Conc 1	BC
1	2.687	1718	0.013	BB
2	2.813	4283	0.032	BV
3	3.333	5970	0.044	VB
4	5.720	14972	0.111	BB
5	8.660	13370652	99.228	BV
6	10.933	77132	0.572	TBB
		13474727	100.000	

Peak rejection level: 0

Chromaster System Manager Report

Analyzed Date and Time: 2020/03/17 04:31 下午   Reported Date and Time: 2020/03/17 04:46:47 下午

Processed Date and Time: 2020/03/17 04:46 下午

Data Path: C:\WIN32APP\CHROMASTER\TKW\DATA\0128\

Processing Method: KYT

System (acquisition): Sys 1   Series: 0128

Application(data): TKW   Vial Number: 1

Sample Name: 5443   Vial Type: UNK

Injection from this vial: 1 of 1   Volume: 20.0 ul

Sample Description:

Chrom Type: Chromaster Channel : 1

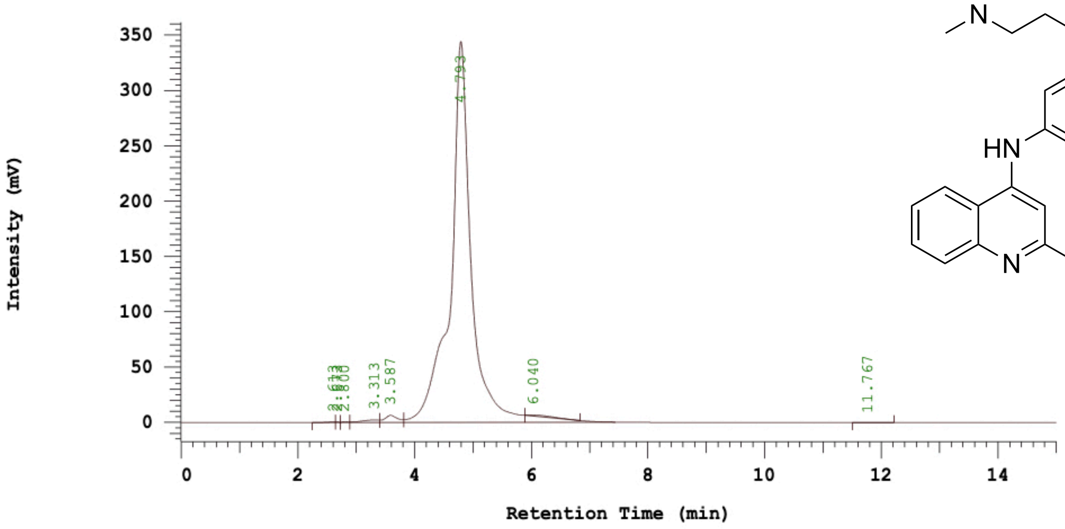

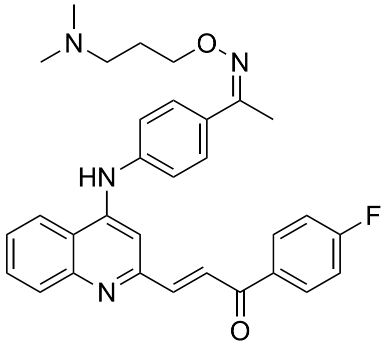

Processing Method: KYT

Method Developer: Hitachi

Pump 1: 5110

Pump 1 Solvent A: Pump 1 Solvent B:

Pump 1 Solvent C: Pump 1 Solvent D:

Method Description: MeOH / 10mM KH2PO4 = 60 / 40 (pH = 2.94)

Chrom Type: Chromaster Channel : 1

Peak Quantitation: AREA

Calculation Method: AREA%

No.	RT	Area	Conc 1	BC
1	2.613	4541	0.051	BV
2	2.673	2055	0.023	VV
3	2.800	5635	0.064	VV
4	3.313	46707	0.530	VV
5	3.587	98455	1.116	VV
6	4.793	8624194	97.775	VV
7	6.040	36294	0.411	TBB
8	11.767	2591	0.029	BB
		8820472	100.000	

Peak rejection level: 0

Chromaster System Manager Report

Analyzed Date and Time: 2020/03/18 06:36 下午
Reported Date and Time: 2020/03/18 07:02:54 下午
Processed Date and Time: 2020/03/18 06:51 下午
Data Path: C:\WIN32APP\CHROMASTER\TKW\DATA\0134\
Processing Method: KYT

System (acquisition): Sys 1
Application(data): TKW
Sample Name: 5444
Injection from this vial: 1 of 1
Sample Description:
Series: 0134
Vial Number: 2
Vial Type: UNK
Volume: 20.0 ul

Chrom Type: Chromaster Channel : 1

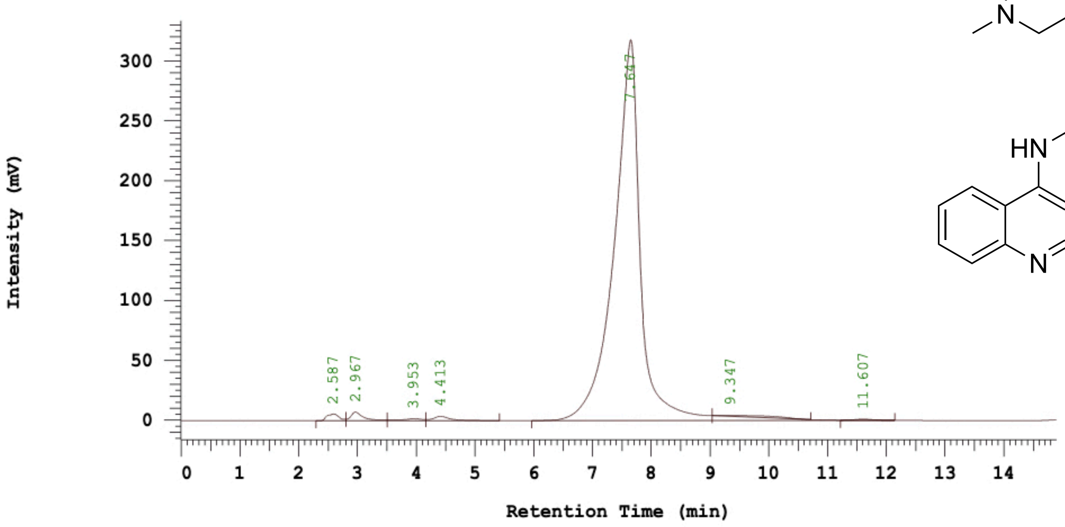

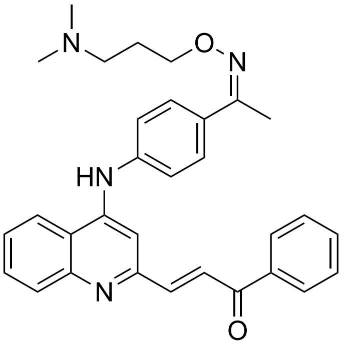

Processing Method: KYT
Method Developer: Hitachi
Pump 1: 5110
Pump 1 Solvent A:
Pump 1 Solvent B:
Pump 1 Solvent C:
Pump 1 Solvent D:
Method Description: MeOH / 10mM KH2PO4 = 60/40 (pH = 3.47)

Chrom Type: Chromaster Channel : 1
Peak Quantitation: AREA
Calculation Method: AREA%

No.	RT	Area	Conc 1	BC
1	2.587	78394	0.753	BV
2	2.967	106846	1.027	VV
3	3.953	39261	0.377	VV
4	4.413	70740	0.680	VB
5	7.647	10007937	96.157	BV
6	9.347	83199	0.799	TBB
7	11.607	21515	0.207	TBB
		10407892	100.000	

Peak rejection level: 0

Chromaster System Manager Report

Analyzed Date and Time: 2020/03/09 12:43 下午
Reported Date and Time: 2020/03/09 04:03:13 下午
Processed Date and Time: 2020/03/09 12:58 下午
Data Path: C:\WIN32APP\CHROMASTER\TKW\DATA\0109\
Processing Method: KYT

System (acquisition): Sys 1
Series: 0109
Application(data): TKW
Vial Number: 6
Sample Name: 5445
Vial Type: UNK
Injection from this vial: 1 of 1
Volume: 20.0 ul
Sample Description:

Chrom Type: Chromaster Channel : 1

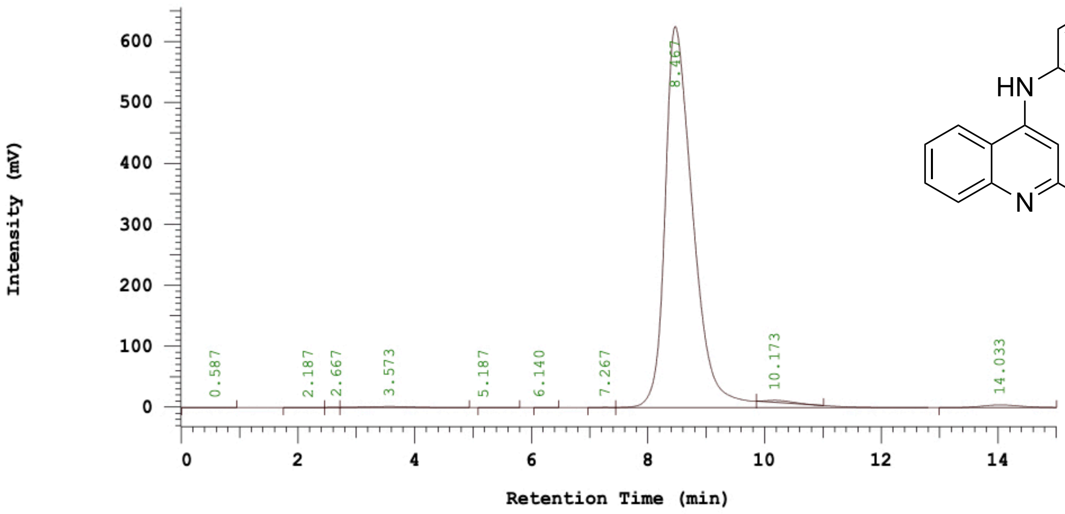

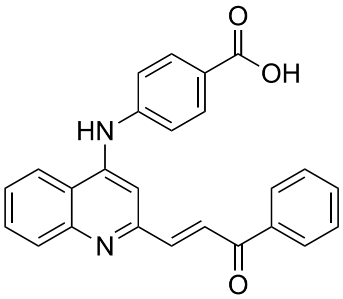

Processing Method: KYT
Method Developer: Hitachi
Pump 1: 5110
Pump 1 Solvent A:
Pump 1 Solvent B:
Pump 1 Solvent C:
Pump 1 Solvent D:
Method Description: MOH:10mM KH2PO4 = 6/4 (pH=3.47)

Chrom Type: Chromaster Channel : 1
Peak Quantitation: AREA
Calculation Method: AREA%

No.	RT	Area	Conc 1	BC
1	0.587	2664	0.012	BB
2	2.187	3219	0.015	BV
3	2.667	3558	0.016	VV
4	3.573	85008	0.394	VB
5	5.187	1487	0.007	BB
6	6.140	638	0.003	BB
7	7.267	3492	0.016	BV
8	8.467	21161310	98.100	VV
9	10.173	117139	0.543	TBB
10	14.033	192741	0.894	BB
		21571256	100.000	

Peak rejection level: 0

Chromaster System Manager Report

Analyzed Date and Time: 2020/03/09 12:59 下午

Reported Date and Time: 2020/03/09 04:03:13 下午

Processed Date and Time: 2020/03/09 01:14 下午

Data Path: C:\WIN32APP\CHROMASTER\TKW\DATA\0109\

Processing Method: KYT

System (acquisition): Sys 1

Series: 0109

Application(data): TKW

Vial Number: 7

Sample Name: 5446

Vial Type: UNK

Injection from this vial: 1 of 1

Volume: 20.0 ul

Sample Description:

Chrom Type: Chromaster Channel : 1

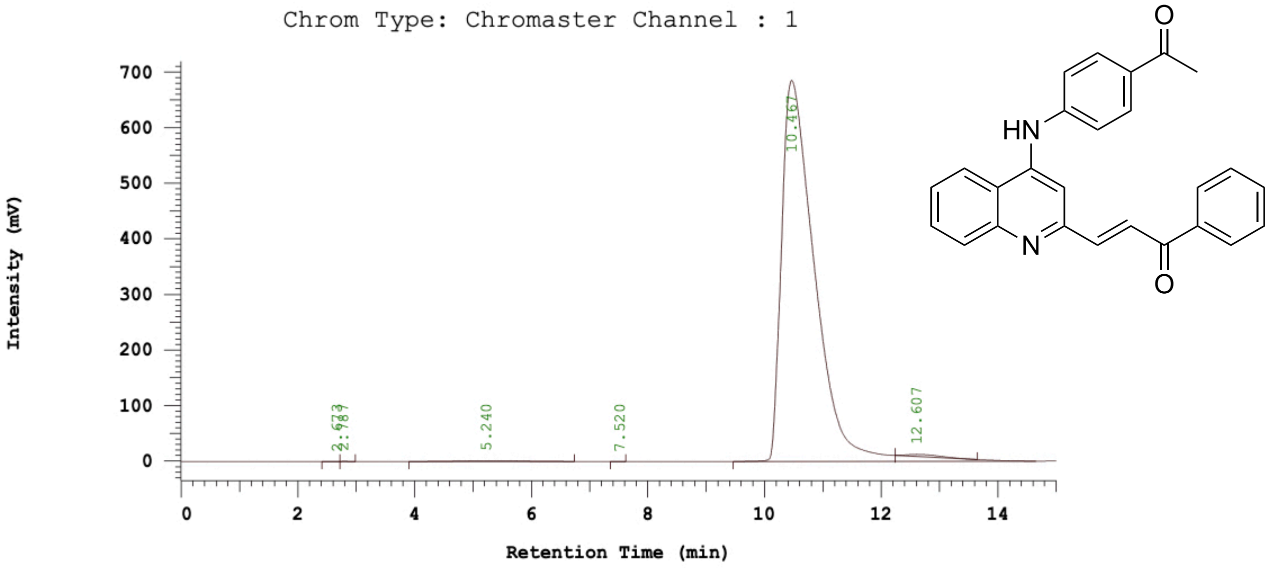

Processing Method: KYT

Method Developer: Hitachi

Pump 1: 5110

Pump 1 Solvent A:

Pump 1 Solvent B:

Pump 1 Solvent C:

Pump 1 Solvent D:

Method Description: MOH:10mM KH2PO4 = 6/4 (pH=3.47)

Chrom Type: Chromaster Channel : 1

Peak Quantitation: AREA

Calculation Method: AREA%

No.	RT	Area	Conc 1	BC
1	2.673	2478	0.009	BB
2	2.787	3224	0.012	BB
3	5.240	112340	0.409	BB
4	7.520	337	0.001	BB
5	10.467	27210636	98.986	BV
6	12.607	160246	0.583	TBB
		27489261	100.000	

Peak rejection level: 0

Chromaster System Manager Report

Analyzed Date and Time: 2020/03/17 03:46 下午   Reported Date and Time: 2020/03/17 04:03:05 下午

Processed Date and Time: 2020/03/17 04:02 下午

Data Path: C:\WIN32APP\CHROMASTER\TKW\DATA\0126\

Processing Method: KYT

System (acquisition): Sys 1   Series: 0126

Application(data): TKW   Vial Number: 2

Sample Name: 5447   Vial Type: UNK

Injection from this vial: 1 of 1   Volume: 20.0 ul

Sample Description:

Chrom Type: Chromaster Channel : 1

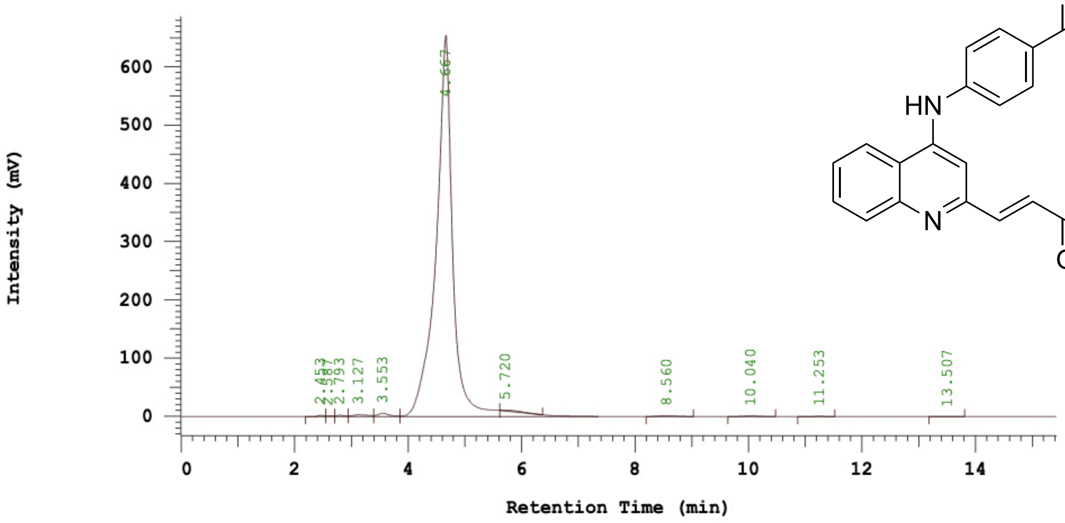

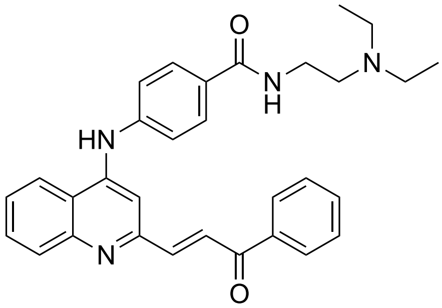

Processing Method: KYT

Method Developer: Hitachi

Pump 1: 5110

Pump 1 Solvent A: Pump 1 Solvent B:

Pump 1 Solvent C: Pump 1 Solvent D:

Method Description: MeOH / 10mM KH2PO4 = 60 / 40 (pH = 2.94)

Chrom Type: Chromaster Channel : 1

Peak Quantitation: AREA

Calculation Method: AREA%

No.	RT	Area	Conc 1	BC
1	2.453	17620	0.125	BV
2	2.587	14291	0.101	VV
3	2.793	25400	0.180	VV
4	3.127	60592	0.430	VV
5	3.553	79780	0.566	VV
6	4.667	13807710	97.921	VV
7	5.720	44266	0.314	TBB
8	8.560	27336	0.194	BB
9	10.040	18754	0.133	BB
10	11.253	3777	0.027	BB
11	13.507	1342	0.010	BB
		14100868	100.000	

Peak rejection level: 0

Chromaster System Manager Report

Analyzed Date and Time: 2020/03/13 01:11 下午 | Reported Date and Time: 2020/03/13 02:00:28 下午

Processed Date and Time: 2020/03/13 01:26 下午

Data Path: C:\WIN32APP\CHROMASTER\TKW\DATA\0119\

Processing Method: KYT

System (acquisition): Sys 1 | Series: 0119

Application(data): TKW | Vial Number: 5

Sample Name: 5448 | Vial Type: UNK

Injection from this vial: 1 of 1 | Volume: 20.0 ul

Sample Description:

Chrom Type: Chromaster Channel : 1

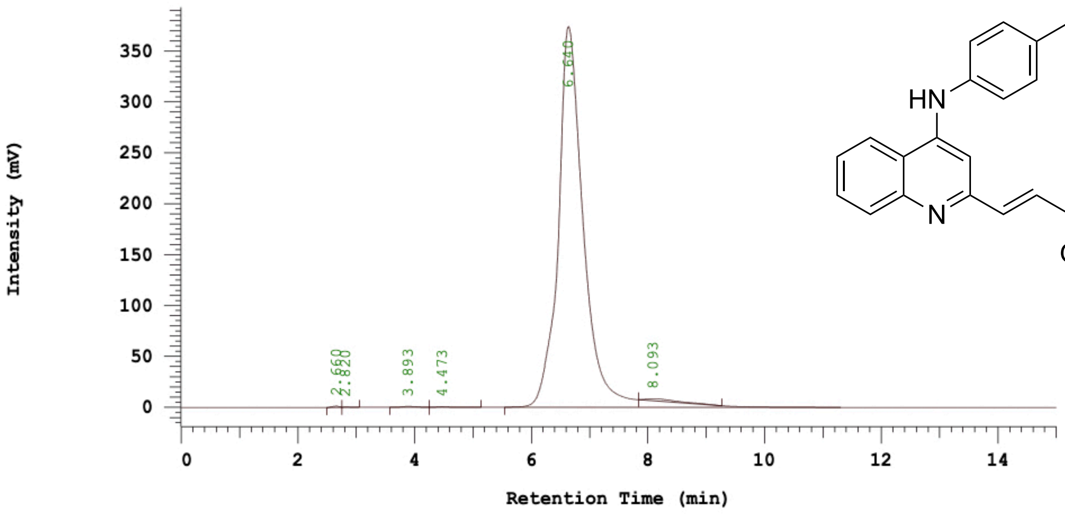

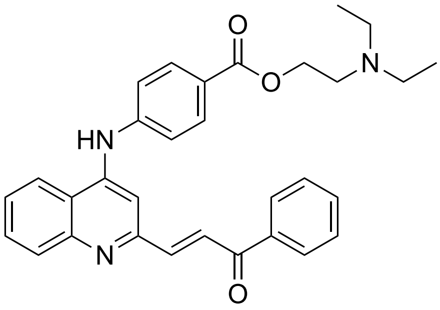

Processing Method: KYT
Method Developer: Hitachi
Pump 1: 5110

Pump 1 Solvent A: | Pump 1 Solvent B:

Pump 1 Solvent C: | Pump 1 Solvent D:

Method Description: MeOH / 10mM KH2PO4 = 50%/50% (pH = 2.64)

Chrom Type: Chromaster Channel : 1

Peak Quantitation: AREA
Calculation Method: AREA%

No.	RT	Area	Conc 1	BC
1	2.660	11232	0.096	BV
2	2.820	4130	0.035	VB
3	3.893	15576	0.134	BB
4	4.473	8685	0.074	BB
5	6.640	11534144	98.907	BV
6	8.093	87862	0.753	TBB
		11661629	100.000	

Peak rejection level: 0

Chromaster System Manager Report

Analyzed Date and Time: 2020/03/17 01:38 下午
Reported Date and Time: 2020/03/17 01:56:12 下午
Processed Date and Time: 2020/03/17 01:53 下午
Data Path: C:\WIN32APP\CHROMASTER\TKW\DATA\0122\
Processing Method: KYT

System (acquisition): Sys 1
Application(data): TKW
Sample Name: 5450
Injection from this vial: 1 of 1
Sample Description:

Series: 0122
Vial Number: 1
Vial Type: UNK
Volume: 20.0 ul

Chrom Type: Chromaster Channel : 1

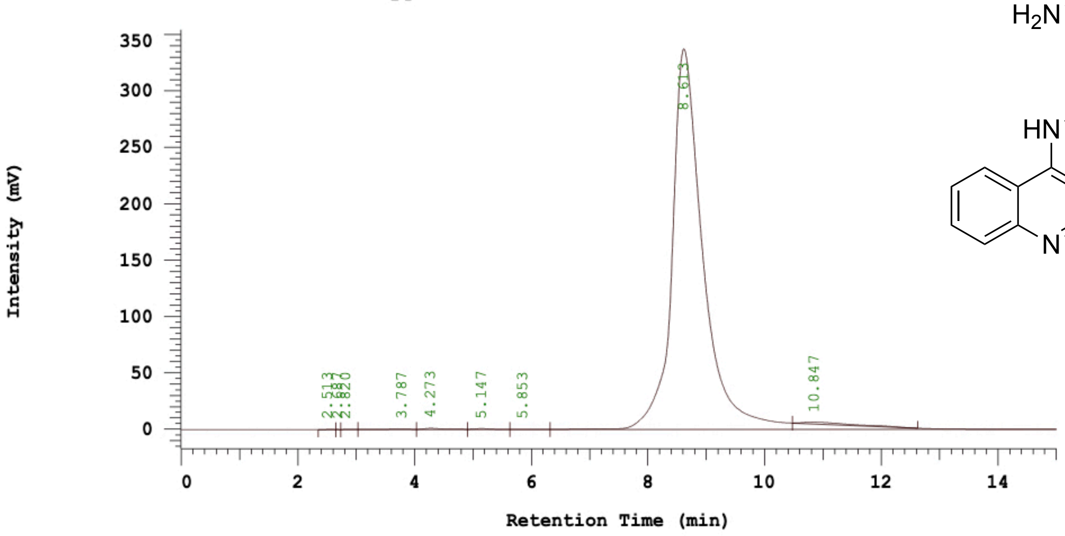

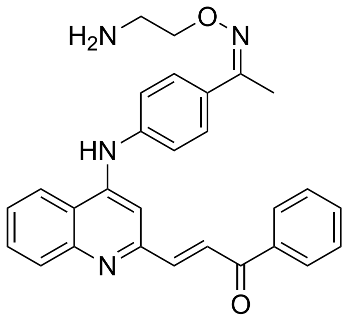

Processing Method: KYT
Method Developer: Hitachi
Pump 1: 5110
Pump 1 Solvent A:
Pump 1 Solvent B:
Pump 1 Solvent C:
Pump 1 Solvent D:
Method Description: MeOH / 10mM KH2PO4 = 50 / 50 (pH = 2.94)

Chrom Type: Chromaster Channel : 1
Peak Quantitation: AREA
Calculation Method: AREA%

No.	RT	Area	Conc 1	BC
1	2.513	1572	0.012	BV
2	2.687	789	0.006	VB
3	2.820	4795	0.036	BV
4	3.787	23855	0.181	VV
5	4.273	35943	0.273	VV
6	5.147	22762	0.173	VV
7	5.853	11107	0.084	VV
8	8.613	12944869	98.417	VV
9	10.847	107369	0.816	TBB
		13153061	100.000	

Peak rejection level: 0

Chromaster System Manager Report

Analyzed Date and Time: 2020/03/09 02:37 下午
Reported Date and Time: 2020/03/09 04:03:48 下午
Processed Date and Time: 2020/03/09 02:57 下午
Data Path: C:\WIN32APP\CHROMASTER\TKW\DATA\0110\
Processing Method: KYT

System (acquisition): Sys 1
Series: 0110
Application(data): TKW
Vial Number: 3
Sample Name: H aniline
Vial Type: UNK
Injection from this vial: 1 of 1
Volume: 20.0 ul
Sample Description:

Chrom Type: Chromaster Channel : 1

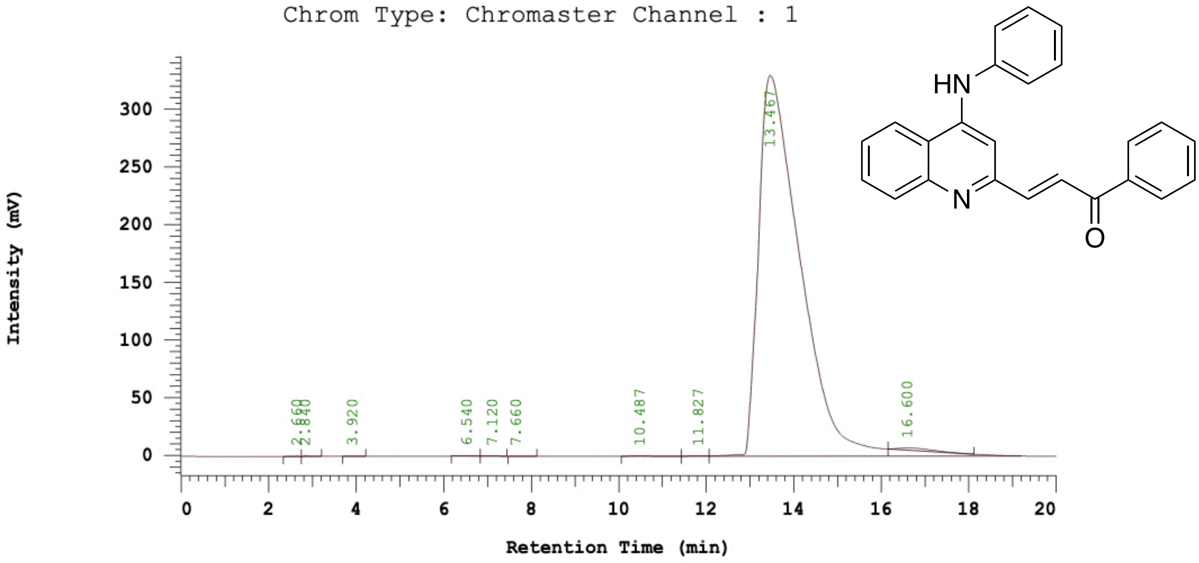

Processing Method: KYT
Method Developer: Hitachi
Pump 1: 5110
Pump 1 Solvent A:
Pump 1 Solvent B:
Pump 1 Solvent C:
Pump 1 Solvent D:
Method Description: MOH:10mM KH2PO4 = 6/4 (pH=2.88)

Chrom Type: Chromaster Channel : 1
Peak Quantitation: AREA
Calculation Method: AREA%

No.	RT	Area	Conc 1	BC
1	2.660	4422	0.020	BB
2	2.840	5937	0.027	BB
3	3.920	1911	0.009	BB
4	6.540	6697	0.030	BV
5	7.120	9722	0.044	VB
6	7.660	1831	0.008	BB
7	10.487	9322	0.042	BB
8	11.827	9419	0.043	BV
9	13.467	21966773	99.271	VV
10	16.600	112151	0.507	TBB
		22128185	100.000	

Peak rejection level: 0

Nuclear magnetic resonance (^{1}H and ^{13}C) spectra.

NMR (^{1}H and ^{13}C) spectra were recorded on a Varian Gemini 200 spectrometer or Varian-Unity-400 spectrometer. Chemical shifts were expressed in parts per million (δ) with tetramethylsilane (TMS) as an internal standard.

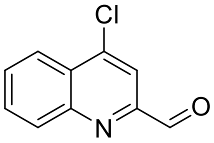
Cl
N
O

8

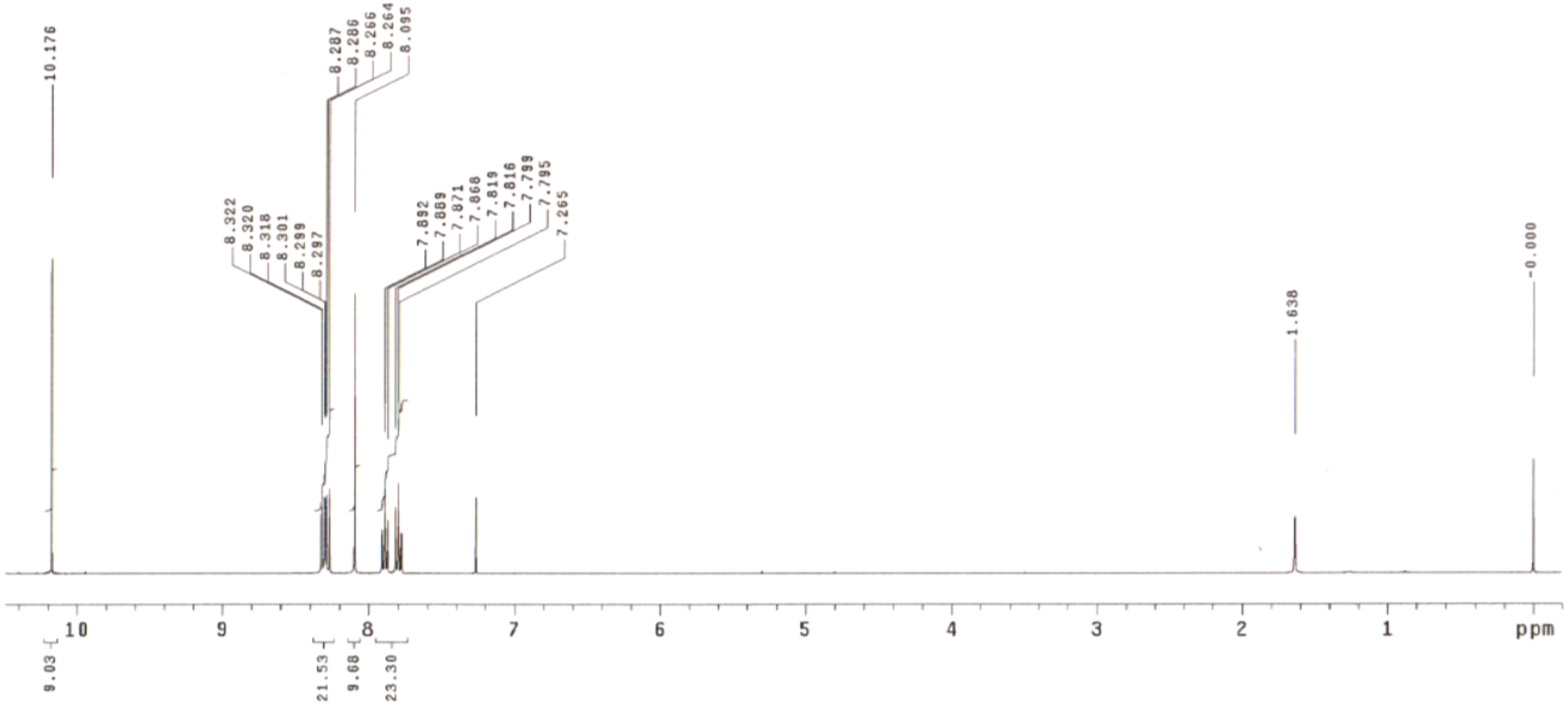
10.176
8.322
8.320
8.318
8.301
8.299
8.297
8.287
8.286
8.266
8.264
8.095
7.892
7.889
7.871
7.868
7.819
7.816
7.799
7.795
7.265
1.638
-0.000
10
9
8
7
6
5
4
3
2
1
ppm
9.03
21.53
9.68
23.30

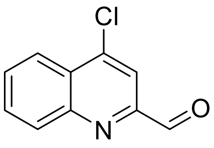

8

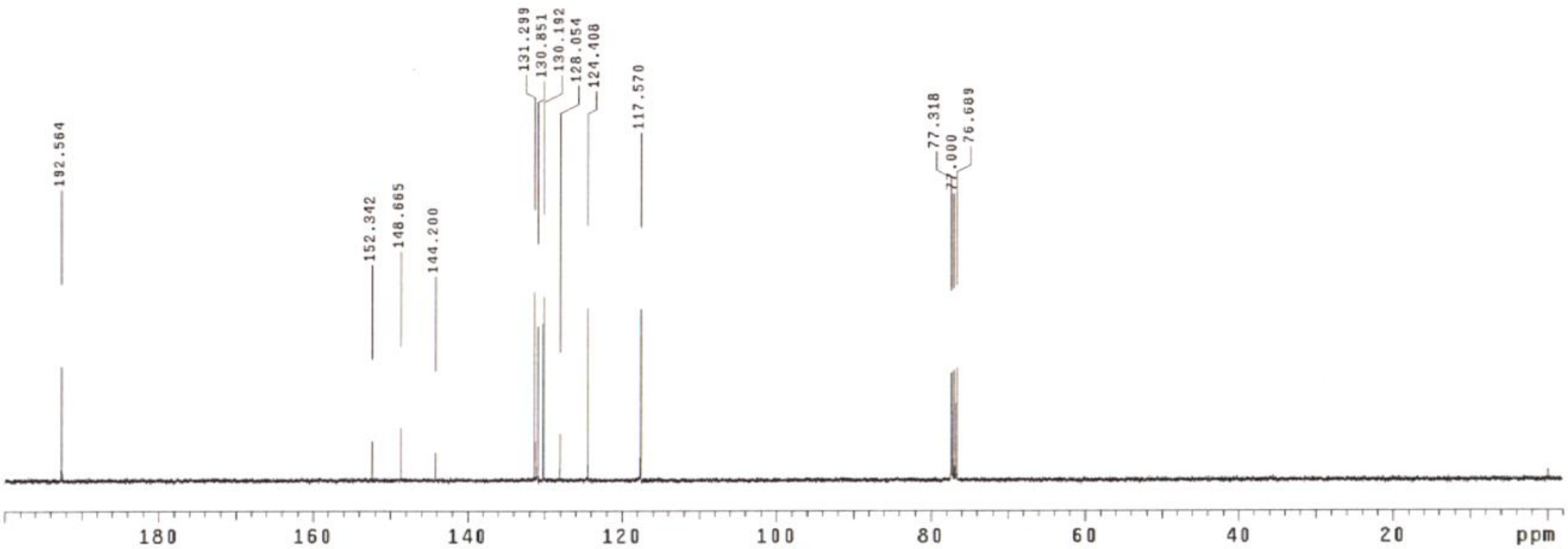
192.564
152.342
148.665
144.200
131.299
130.851
130.192
128.054
124.408
117.570
77.318
77.000
76.689
180
160
140
120
100
80
60
40
20
ppm

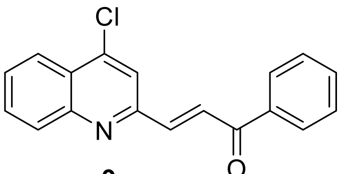

9

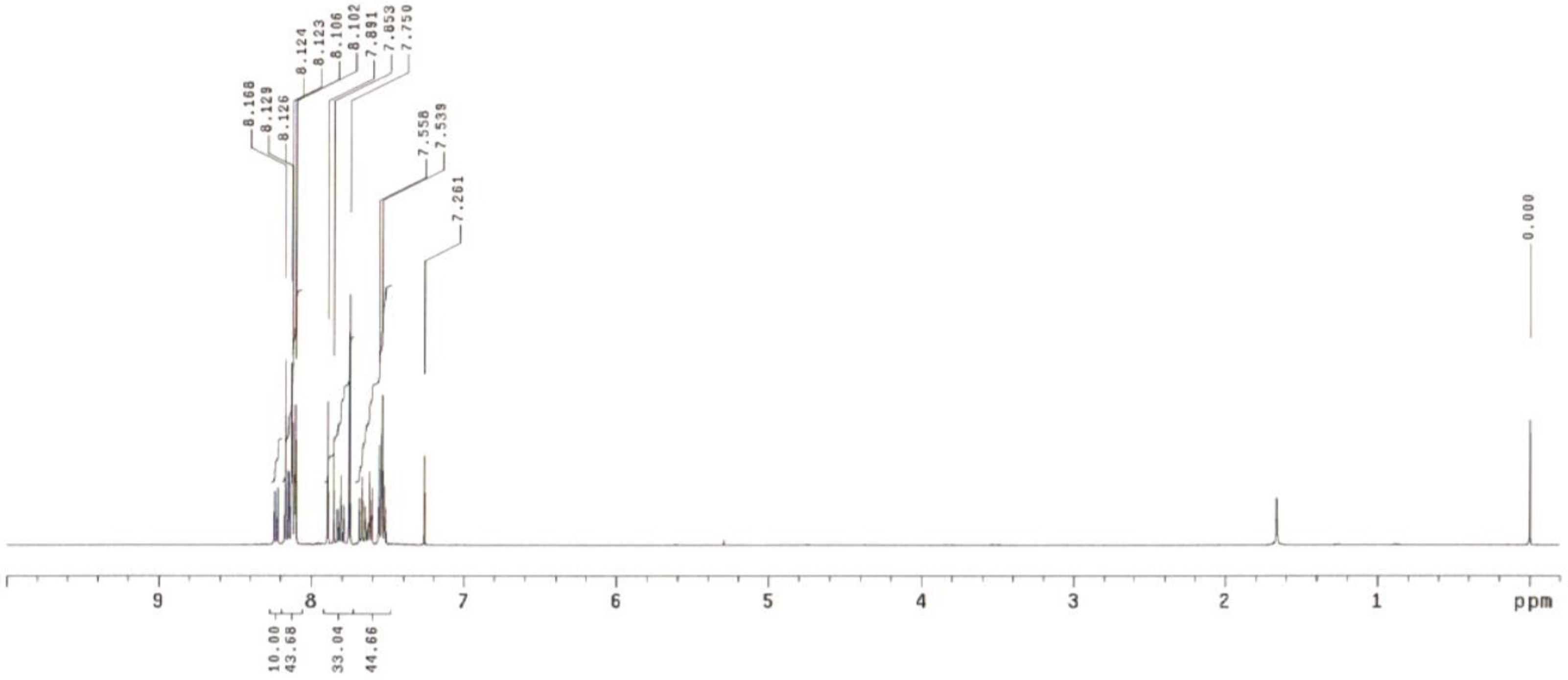

8.168
8.129
8.126
8.124
8.123
8.106
8.102
7.891
7.853
7.750
7.558
7.539
7.261
0.000
9
8
7
6
5
4
3
2
1
ppm
10.00
43.68
33.04
44.66

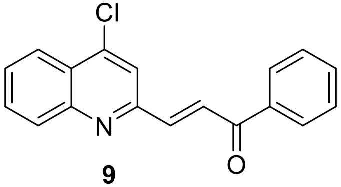
Cl
N
O

9

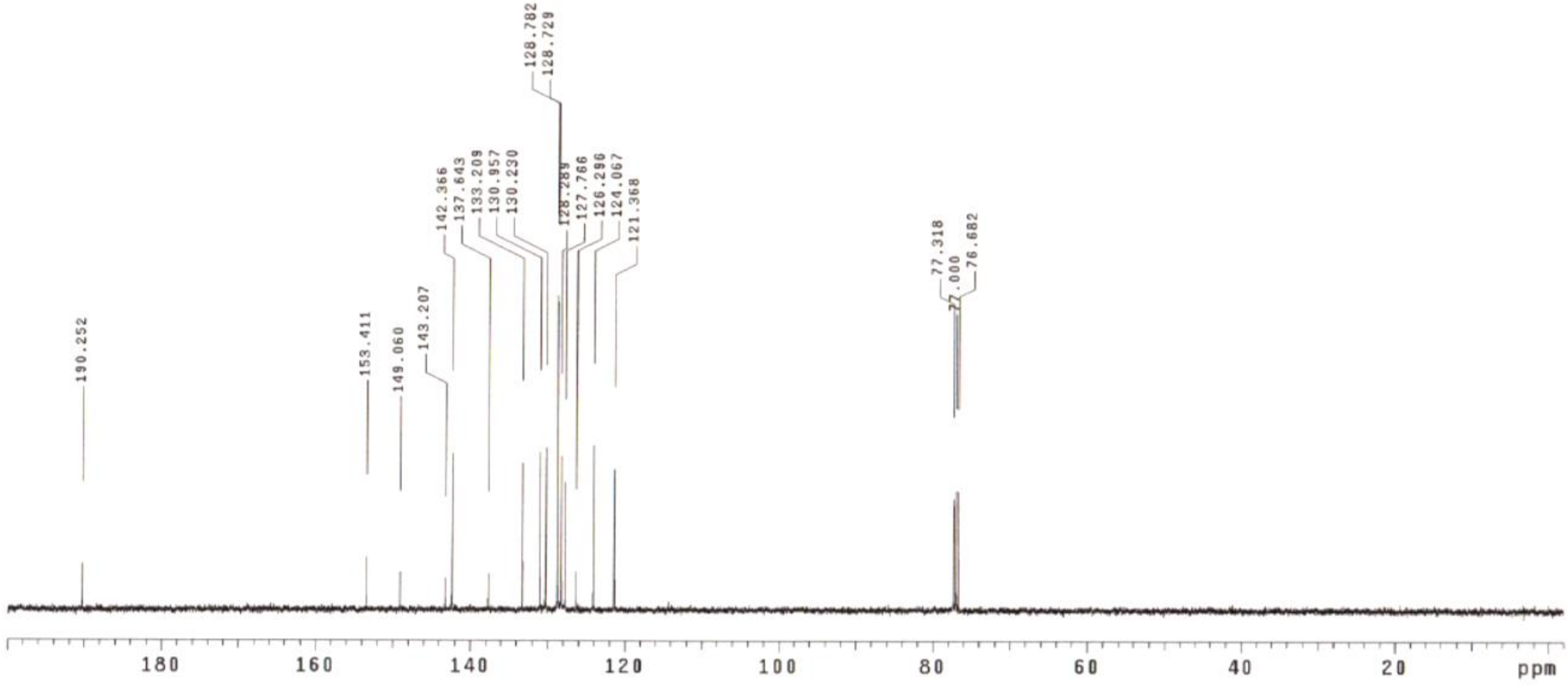
190.252
153.411
149.060
143.207
142.366
137.643
133.209
130.957
130.230
128.782
128.729
128.289
127.766
126.296
124.067
121.368
77.318
77.000
76.682
180
160
140
120
100
80
60
40
20
ppm

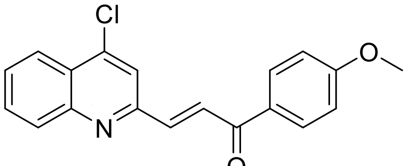
Cl
O
N
O

10

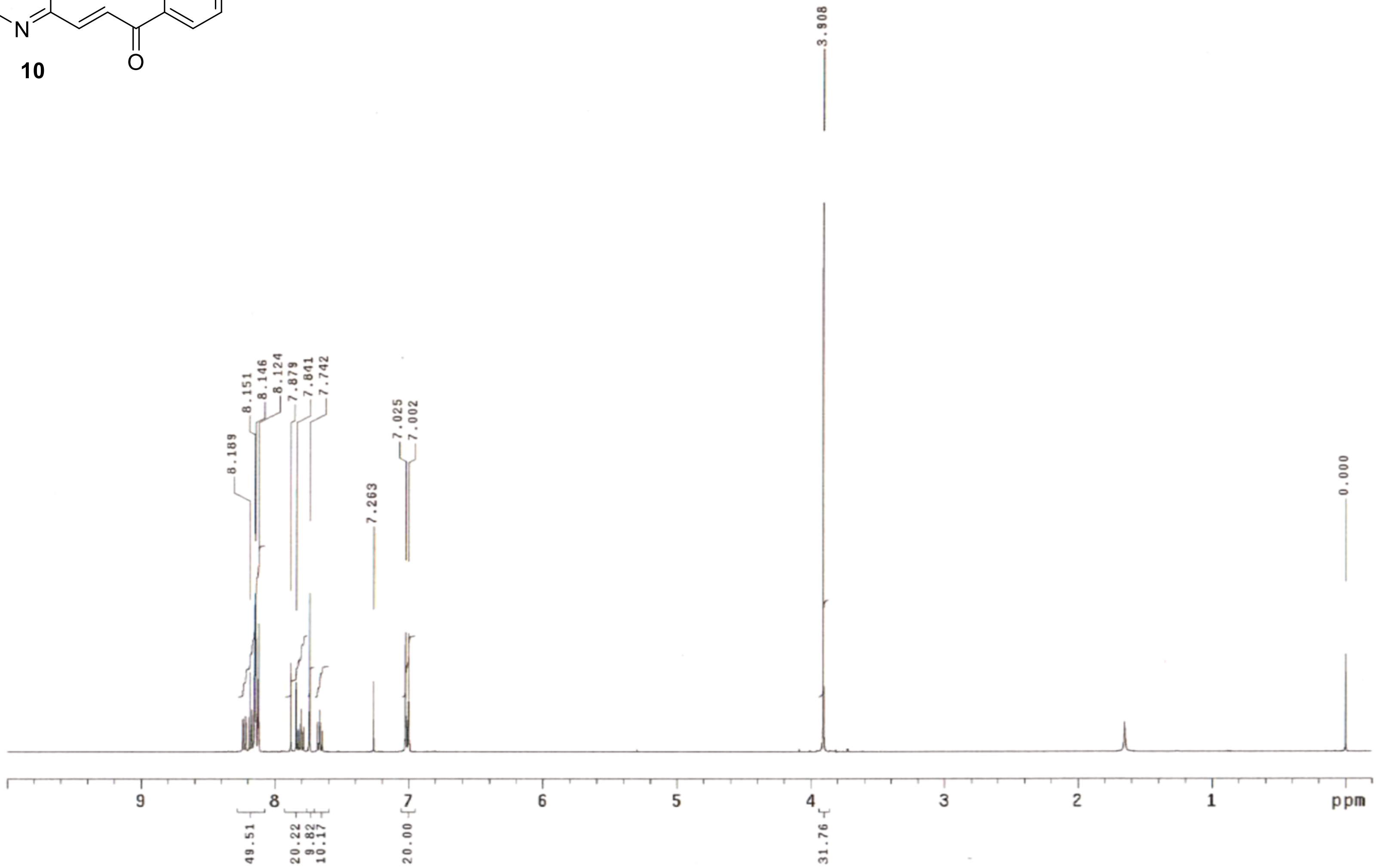
3.908
8.189
8.151
8.146
8.124
7.879
7.841
7.742
7.263
7.025
7.002
0.000
9
8
7
6
5
4
3
2
1
ppm
49.51
20.22
9.82
10.17
20.00
31.76

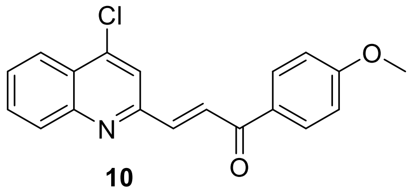

10

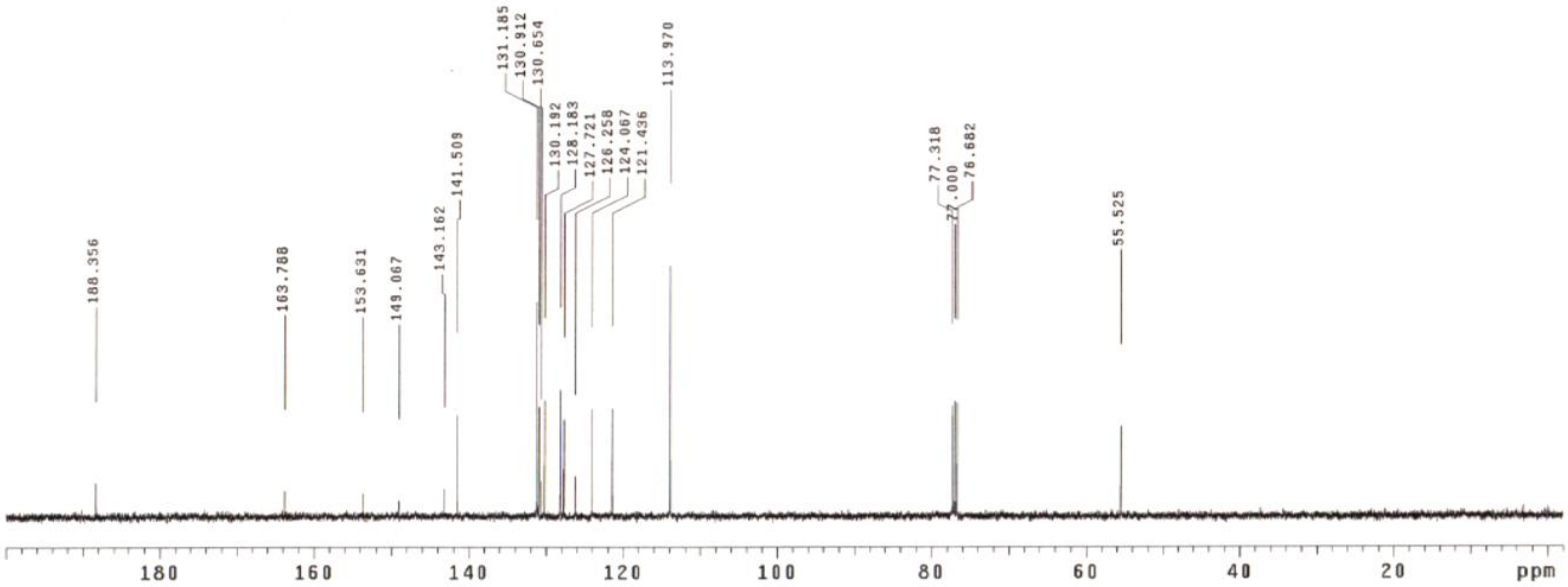

188.356
163.788
153.631
149.067
143.162
141.509
131.185
130.912
130.654
130.192
128.183
127.721
126.258
124.067
121.436
113.970
77.318
77.000
76.682
55.525
180
160
140
120
100
80
60
40
20
ppm

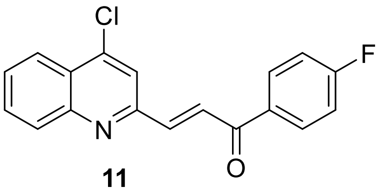
Cl
F
N
O

11

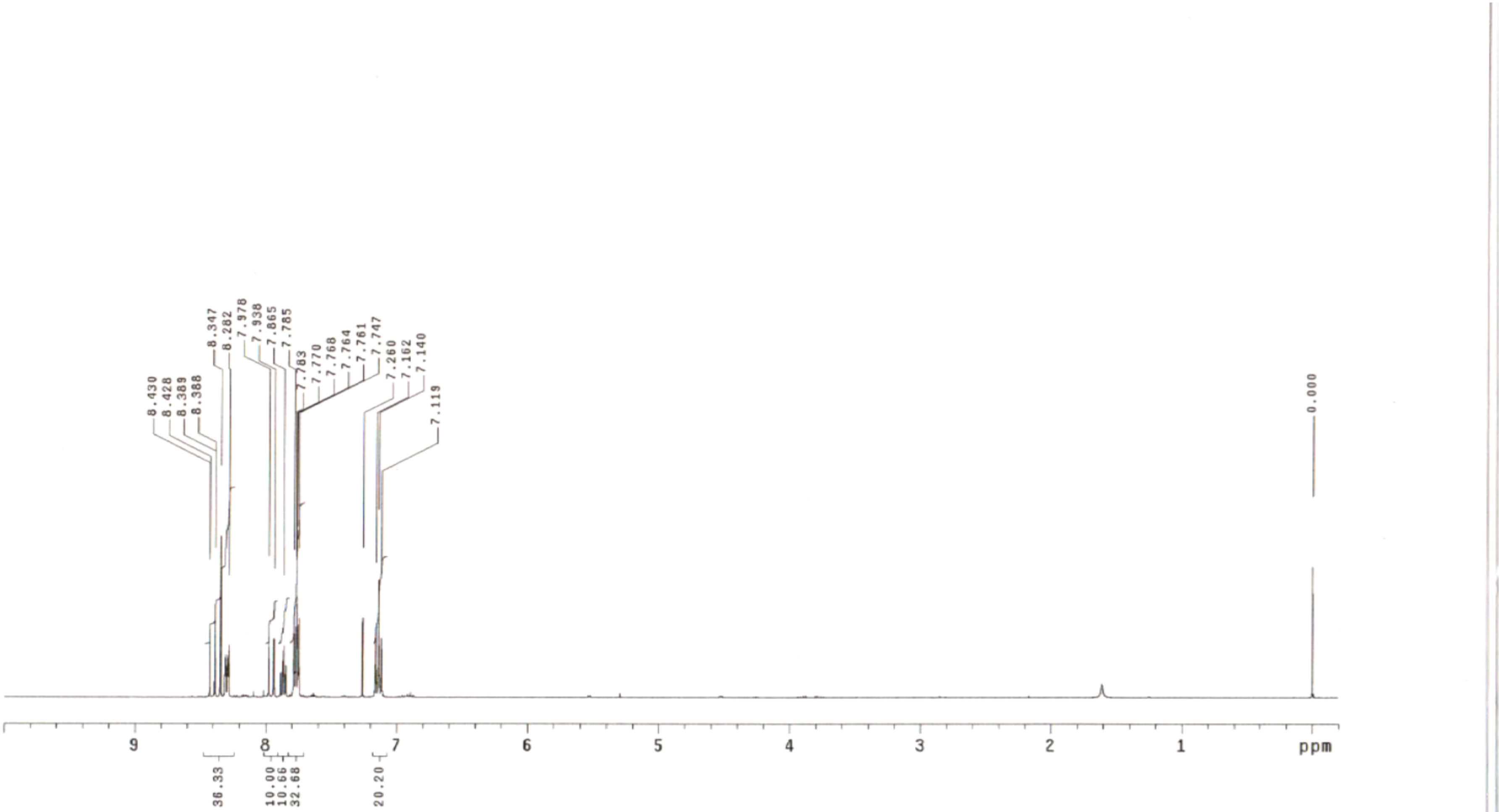
8.430
8.428
8.389
8.388
8.347
8.282
7.978
7.938
7.865
7.785
7.783
7.770
7.768
7.764
7.761
7.747
7.260
7.162
7.140
7.119
0.000
9
8
7
6
5
4
3
2
1
0
ppm
36.33
10.00
10.66
32.68
20.20

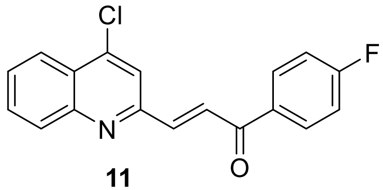
Cl
F
N
O

11

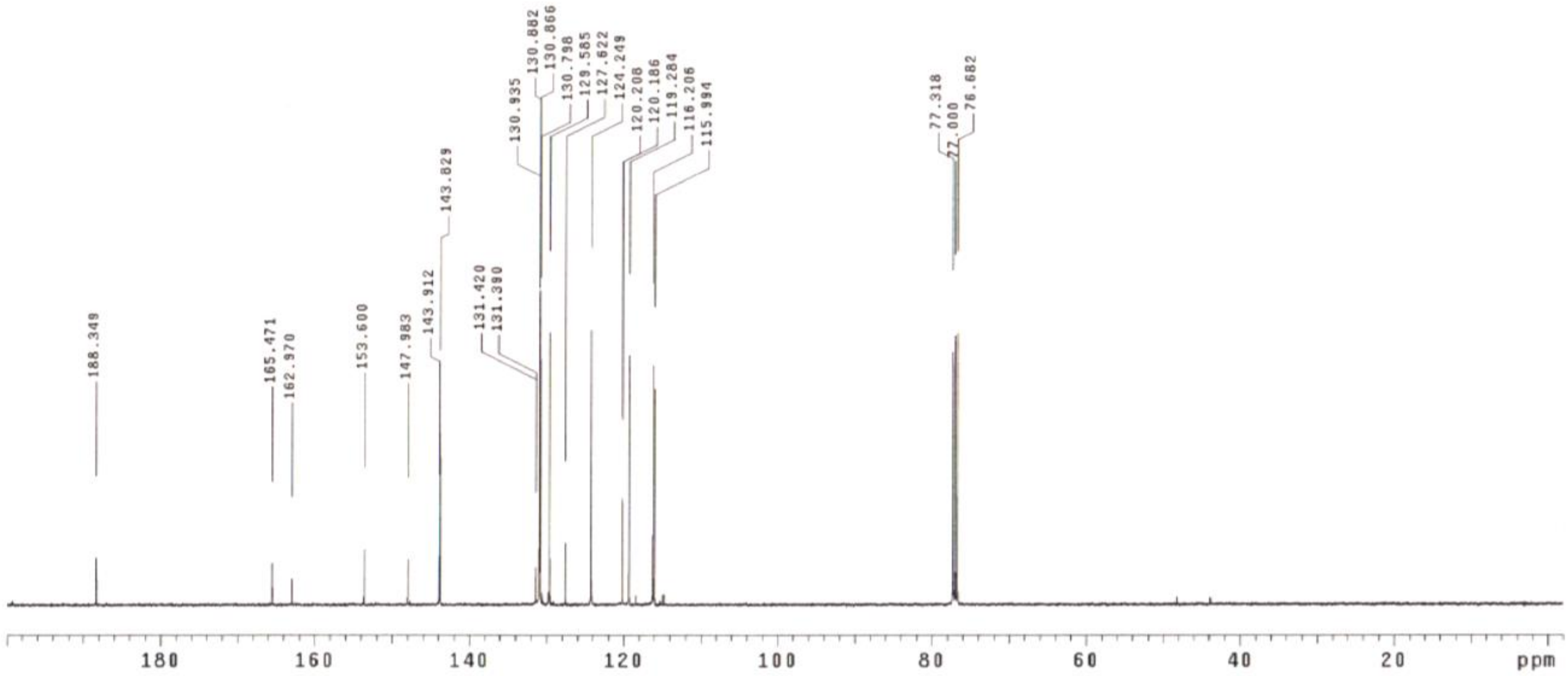
188.349
165.471
162.970
153.600
147.983
143.912
143.829
131.420
131.390
130.935
130.882
130.866
130.798
129.585
127.622
124.249
120.208
120.186
119.284
116.206
115.994
77.318
77.000
76.682
180
160
140
120
100
80
60
40
20
ppm

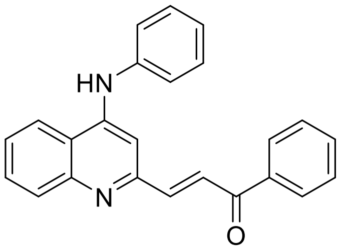
HN
N
O

12a

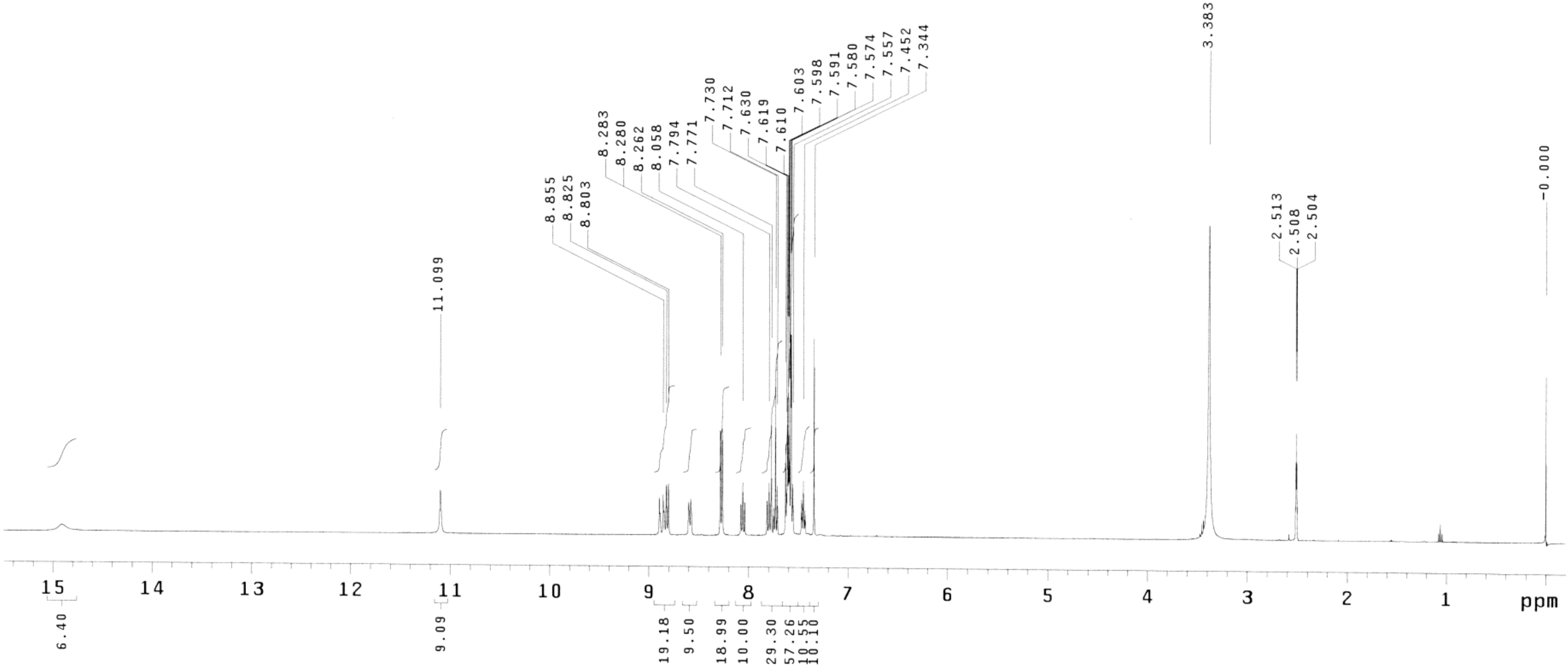
11.099
8.855
8.825
8.803
8.283
8.280
8.262
8.058
7.794
7.771
7.730
7.712
7.630
7.619
7.610
7.603
7.598
7.591
7.580
7.574
7.557
7.452
7.344
3.383
2.513
2.508
2.504
-0.000
15 14 13 12 11 10 9 8 7 6 5 4 3 2 1 ppm
6.40
9.09
19.18
9.50
18.99
10.00
29.30
57.26
10.55
10.10

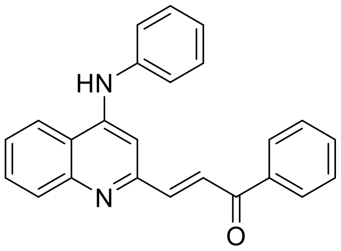
HN
N
O

12a

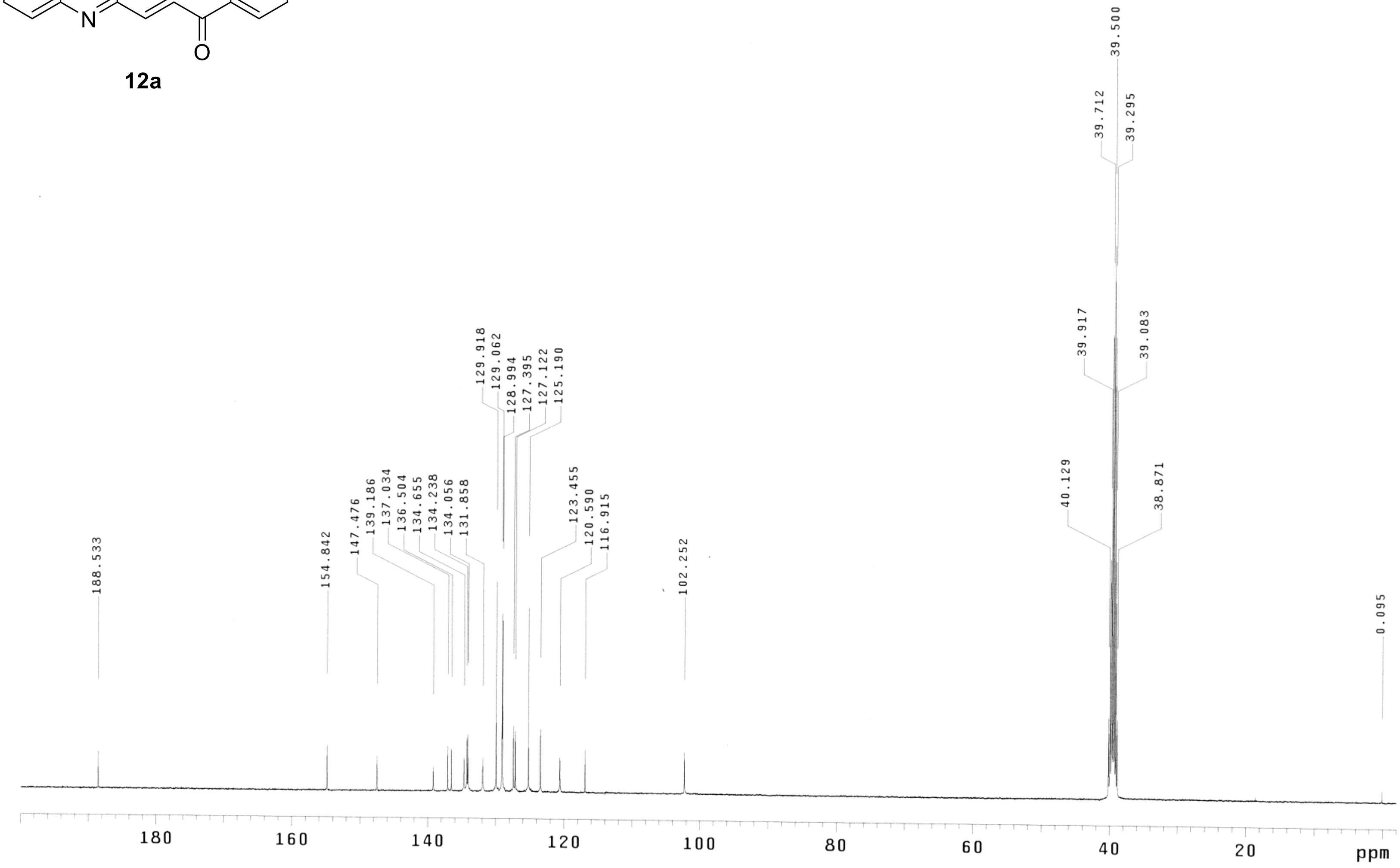
188.533
154.842
147.476
139.186
137.034
136.504
134.655
134.238
134.056
131.858
129.918
129.062
128.994
127.395
127.122
125.190
123.455
120.590
116.915
102.252
40.129
39.917
39.712
39.500
39.295
39.083
38.871
0.095
180
160
140
120
100
80
60
40
20
ppm

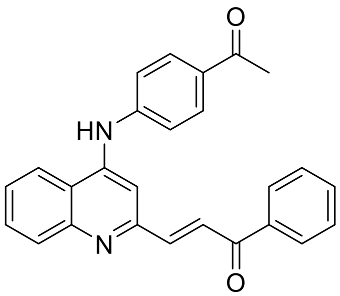

12b

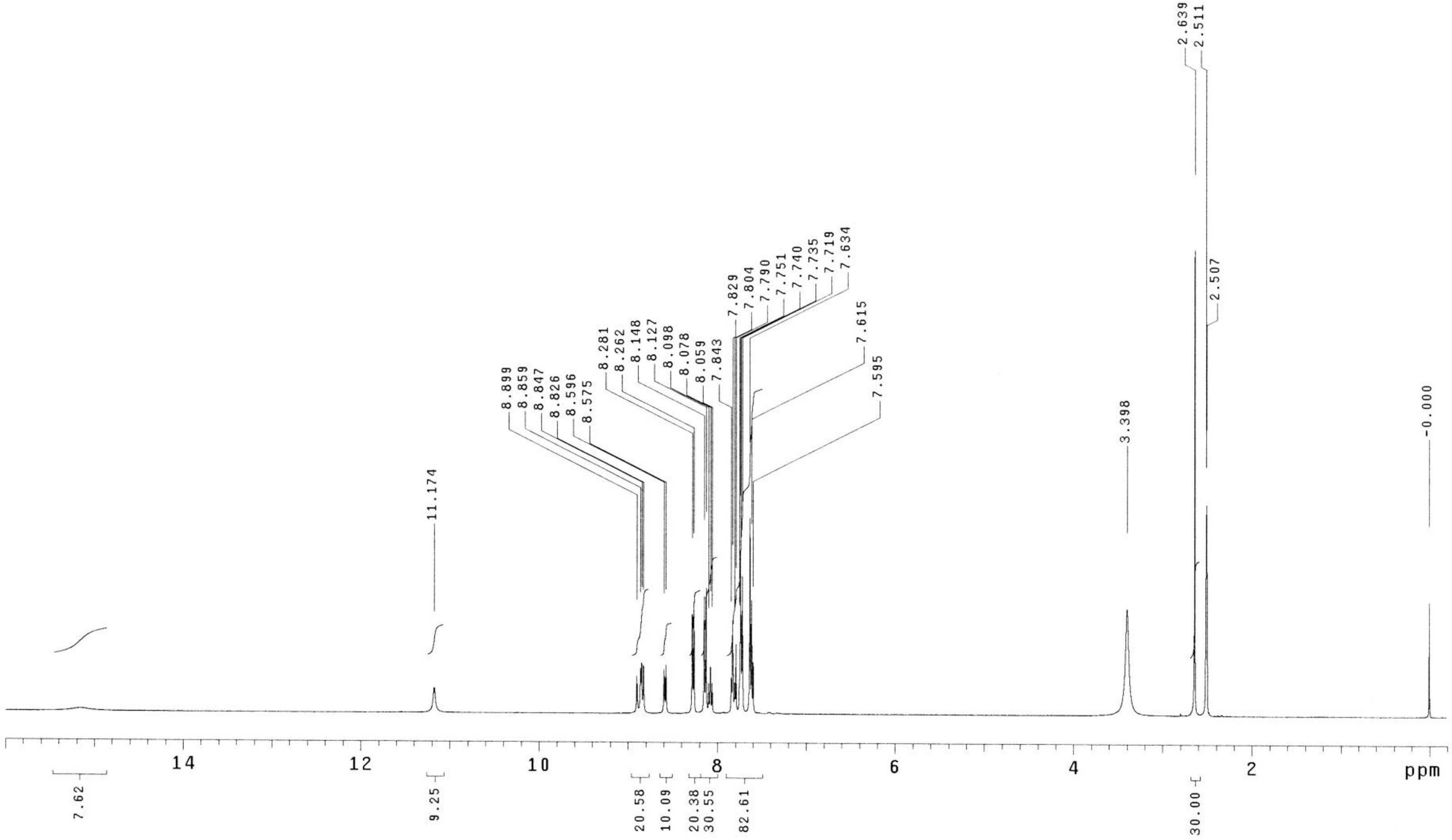

11.174
8.899
8.859
8.847
8.826
8.596
8.575
8.281
8.262
8.148
8.127
8.098
8.078
8.059
7.843
7.829
7.804
7.790
7.751
7.740
7.735
7.719
7.634
7.615
7.595
3.398
2.639
2.511
2.507
-0.000
14
12
10
8
6
4
2
ppm
7.62
9.25
20.58
10.09
20.38
30.55
82.61
30.00

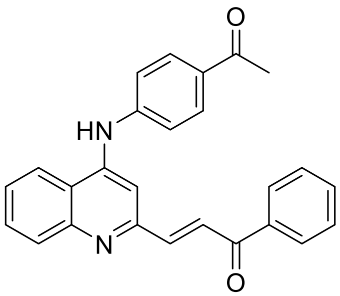
HN
N
O
O

12b

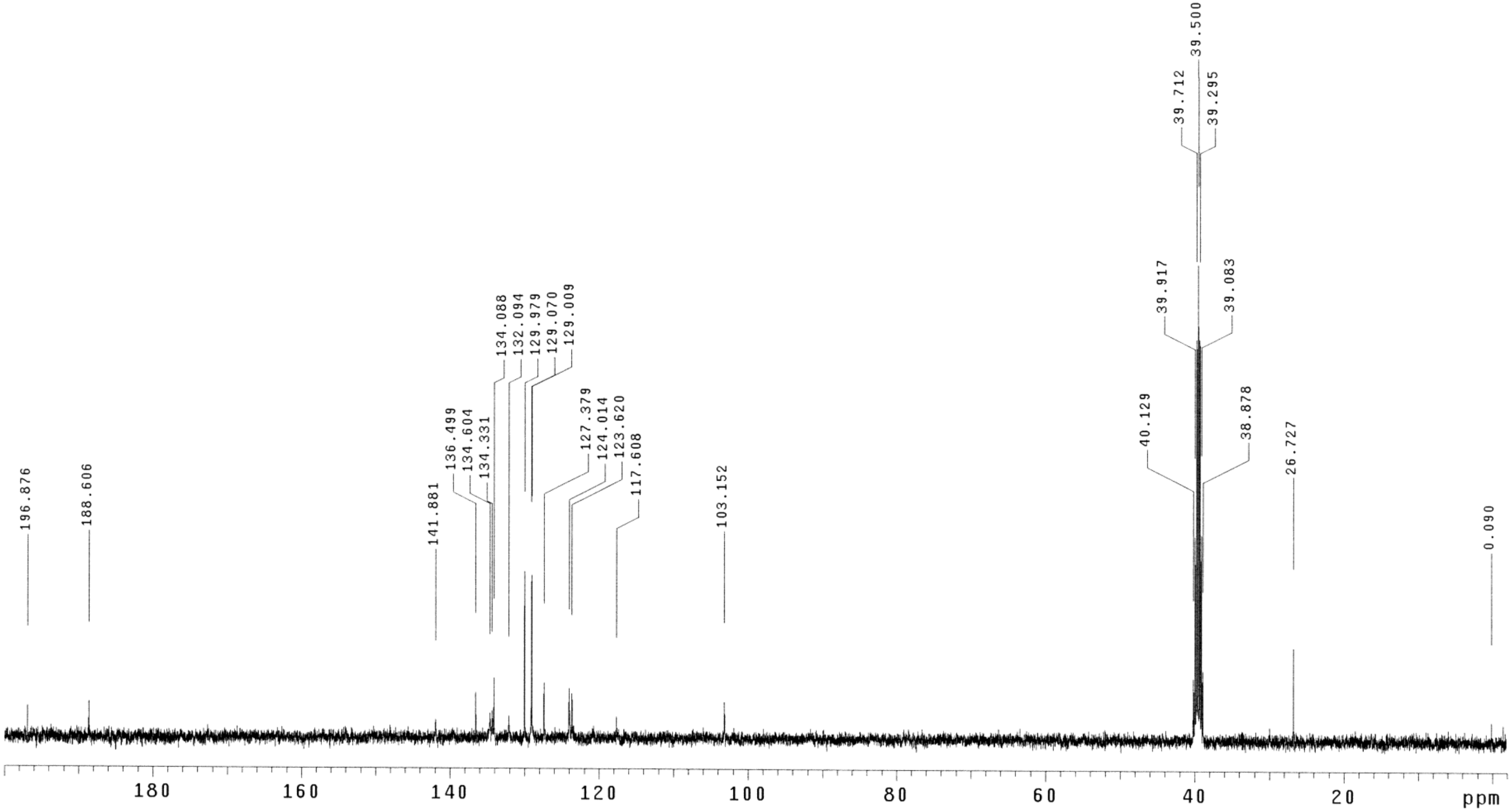
196.876
188.606
141.881
136.499
134.604
134.331
134.088
132.094
129.979
129.070
129.009
127.379
124.014
123.620
117.608
103.152
40.129
39.917
39.712
39.500
39.295
39.083
38.878
26.727
0.090
180
160
140
120
100
80
60
40
20
ppm

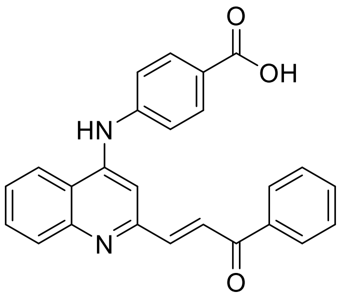

O
OH
HN
N
O

12c

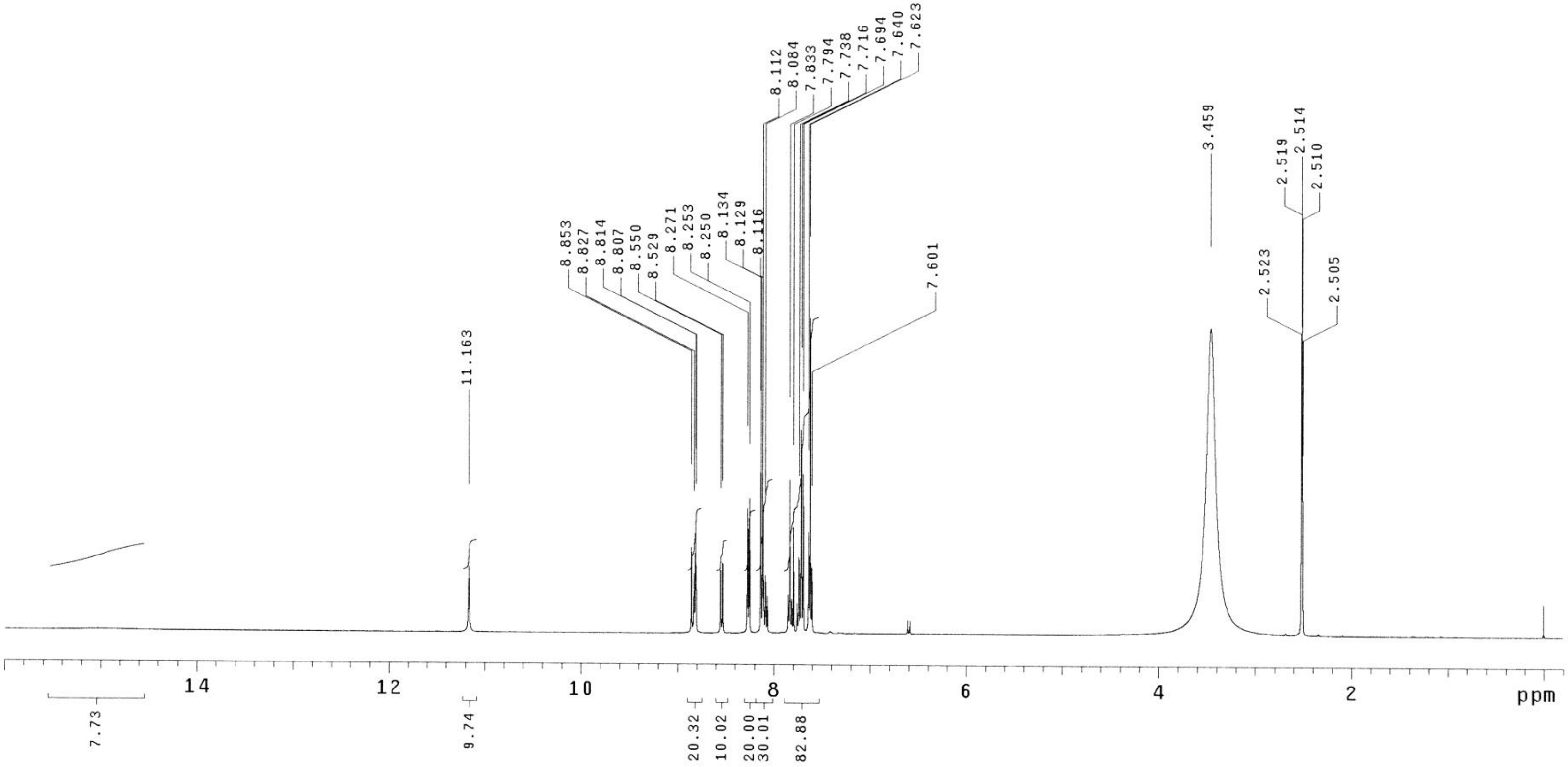

11.163
8.853
8.827
8.814
8.807
8.550
8.529
8.271
8.253
8.250
8.134
8.129
8.116
8.112
8.084
7.833
7.794
7.738
7.716
7.694
7.640
7.623
7.601
3.459
2.523
2.519
2.514
2.510
2.505
14
12
10
8
6
4
2
ppm
7.73
9.74
20.32
10.02
20.00
30.01
82.88

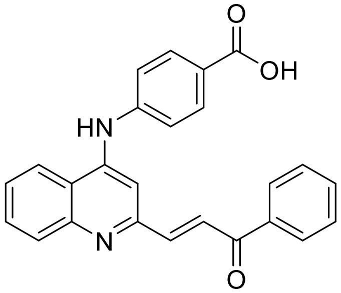

12c

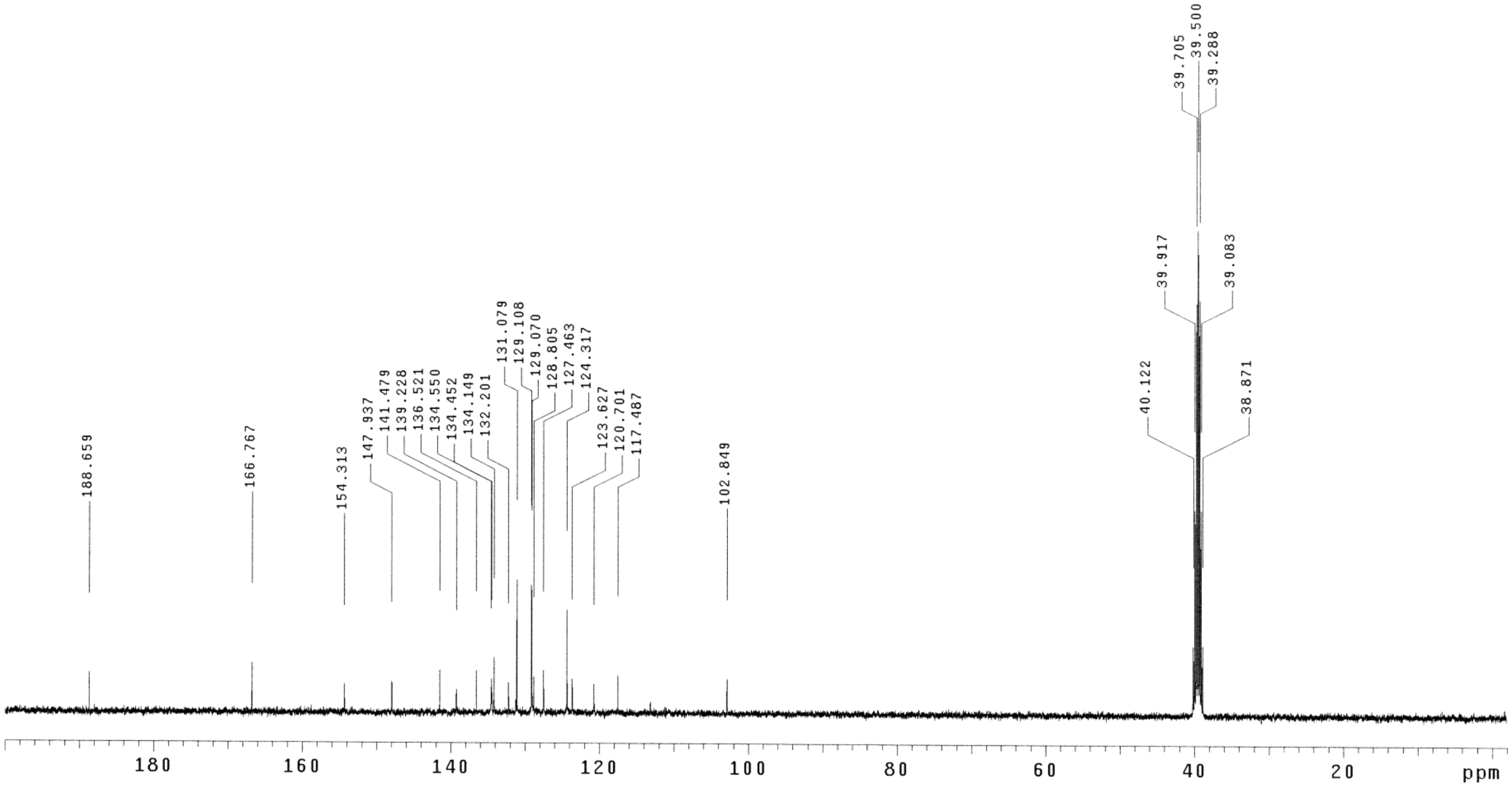
188.659
166.767
154.313
147.937
141.479
139.228
136.521
134.550
134.452
134.149
132.201
131.079
129.108
129.070
128.805
127.463
124.317
123.627
120.701
117.487
102.849
40.122
39.917
39.705
39.500
39.288
39.083
38.871
180
160
140
120
100
80
60
40
20
ppm

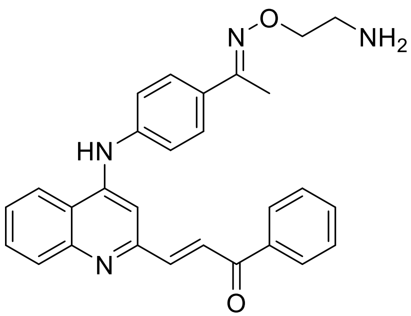
N
O
NH2
HN
N
O

12d

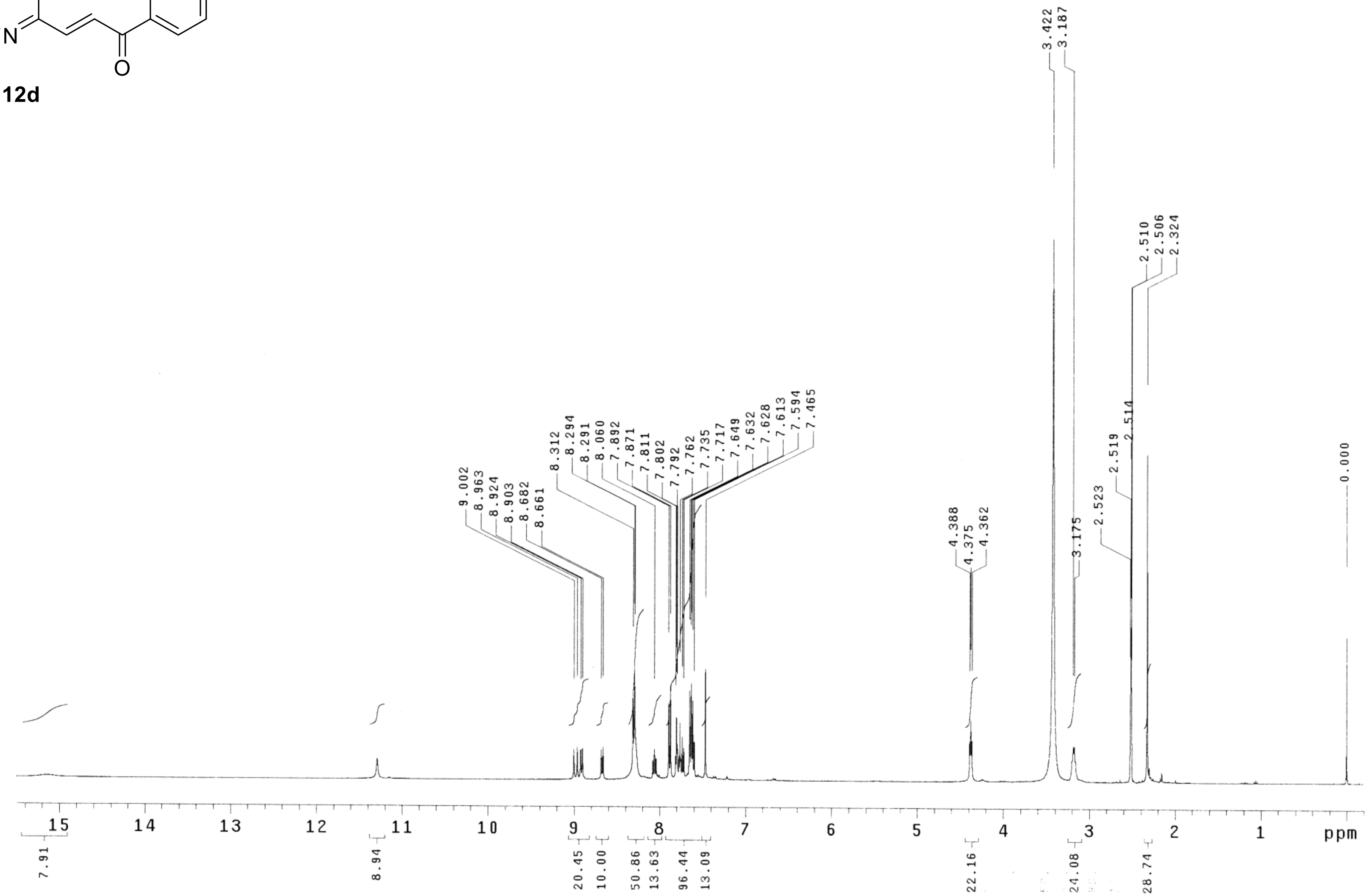
3.422
3.187
2.510
2.506
2.324
9.002
8.963
8.924
8.903
8.682
8.661
8.312
8.294
8.291
8.060
7.892
7.871
7.811
7.802
7.792
7.762
7.735
7.717
7.649
7.632
7.628
7.613
7.594
7.465
4.388
4.375
4.362
3.175
2.523
2.519
2.514
0.000
15
14
13
12
11
10
9
8
7
6
5
4
3
2
1
ppm
7.91
8.94
20.45
10.00
50.86
13.63
96.44
13.09
22.16
24.08
28.74

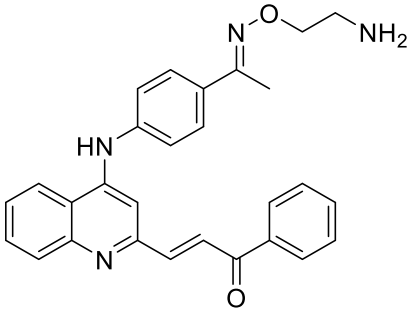
N
O
NH2
HN
N
O

12d

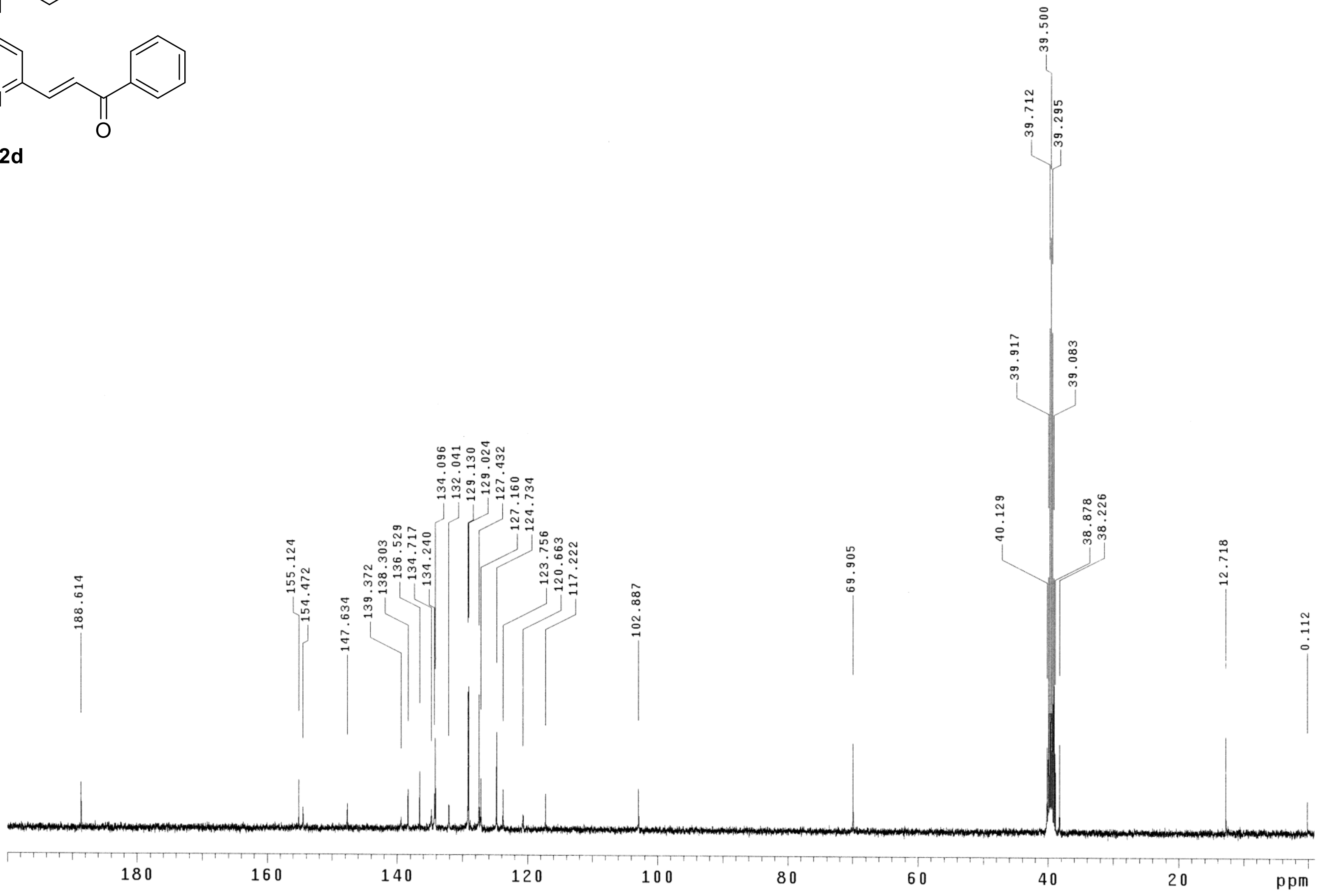
188.614
155.124
154.472
147.634
139.372
138.303
136.529
134.717
134.240
134.096
132.041
129.130
129.024
127.432
127.160
124.734
123.756
120.663
117.222
102.887
69.905
40.129
39.917
39.712
39.500
39.295
39.083
38.878
38.226
12.718
0.112
180
160
140
120
100
80
60
40
20
ppm

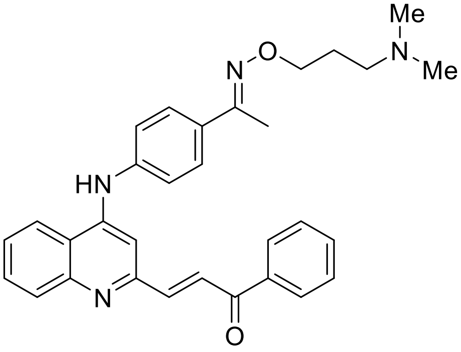

12e

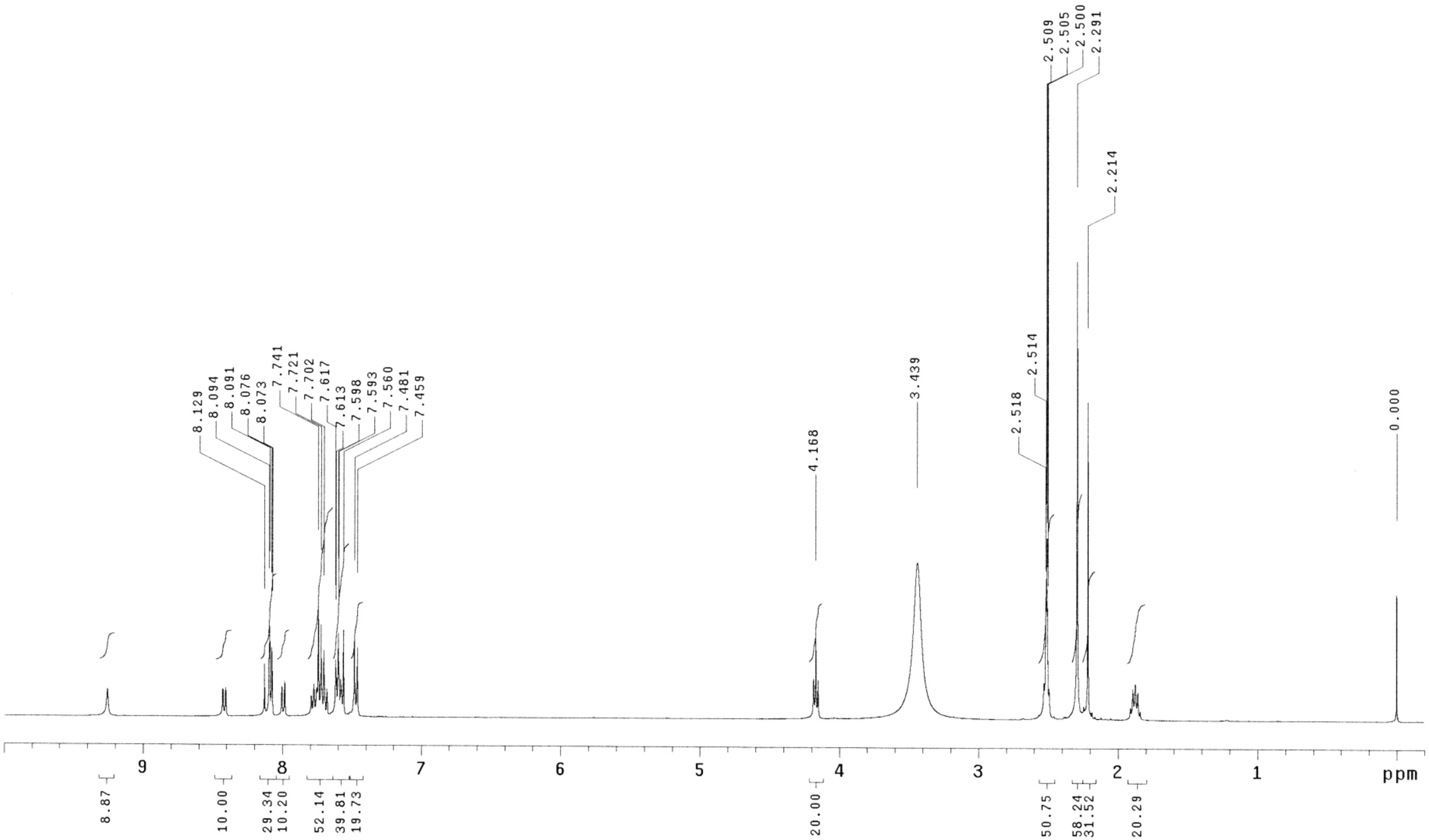

8.129
8.094
8.091
8.076
8.073
7.741
7.721
7.702
7.617
7.613
7.598
7.593
7.560
7.481
7.459
4.168
3.439
2.518
2.514
2.509
2.505
2.500
2.291
2.214
0.000
9
8
7
6
5
4
3
2
1
ppm
8.87
10.00
29.34
10.20
52.14
39.81
19.73
20.00
50.75
58.24
31.52
20.29

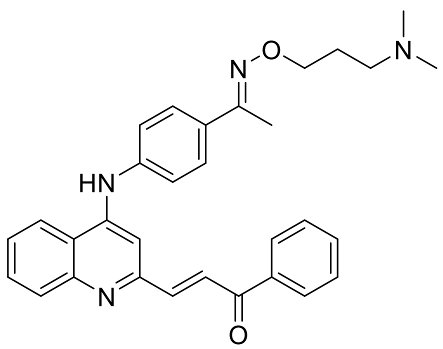

12e

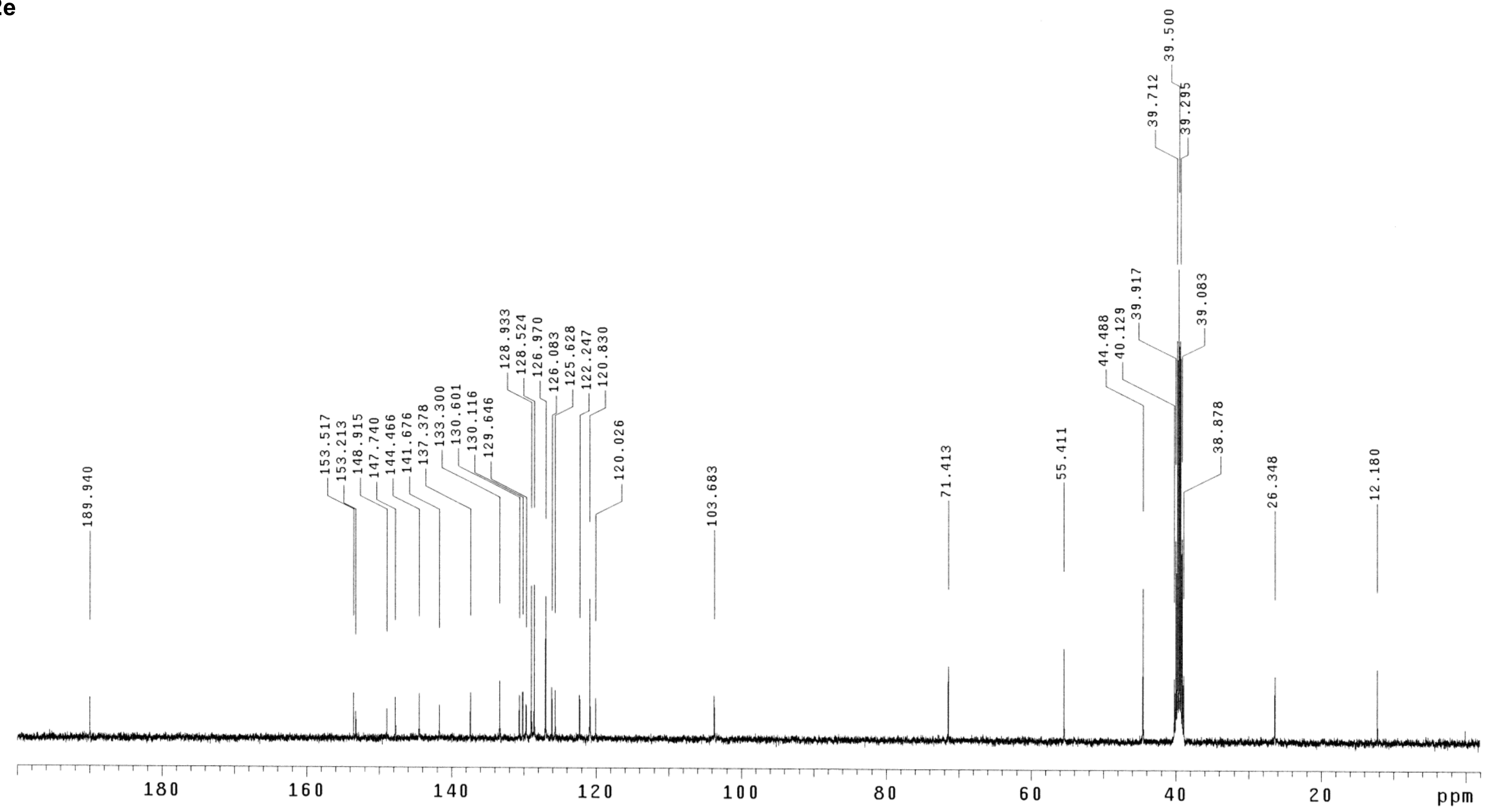
189.940
153.517
153.213
148.915
147.740
144.466
141.676
137.378
133.300
130.601
130.116
129.646
128.933
128.524
126.970
126.083
125.628
122.247
120.830
120.026
103.683
71.413
55.411
44.488
40.129
39.917
39.712
39.500
39.295
39.083
38.878
26.348
12.180
180
160
140
120
100
80
60
40
20
ppm

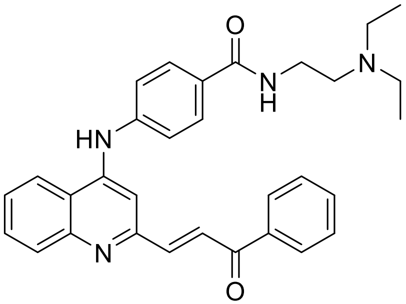
O
N
H
N
HN
N
O

12f

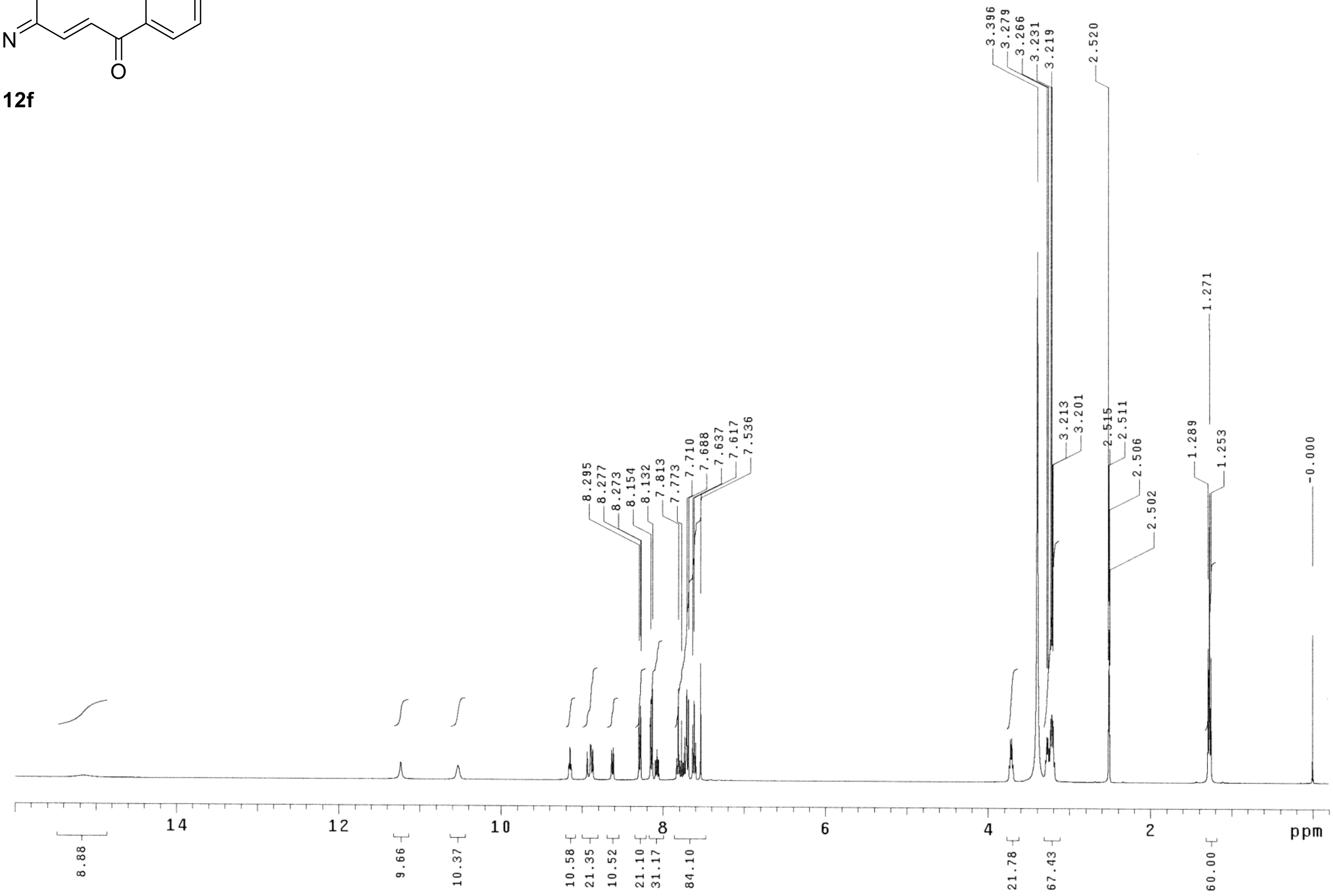
3.396
3.279
3.266
3.231
3.219
2.520
1.271
3.213
3.201
2.515
2.511
2.506
2.502
1.289
1.253
-0.000
8.295
8.277
8.273
8.154
8.132
7.813
7.773
7.710
7.688
7.637
7.617
7.536
14
12
10
8
6
4
2
ppm
8.88
9.66
10.37
10.58
21.35
10.52
21.10
31.17
84.10
21.78
67.43
60.00

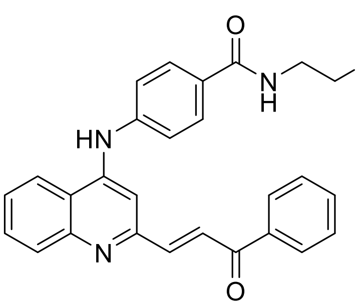
O
N
H
N
HN
N
O

12f

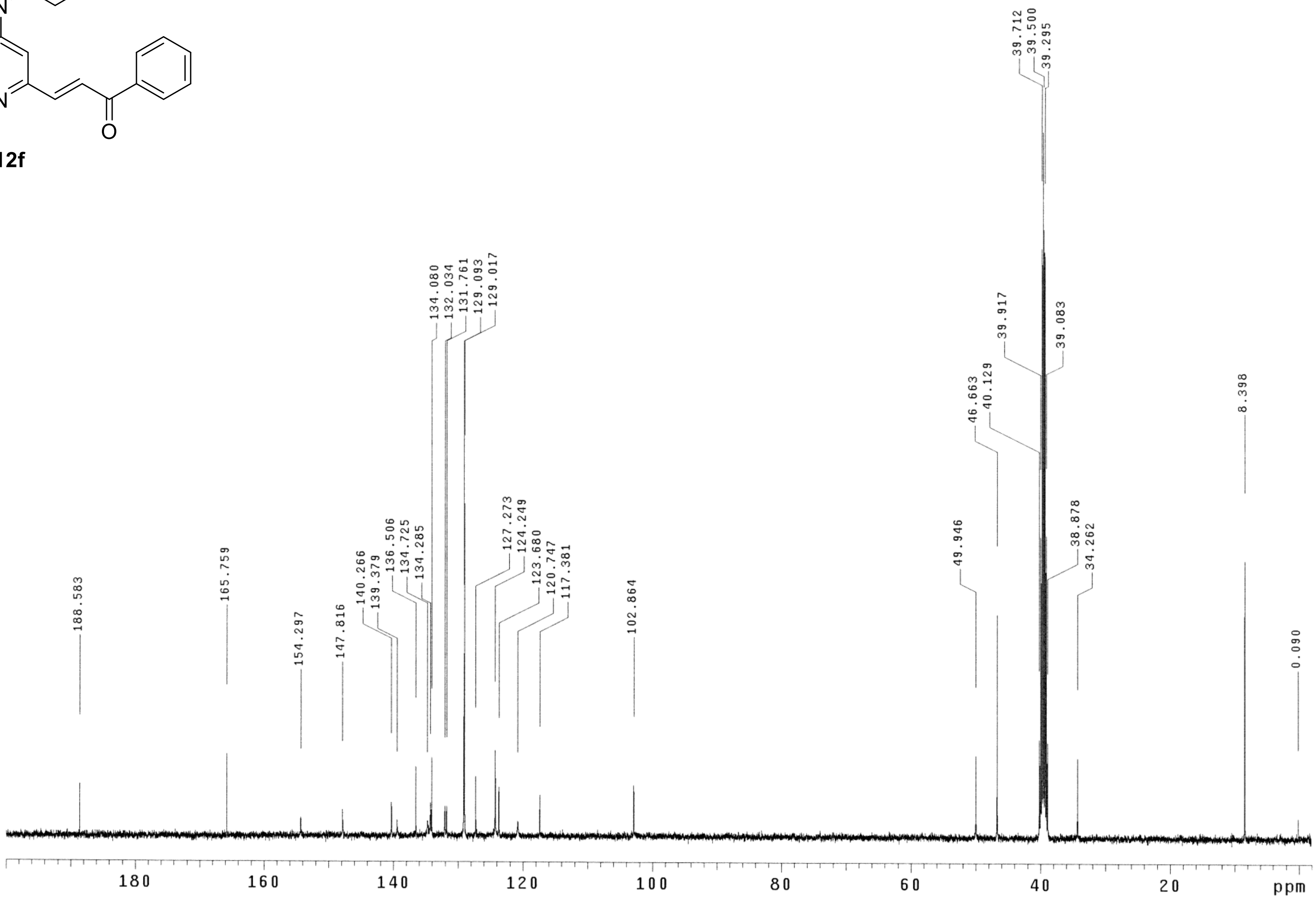
188.583
165.759
154.297
147.816
140.266
139.379
136.506
134.725
134.285
134.080
132.034
131.761
129.093
129.017
127.273
124.249
123.680
120.747
117.381
102.864
49.946
46.663
40.129
39.917
39.712
39.500
39.295
39.083
38.878
34.262
8.398
0.090
180
160
140
120
100
80
60
40
20
ppm

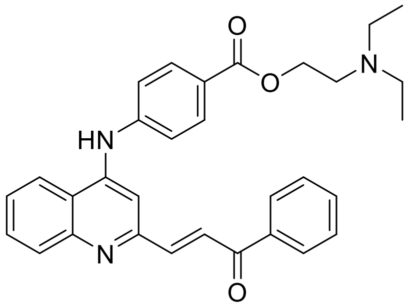

12g

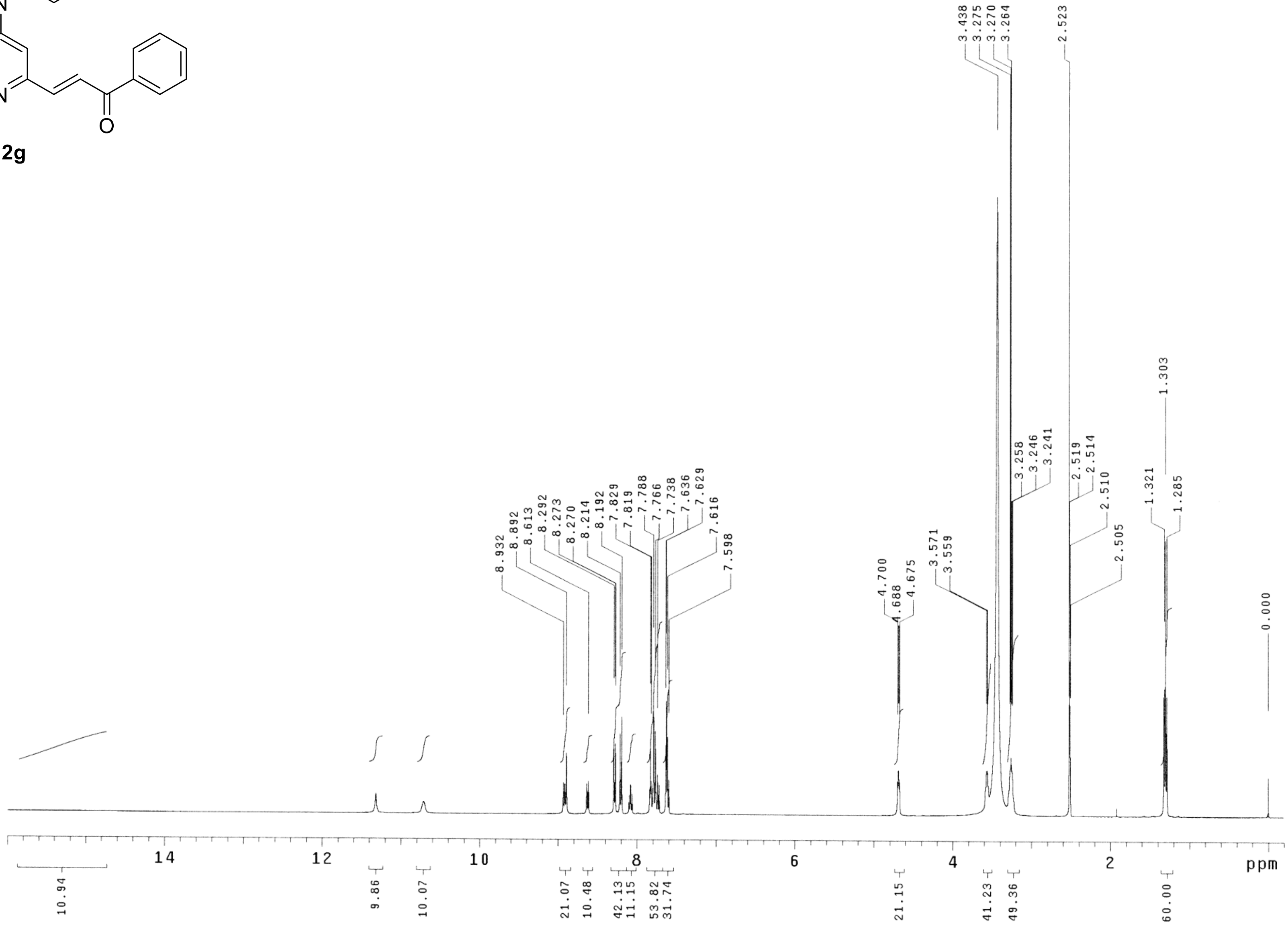
3.438
3.275
3.270
3.264
2.523
1.303
3.258
3.246
3.241
2.519
2.514
2.510
2.505
1.321
1.285
8.932
8.892
8.613
8.292
8.273
8.270
8.214
8.192
7.829
7.819
7.788
7.766
7.738
7.636
7.629
7.616
7.598
4.700
4.688
4.675
3.571
3.559
0.000
14
12
10
8
6
4
2
ppm
10.94
9.86
10.07
21.07
10.48
42.13
11.15
53.82
31.74
21.15
41.23
49.36
60.00

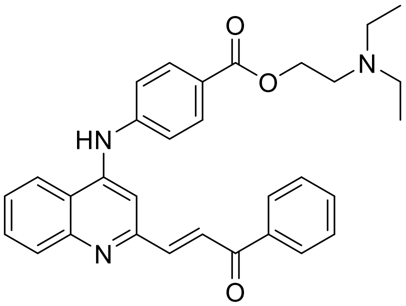

12g

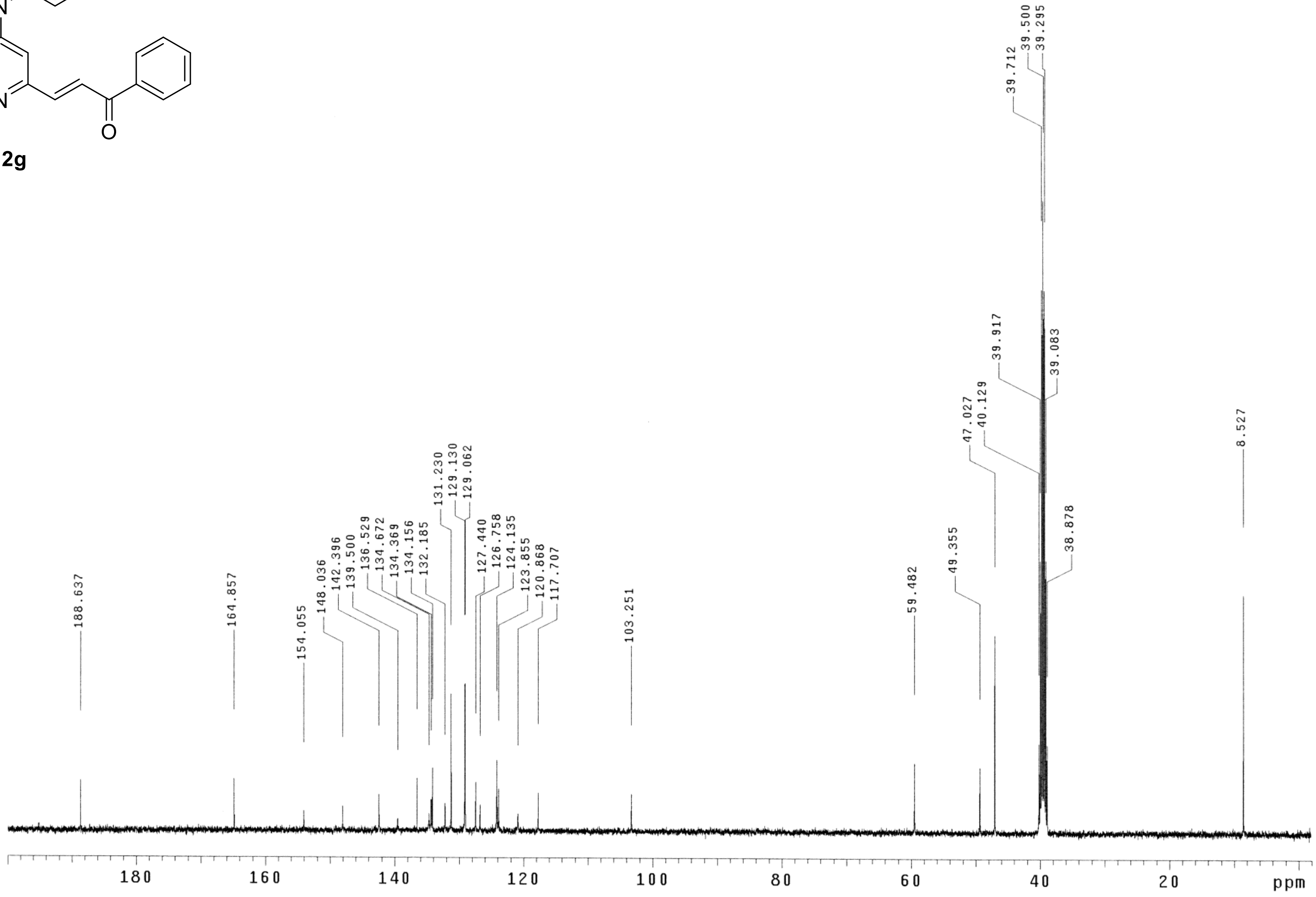
188.637
164.857
154.055
148.036
142.396
139.500
136.529
134.672
134.369
134.156
132.185
131.230
129.130
129.062
127.440
126.758
124.135
123.855
120.868
117.707
103.251
59.482
49.355
47.027
40.129
39.917
39.712
39.500
39.295
39.083
38.878
8.527
180
160
140
120
100
80
60
40
20
ppm

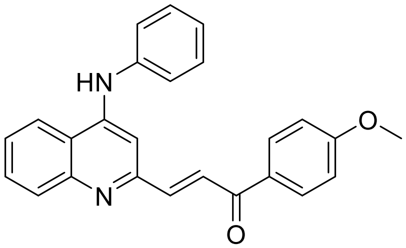
HN
N
O
O

13a

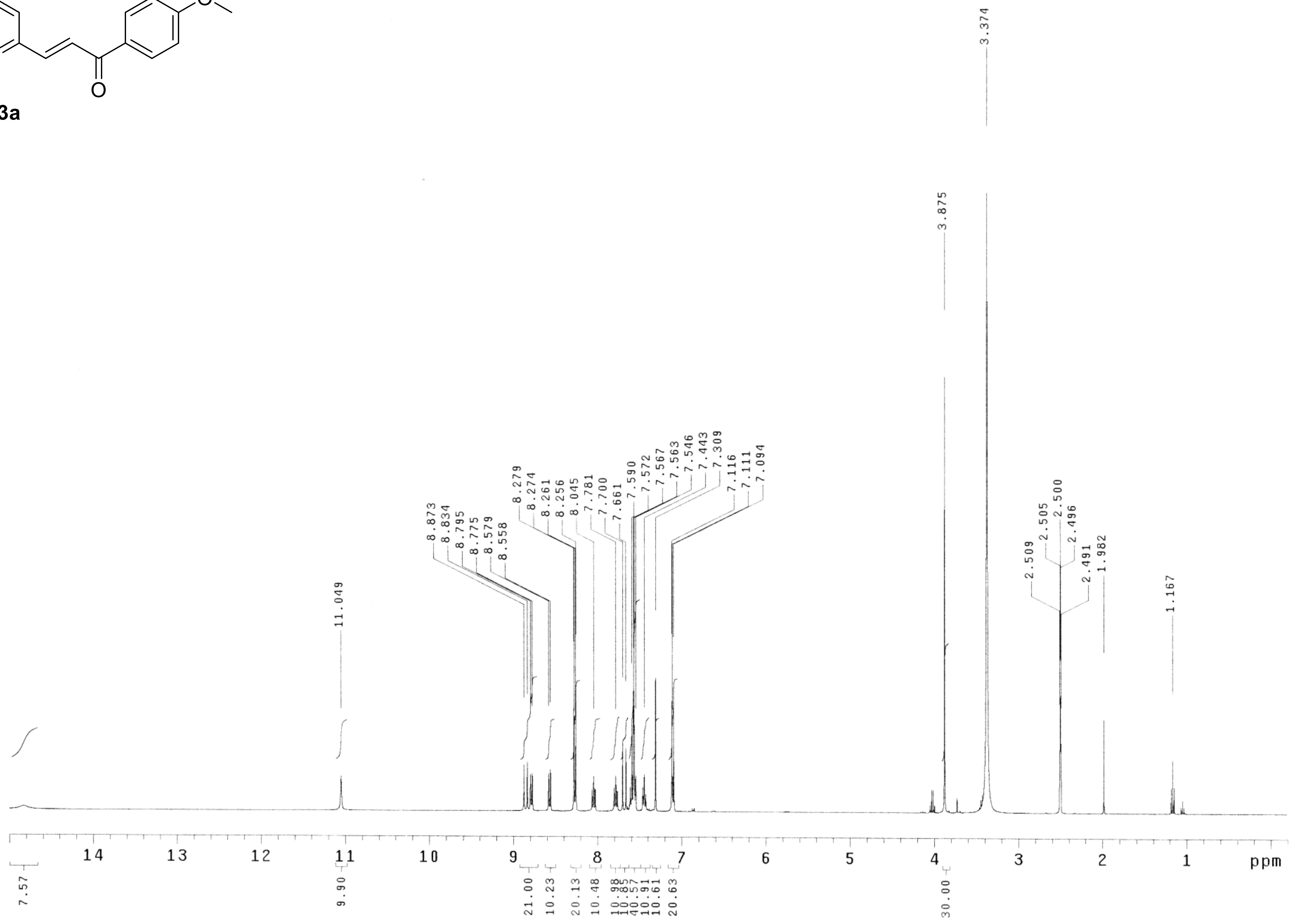
11.049
8.873
8.834
8.795
8.775
8.579
8.558
8.279
8.274
8.261
8.256
8.045
7.781
7.700
7.661
7.590
7.572
7.567
7.563
7.546
7.443
7.309
7.116
7.111
7.094
3.875
3.374
2.509
2.505
2.500
2.496
2.491
1.982
1.167
14 13 12 11 10 9 8 7 6 5 4 3 2 1 ppm
7.57
9.90
21.00
10.23
20.13
10.48
10.98
10.85
40.57
10.91
10.61
20.63
30.00

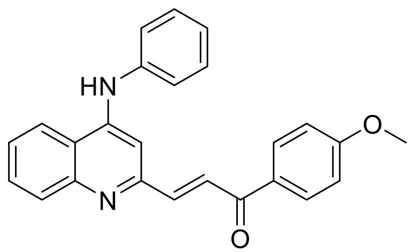

13a

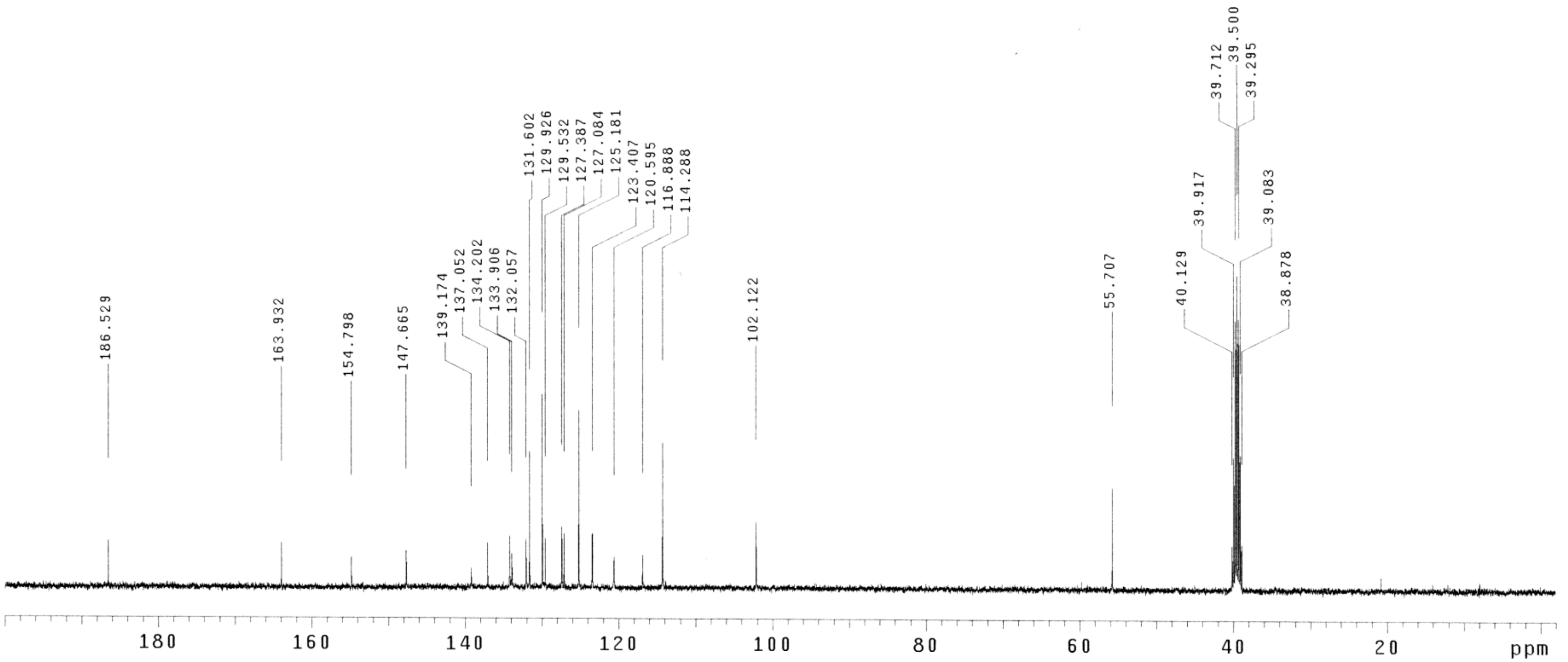

186.529
163.932
154.798
147.665
139.174
137.052
134.202
133.906
132.057
131.602
129.926
129.532
127.387
127.084
125.181
123.407
120.595
116.888
114.288
102.122
55.707
40.129
39.917
39.712
39.500
39.295
39.083
38.878
180
160
140
120
100
80
60
40
20
ppm

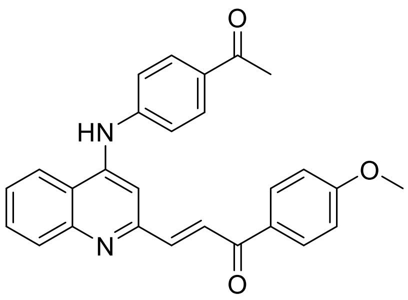

13b

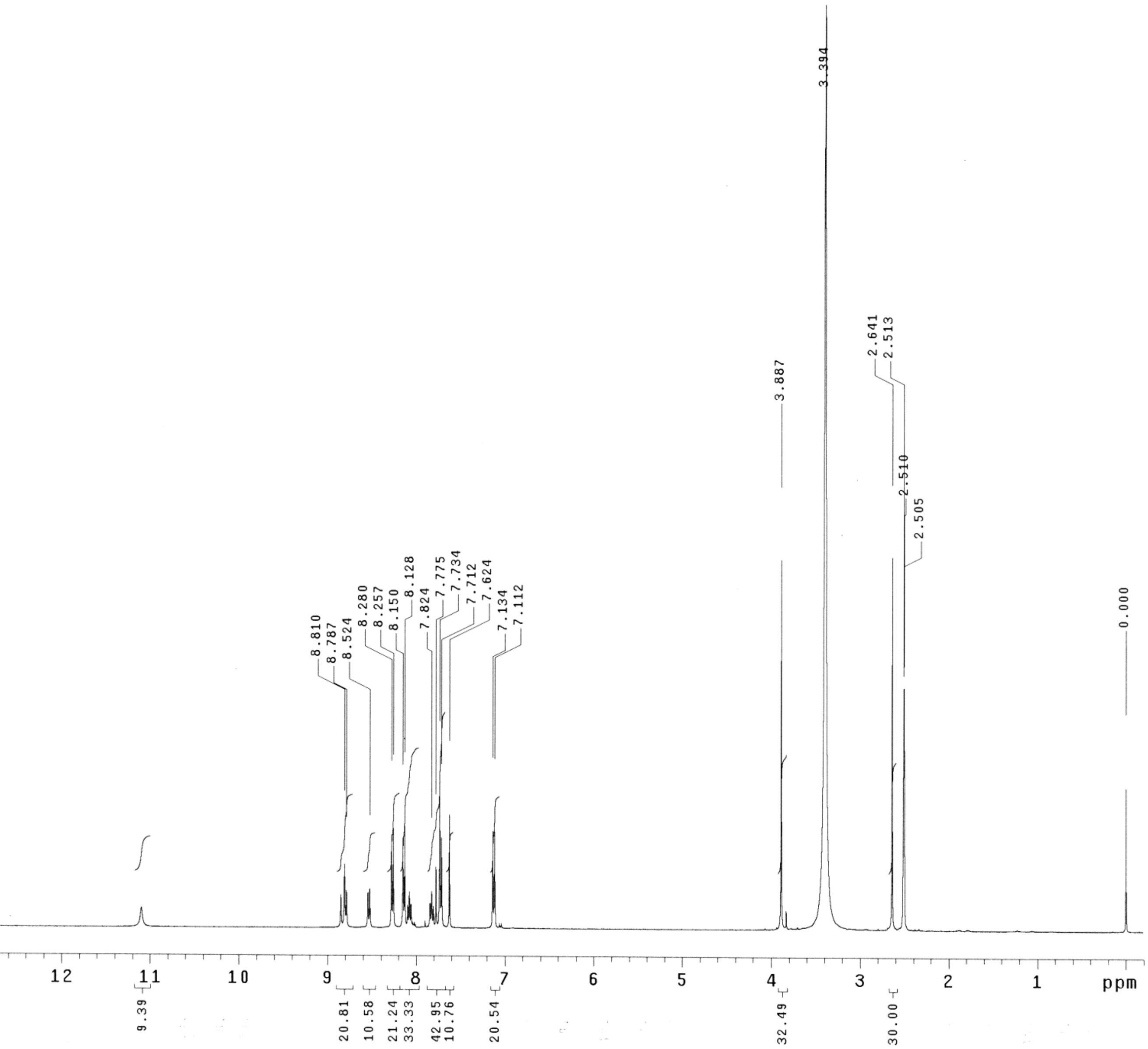

8.810
8.787
8.524
8.280
8.257
8.150
8.128
7.824
7.775
7.734
7.712
7.624
7.134
7.112
3.887
3.394
2.641
2.513
2.510
2.505
0.000
15
14
13
12
11
10
9
8
7
6
5
4
3
2
1
ppm
8.52
9.39
20.81
10.58
21.24
33.33
42.95
10.76
20.54
32.49
30.00

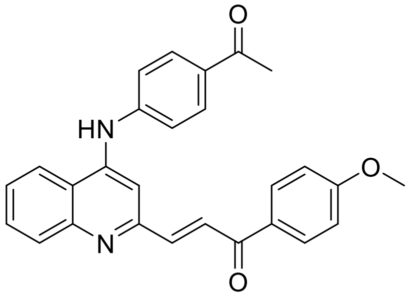

13b

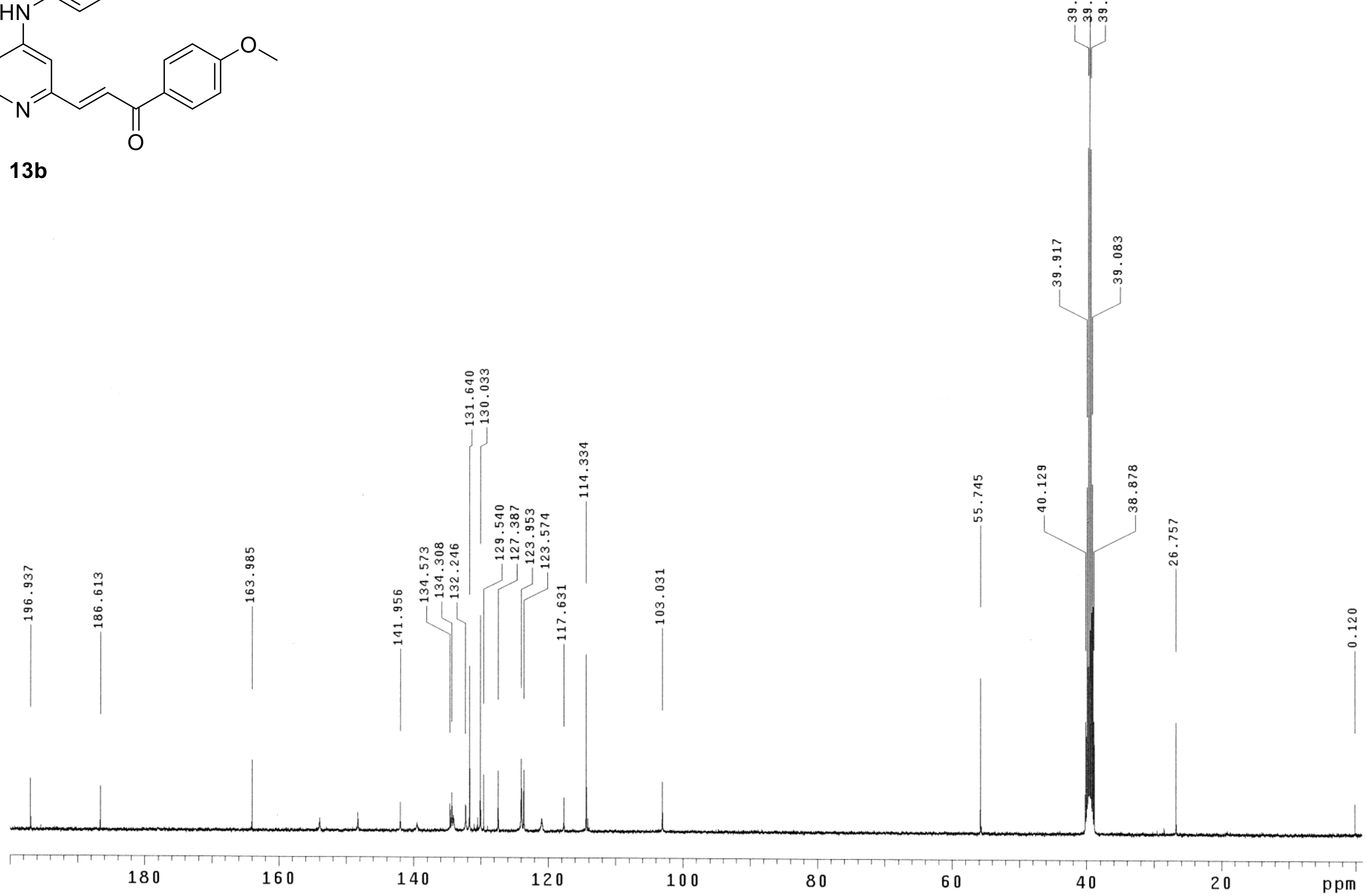

196.937
186.613
163.985
141.956
134.573
134.308
132.246
131.640
130.033
129.540
127.387
123.953
123.574
117.631
114.334
103.031
55.745
40.129
39.917
39.712
39.500
39.295
39.083
38.878
26.757
0.120
180
160
140
120
100
80
60
40
20
ppm

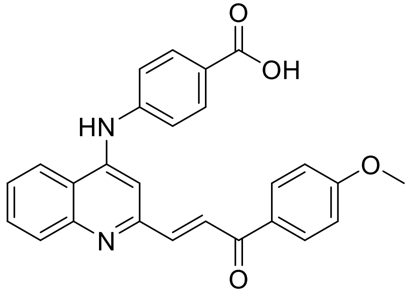

13c

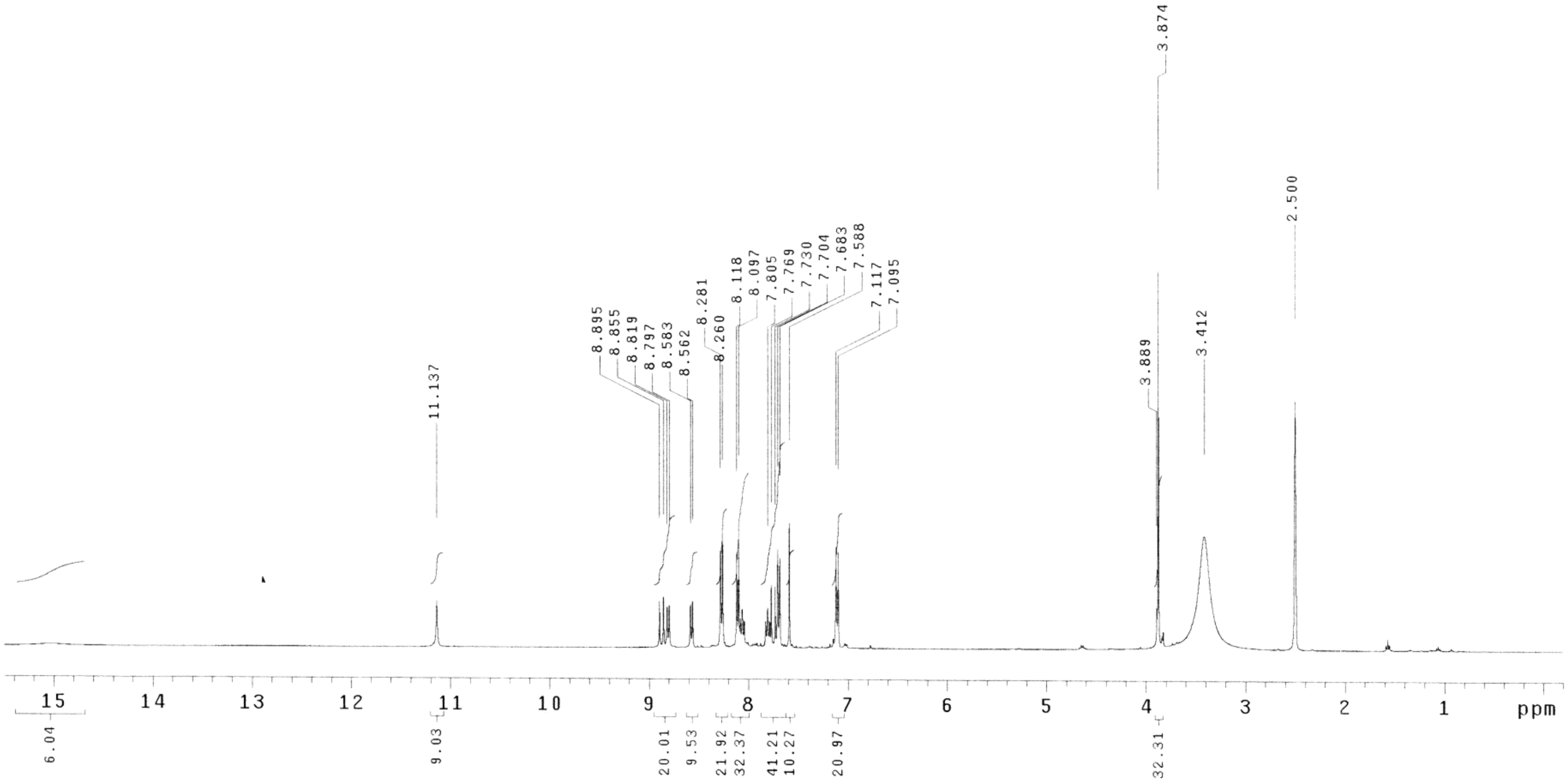

11.137
8.895
8.855
8.819
8.797
8.583
8.562
8.281
8.260
8.118
8.097
7.805
7.769
7.730
7.704
7.683
7.588
7.117
7.095
3.874
3.889
3.412
2.500
ppm
6.04
9.03
20.01
9.53
21.92
32.37
41.21
10.27
20.97
32.31

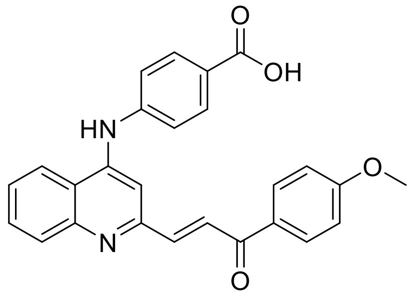
HN
OH
O
N
O
O

13c

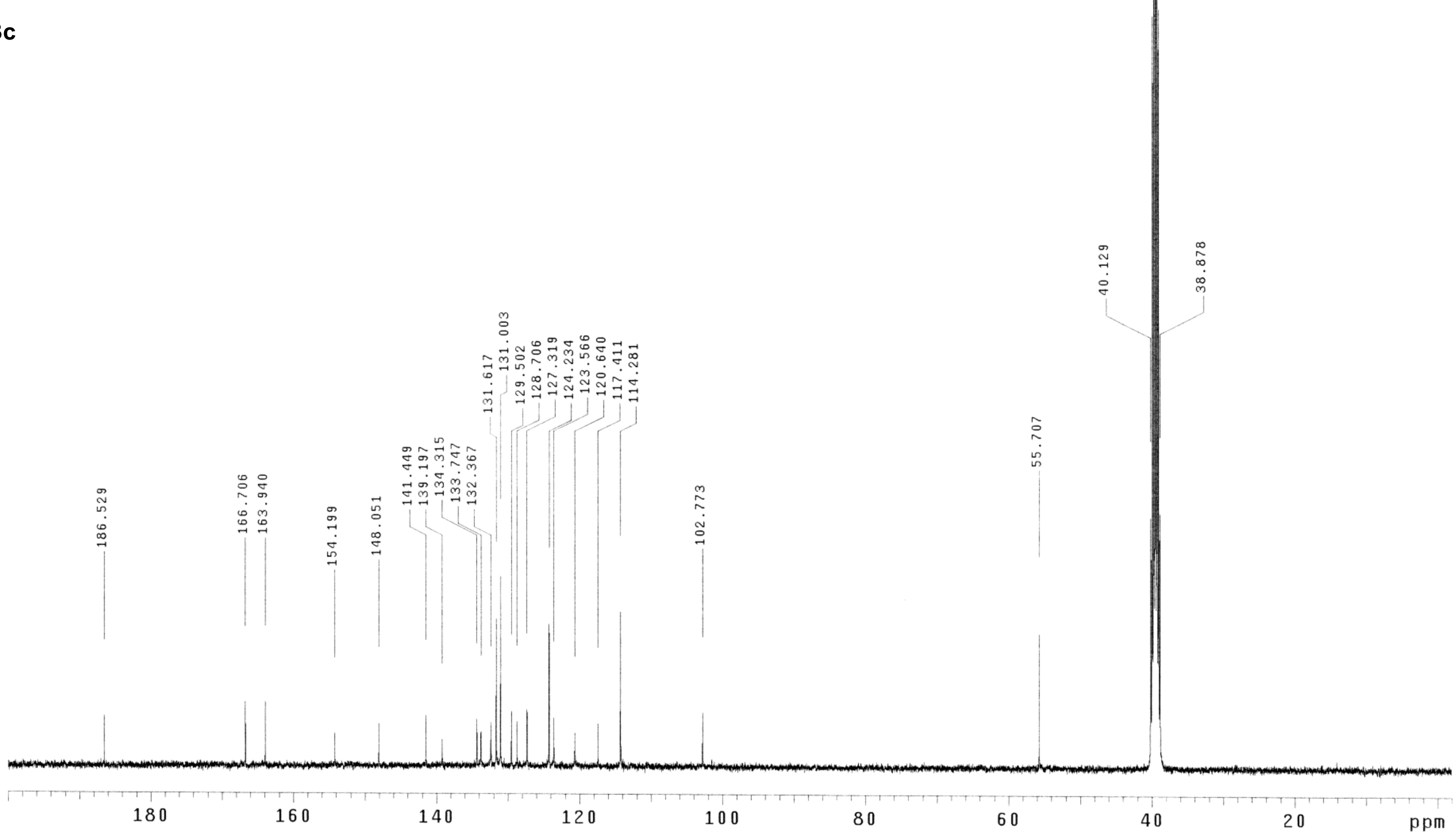
186.529
166.706
163.940
154.199
148.051
141.449
139.197
134.315
133.747
132.367
131.617
131.003
129.502
128.706
127.319
124.234
123.566
120.640
117.411
114.281
102.773
55.707
40.129
39.917
39.712
39.500
39.295
39.083
38.878
180
160
140
120
100
80
60
40
20
ppm

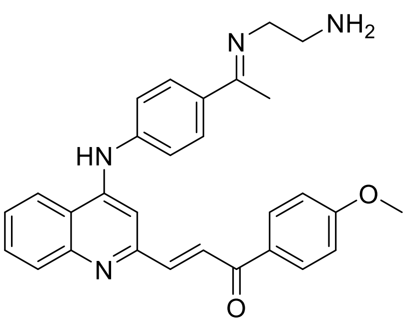

13d

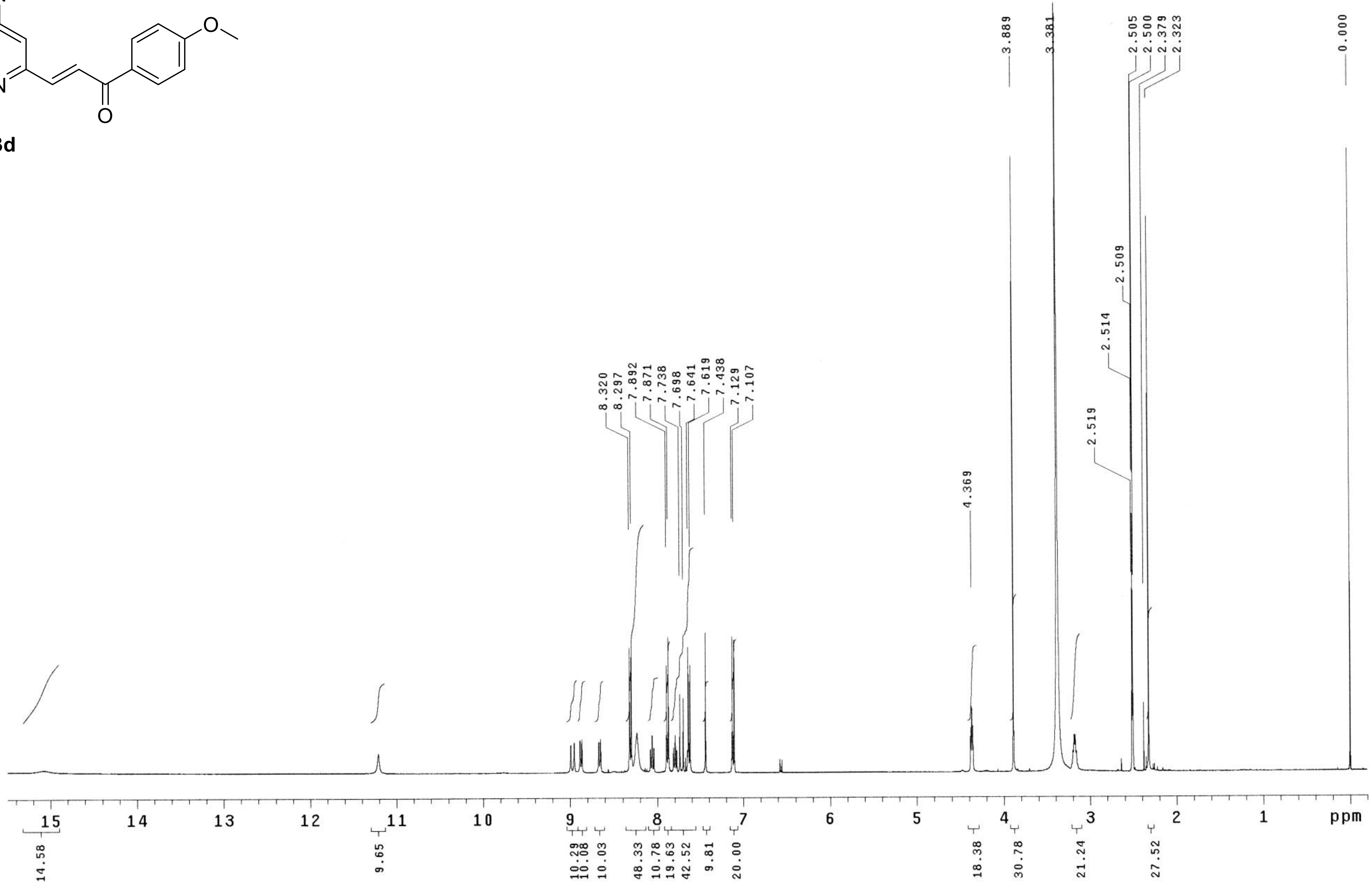

3.889
3.381
2.505
2.500
2.379
2.323
0.000
2.509
2.514
2.519
8.320
8.297
7.892
7.871
7.738
7.698
7.641
7.619
7.438
7.129
7.107
4.369
15
14
13
12
11
10
9
8
7
6
5
4
3
2
1
ppm
14.58
9.65
10.29
10.08
10.03
48.33
10.78
19.63
42.52
9.81
20.00
18.38
30.78
21.24
27.52

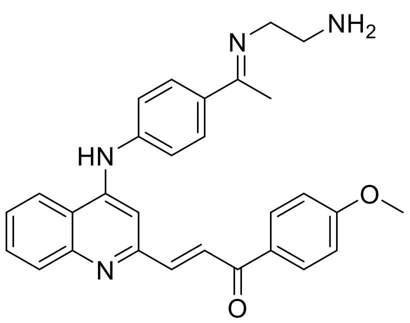

13d

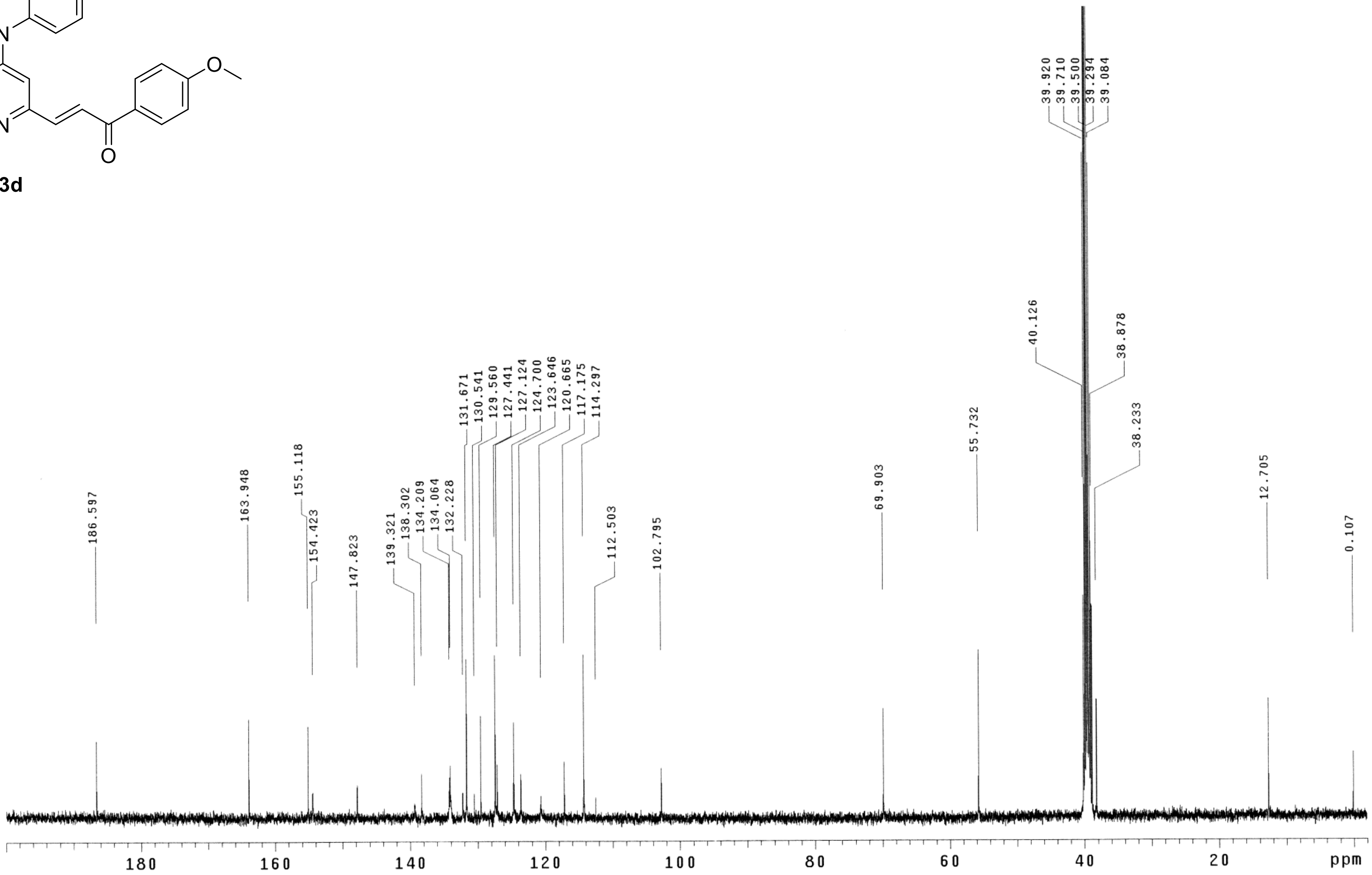
186.597
163.948
155.118
154.423
147.823
139.321
138.302
134.209
134.064
132.228
131.671
130.541
129.560
127.441
127.124
124.700
123.646
120.665
117.175
114.297
112.503
102.795
69.903
55.732
40.126
39.920
39.710
39.500
39.294
39.084
38.878
38.233
12.705
0.107
180
160
140
120
100
80
60
40
20
ppm

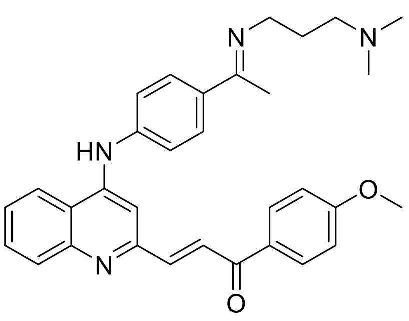

13e

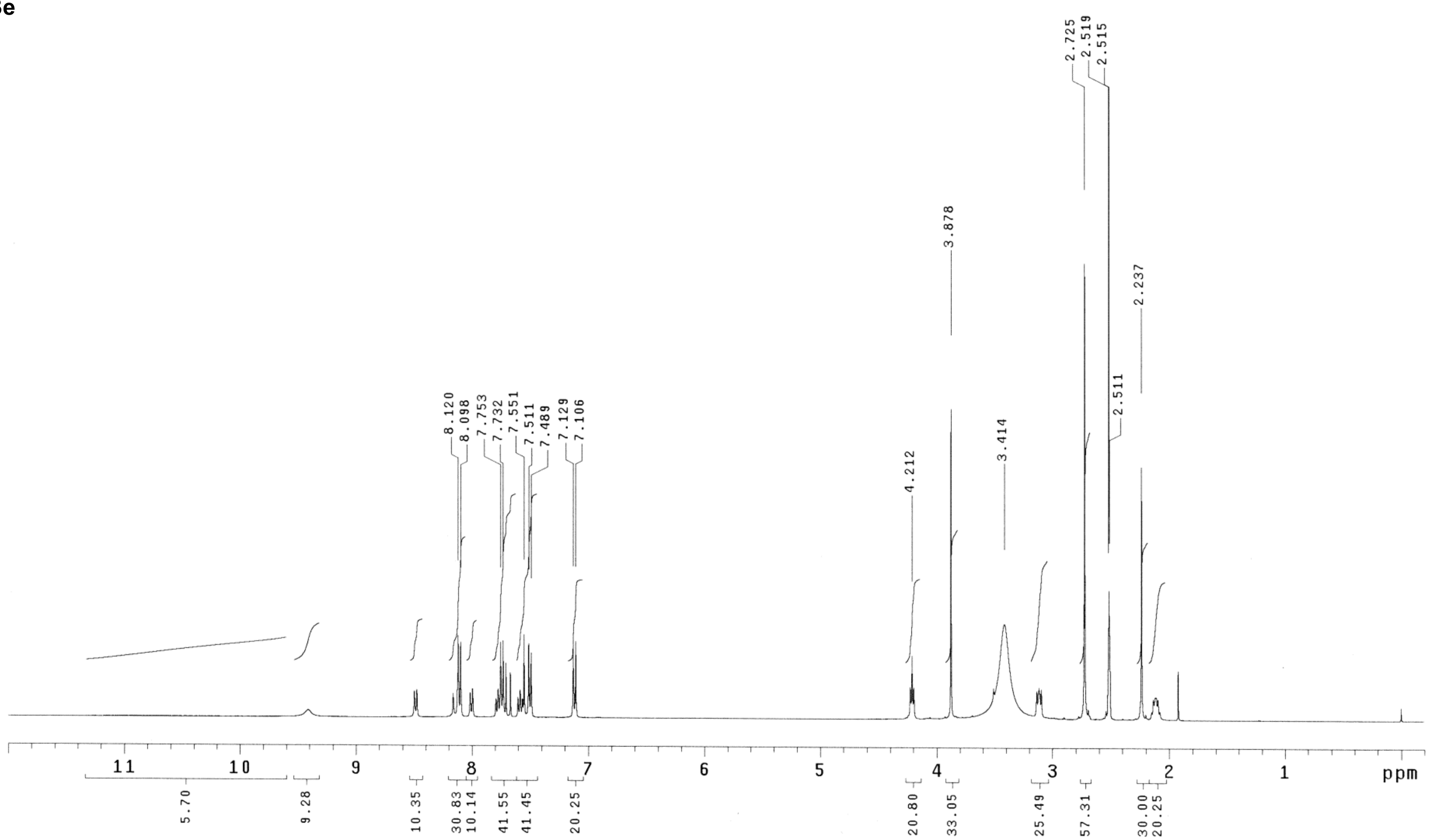
8.120
8.098
7.753
7.732
7.551
7.511
7.489
7.129
7.106
4.212
3.878
3.414
2.725
2.519
2.515
2.511
2.237
11
10
9
8
7
6
5
4
3
2
1
ppm
5.70
9.28
10.35
30.83
10.14
41.55
41.45
20.25
20.80
33.05
25.49
57.31
30.00
20.25

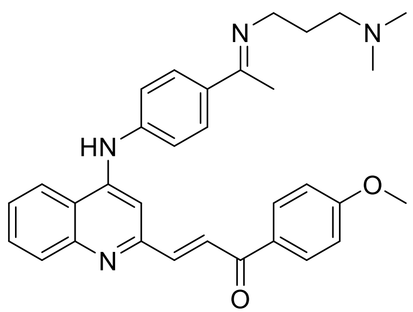
N
N
HN
N
O
O

13e

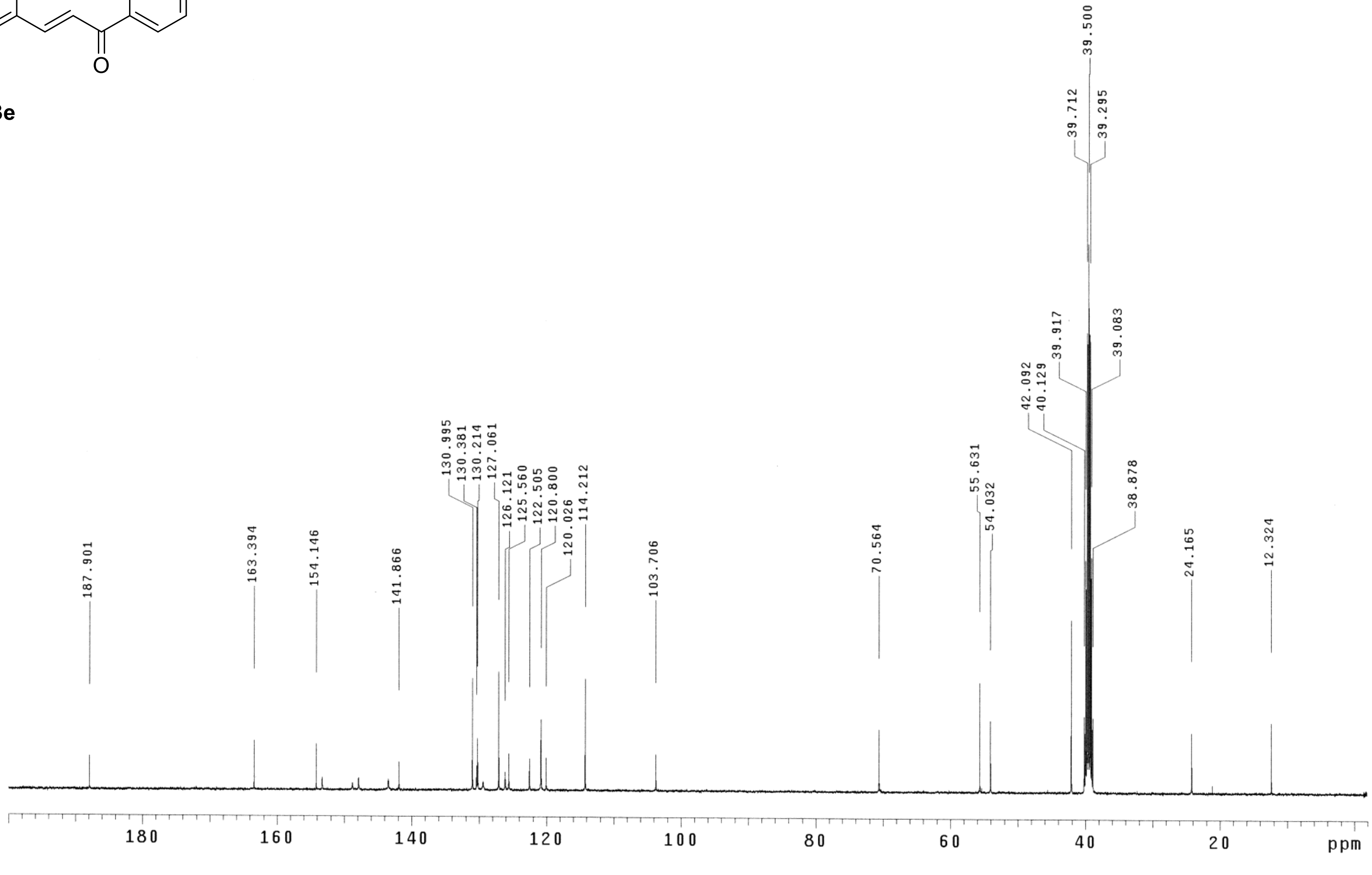
187.901
163.394
154.146
141.866
130.995
130.381
130.214
127.061
126.121
125.560
122.505
120.800
120.026
114.212
103.706
70.564
55.631
54.032
42.092
40.129
39.917
39.712
39.500
39.295
39.083
38.878
24.165
12.324
180
160
140
120
100
80
60
40
20
ppm

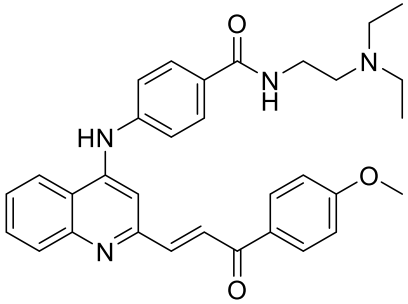
O
H
N
N
HN
O
N
O

13f

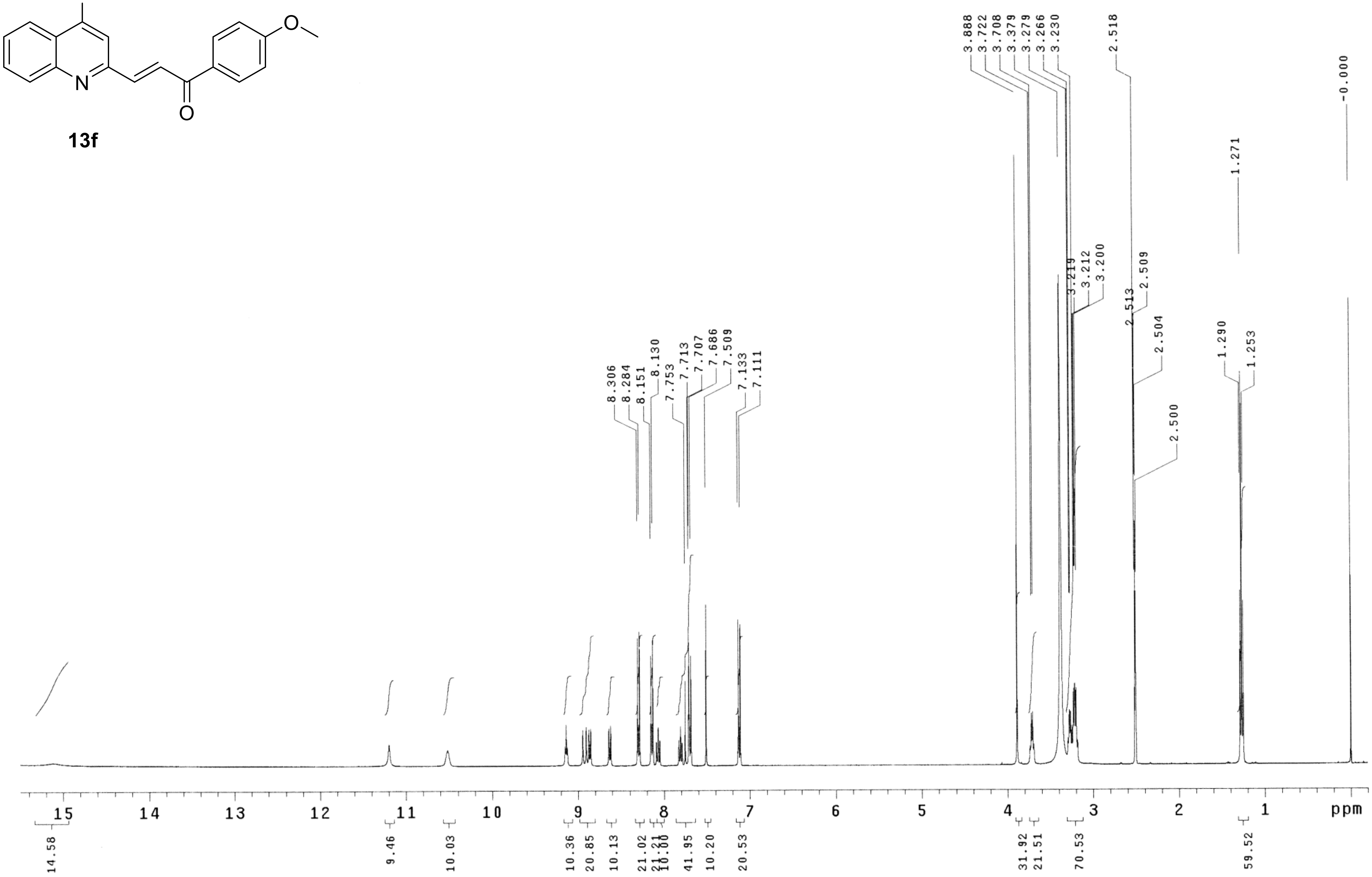
3.888
3.722
3.708
3.379
3.279
3.266
3.230
2.518
-0.000
1.271
3.219
3.212
3.200
2.509
2.513
2.504
1.290
1.253
2.500
8.306
8.284
8.151
8.130
7.753
7.713
7.707
7.686
7.509
7.133
7.111
15
14
13
12
11
10
9
8
7
6
5
4
3
2
1
ppm
14.58
9.46
10.03
10.36
20.85
10.13
21.02
21.21
10.00
41.95
10.20
20.53
31.92
21.51
70.53
59.52

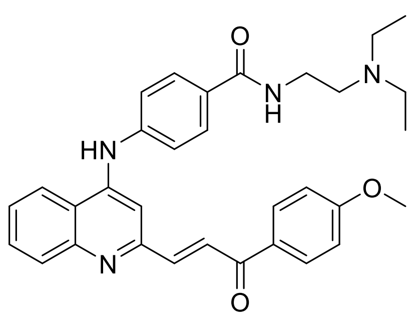

13f

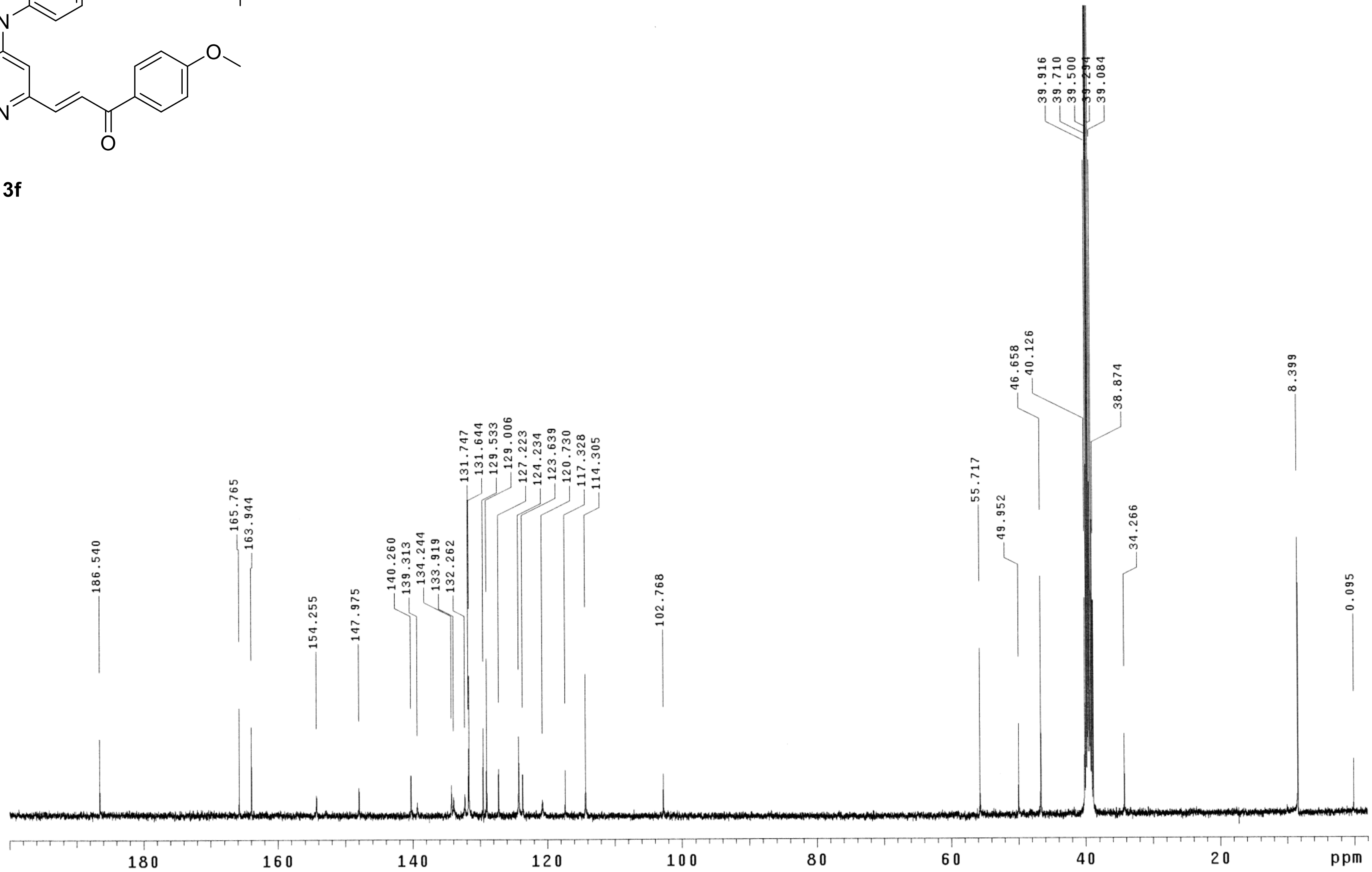

186.540
165.765
163.944
154.255
147.975
140.260
139.313
134.244
133.919
132.262
131.747
131.644
129.533
129.006
127.223
124.234
123.639
120.730
117.328
114.305
102.768
55.717
49.952
46.658
40.126
39.916
39.710
39.500
39.294
39.084
38.874
34.266
8.399
0.095
180
160
140
120
100
80
60
40
20
ppm

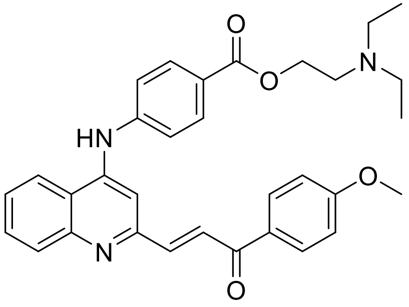

13g

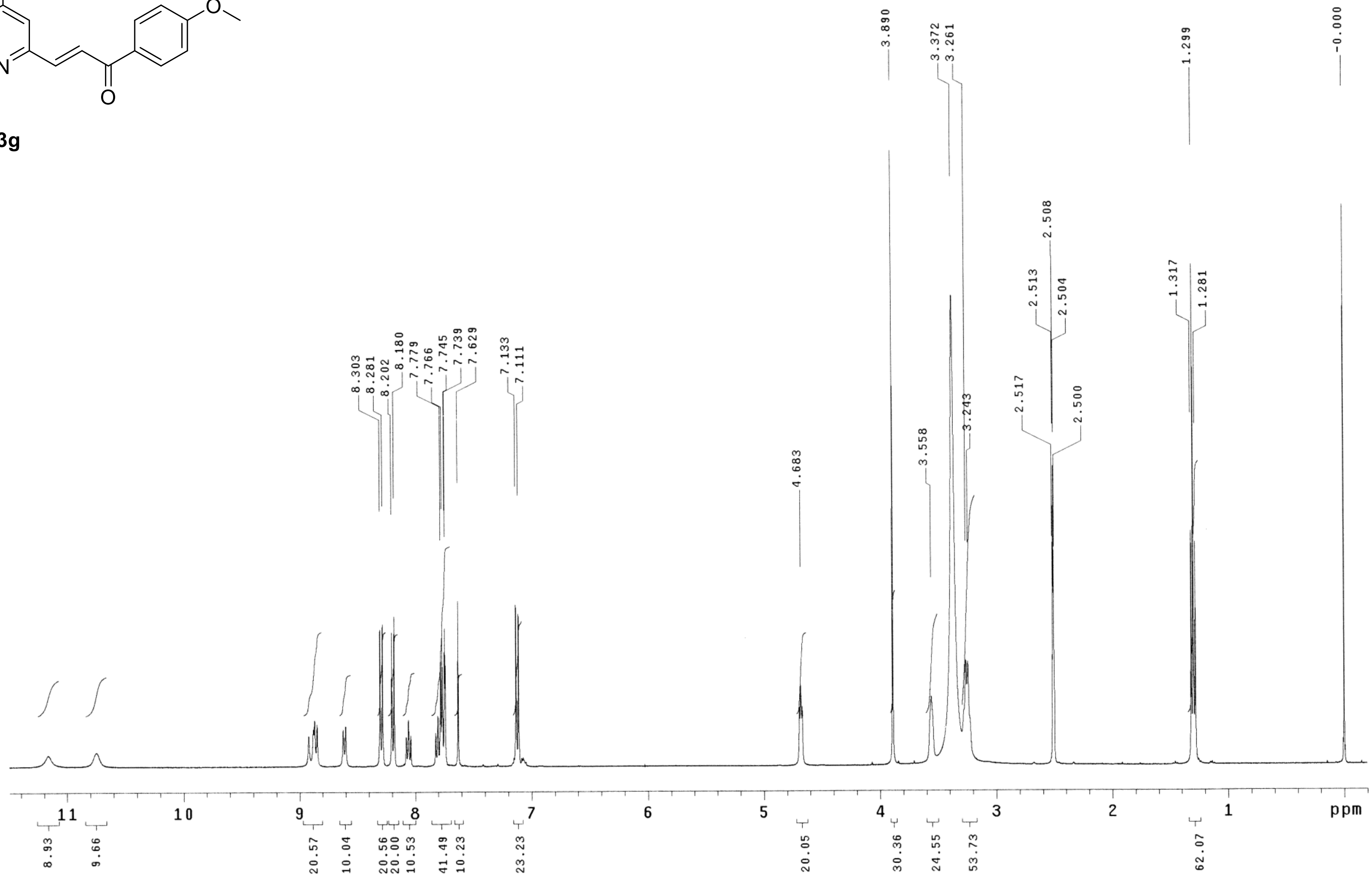

3.890
3.372
3.261
1.299
-0.000
2.508
2.513
2.504
1.317
1.281
8.303
8.281
8.202
8.180
7.779
7.766
7.745
7.739
7.629
7.133
7.111
2.517
2.500
3.243
3.558
4.683
11
10
9
8
7
6
5
4
3
2
1
ppm
8.93
9.66
20.57
10.04
20.56
20.00
10.53
41.49
10.23
23.23
20.05
30.36
24.55
53.73
62.07

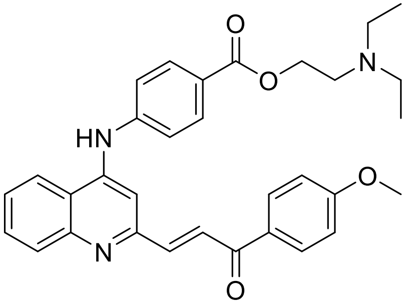

13g

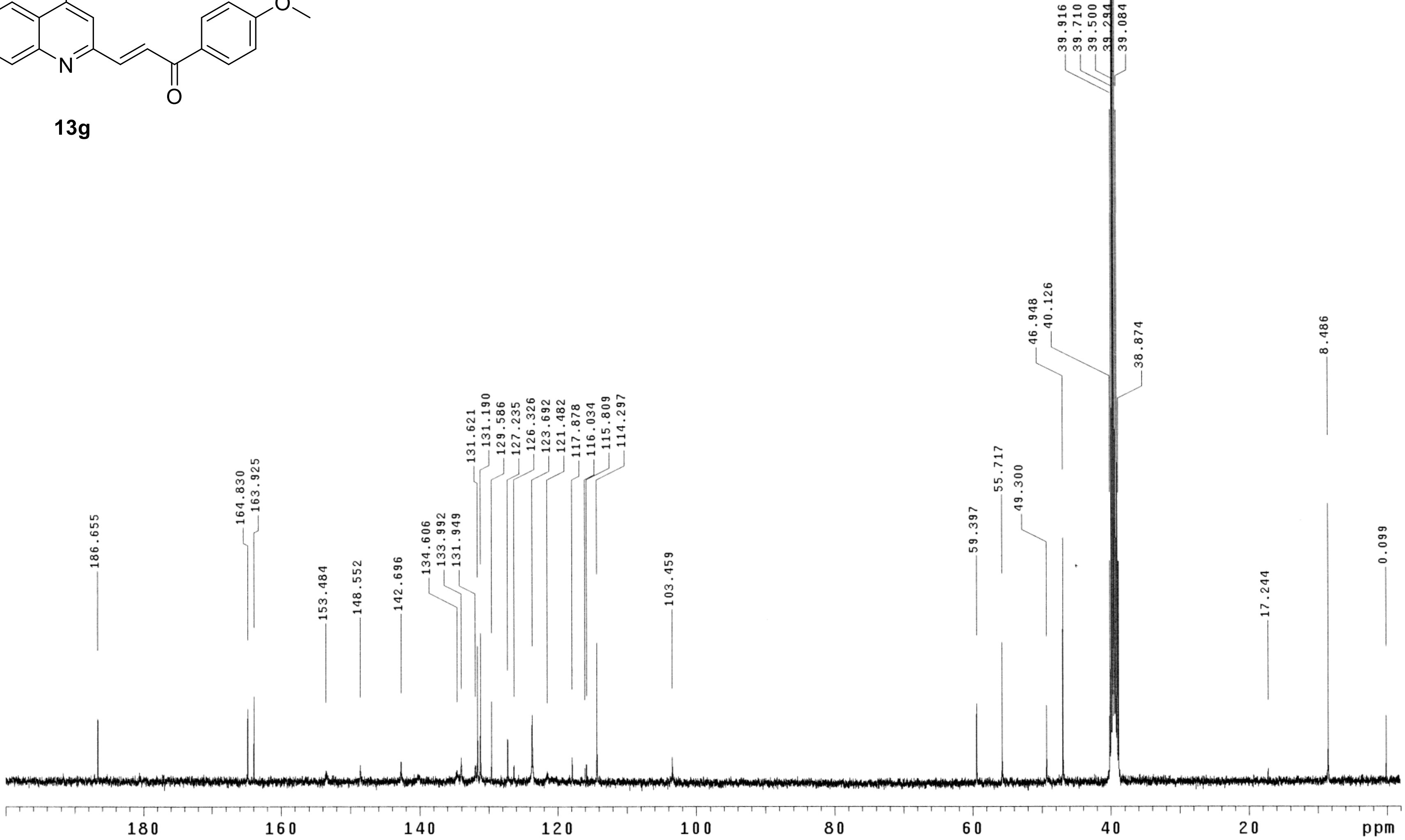
186.655
164.830
163.925
153.484
148.552
142.696
134.606
133.992
131.949
131.621
131.190
129.586
127.235
126.326
123.692
121.482
117.878
116.034
115.809
114.297
103.459
59.397
55.717
49.300
46.948
40.126
39.916
39.710
39.500
39.294
39.084
38.874
17.244
8.486
0.099
180
160
140
120
100
80
60
40
20
ppm

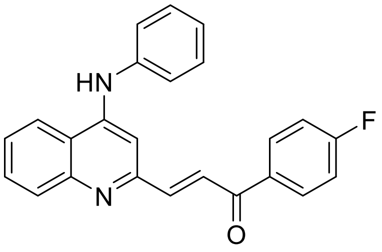
HN
F
N
O

14a

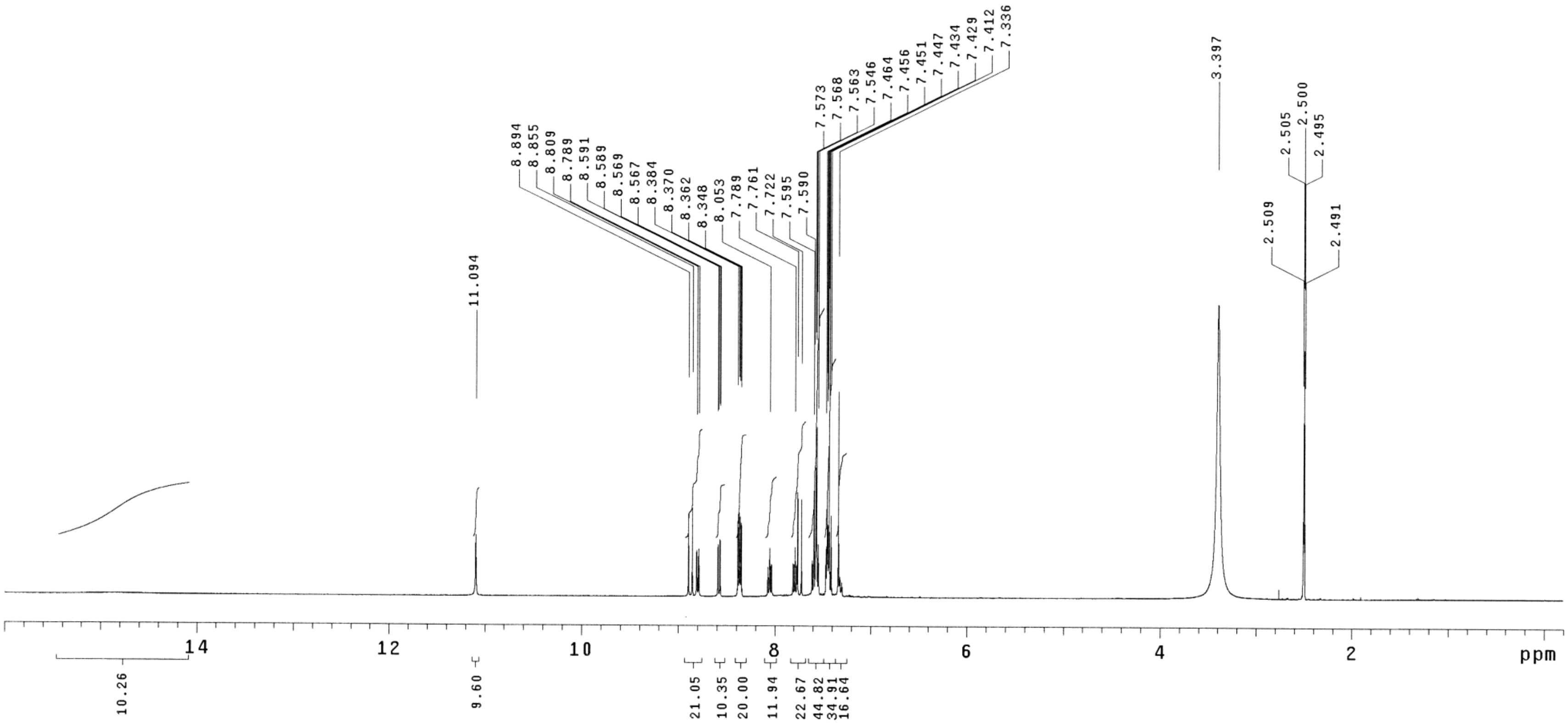
8.894
8.855
8.809
8.789
8.591
8.589
8.569
8.567
8.384
8.370
8.362
8.348
8.053
7.789
7.761
7.722
7.595
7.590
7.573
7.568
7.563
7.546
7.464
7.456
7.451
7.447
7.434
7.429
7.412
7.336
11.094
3.397
2.505
2.500
2.495
2.509
2.491
14
12
10
8
6
4
2
ppm
10.26
9.60
21.05
10.35
20.00
11.94
22.67
44.82
34.91
16.64

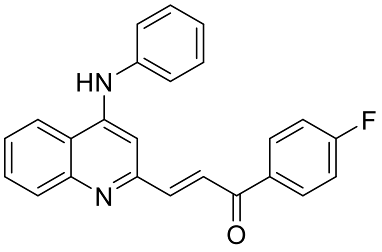
HN
F
N
O

14a

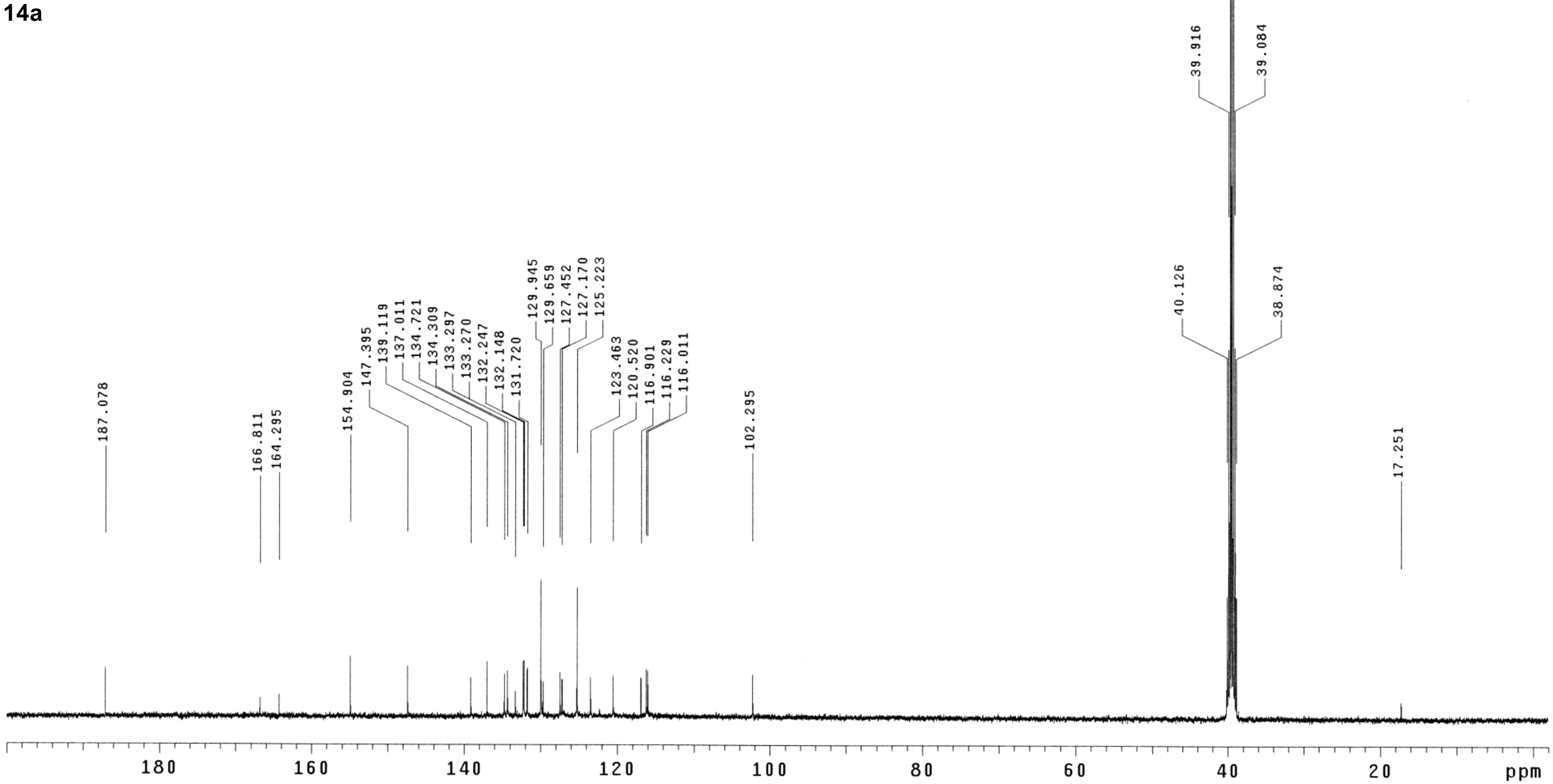
187.078
166.811
164.295
154.904
147.395
139.119
137.011
134.721
134.309
133.297
133.270
132.247
132.148
131.720
129.945
129.659
127.452
127.170
125.223
123.463
120.520
116.901
116.229
116.011
102.295
40.126
39.916
39.710
39.500
39.294
39.084
38.874
17.251
180
160
140
120
100
80
60
40
20
ppm

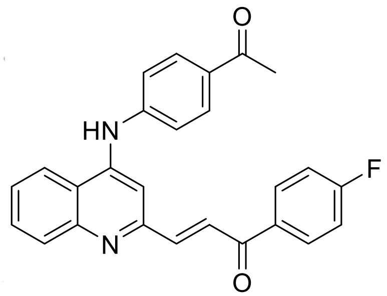

14b

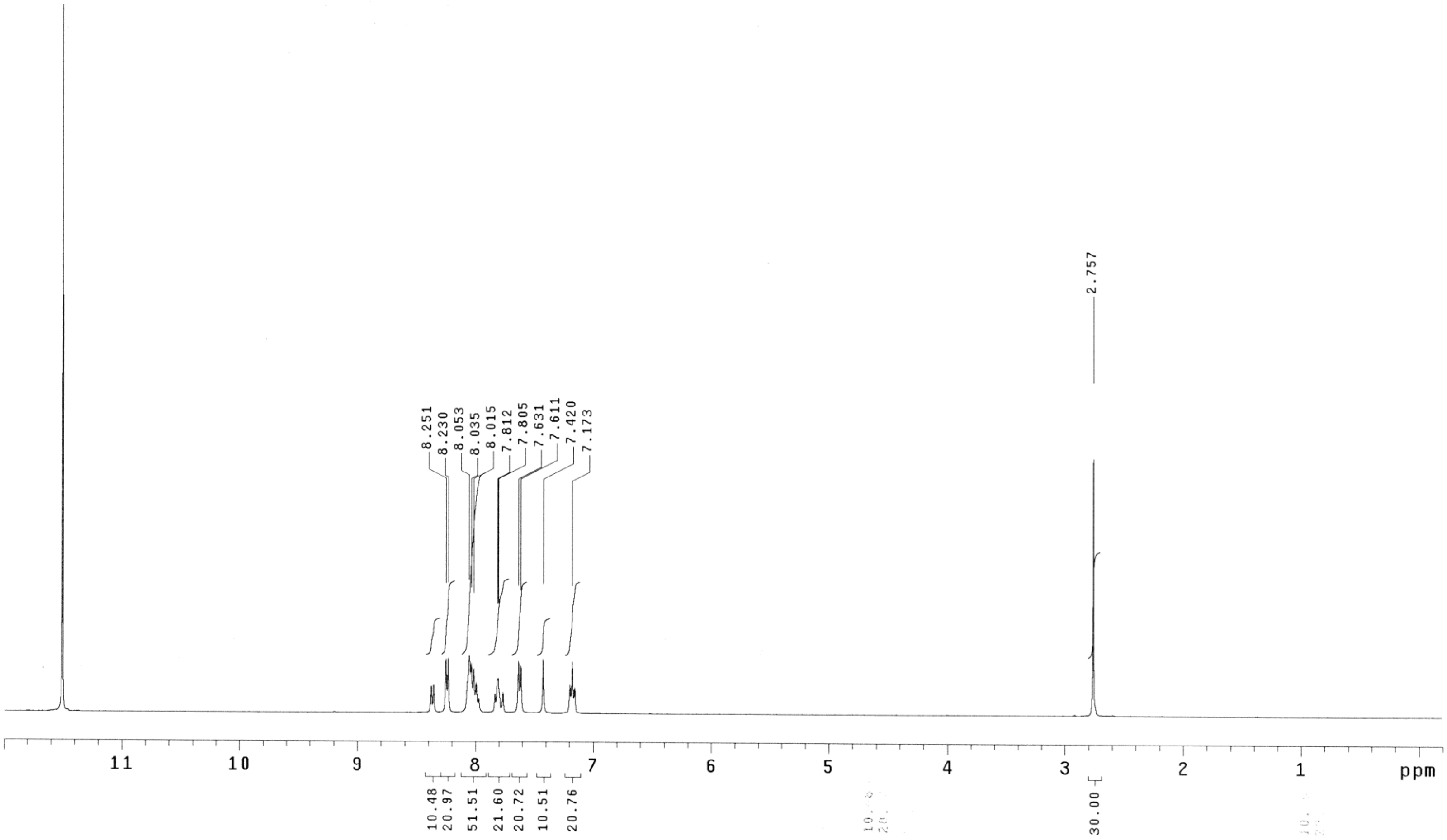

8.251
8.230
8.053
8.035
8.015
7.812
7.805
7.631
7.611
7.420
7.173
2.757
11
10
9
8
7
6
5
4
3
2
1
ppm
10.48
20.97
51.51
21.60
20.72
10.51
20.76
30.00

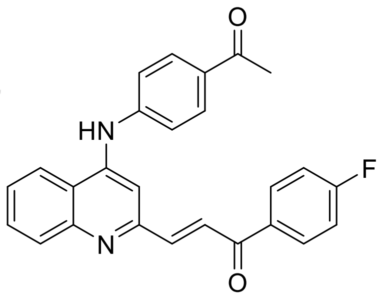
O
HN
F
N
O

14b

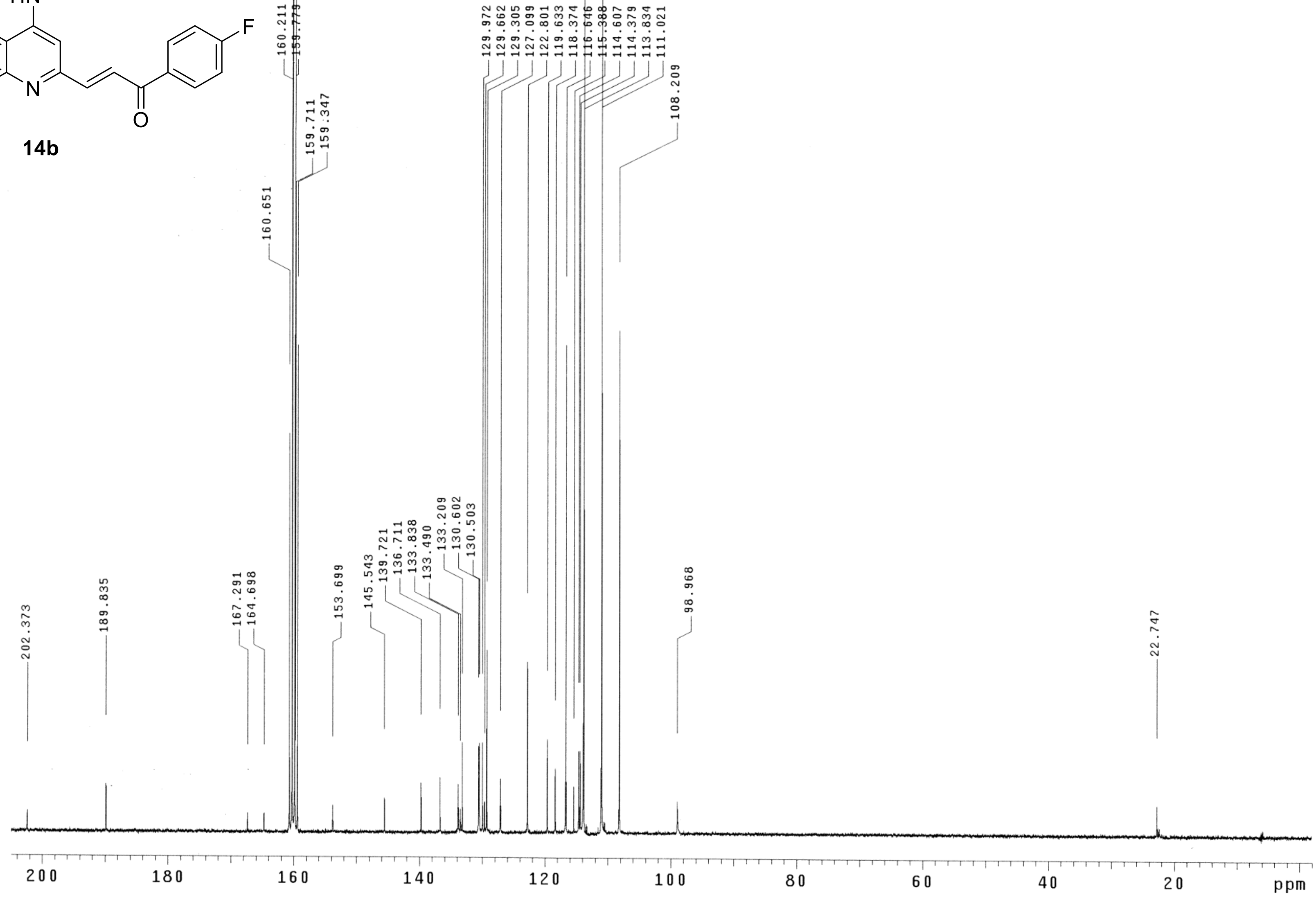
202.373
189.835
167.291
164.698
160.651
160.211
159.779
159.711
159.347
153.699
145.543
139.721
136.711
133.838
133.490
133.209
130.602
130.503
129.972
129.662
129.305
127.099
122.801
119.633
118.374
116.646
115.388
114.607
114.379
113.834
111.021
108.209
98.968
22.747
200
180
160
140
120
100
80
60
40
20
ppm

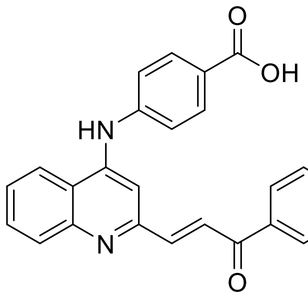

OH
HN
F
N
O
O

14c

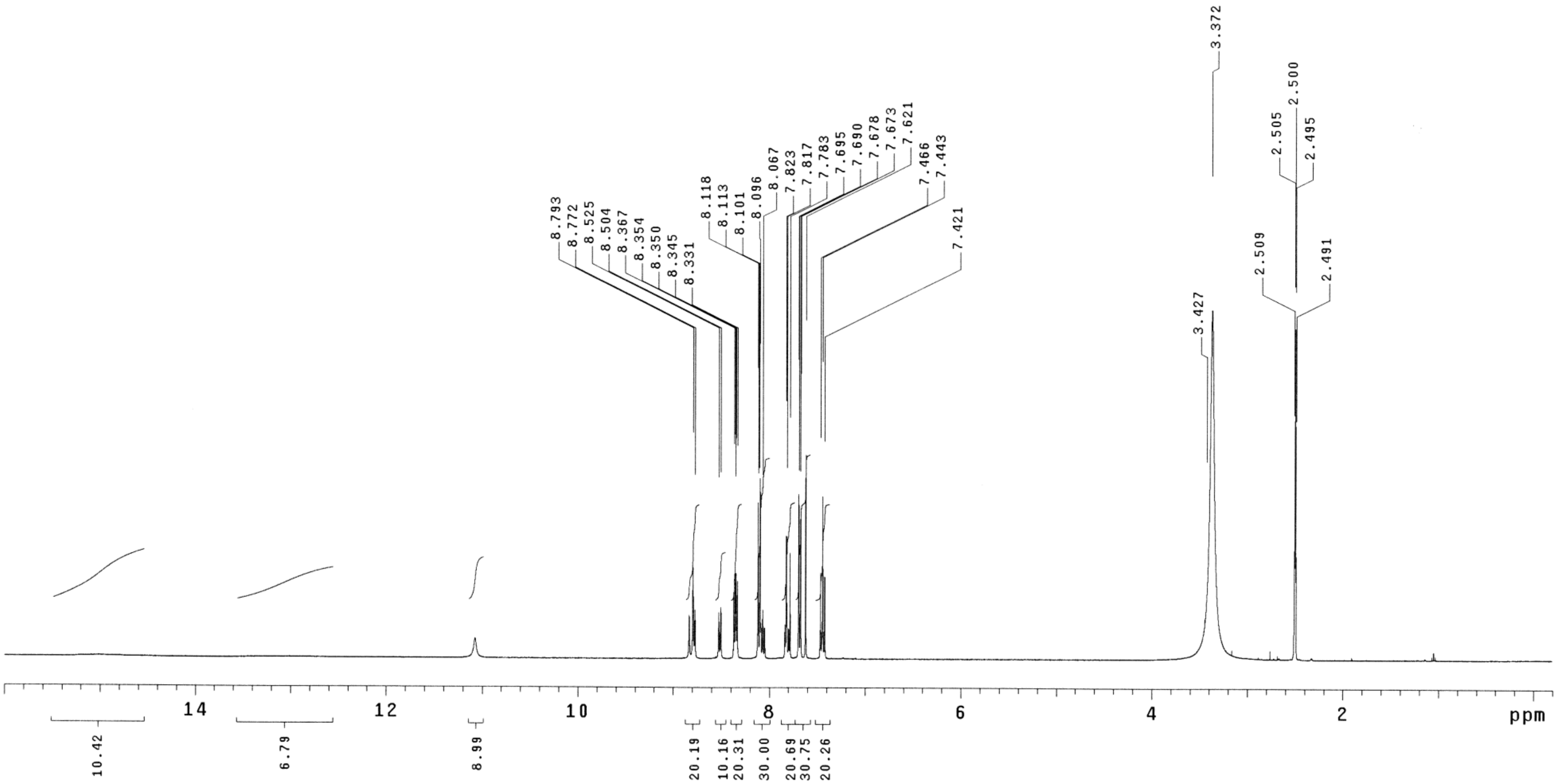

8.793
8.772
8.525
8.504
8.367
8.354
8.350
8.345
8.331
8.118
8.113
8.101
8.096
8.067
7.823
7.817
7.783
7.695
7.690
7.678
7.673
7.621
7.466
7.443
7.421
3.427
3.372
2.509
2.505
2.500
2.495
2.491
14
12
10
8
6
4
2
ppm
10.42
6.79
8.99
20.19
10.16
20.31
30.00
20.69
30.75
20.26

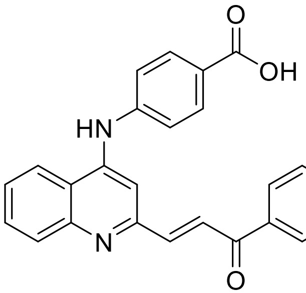
O
OH
HN
F
N
O

14c

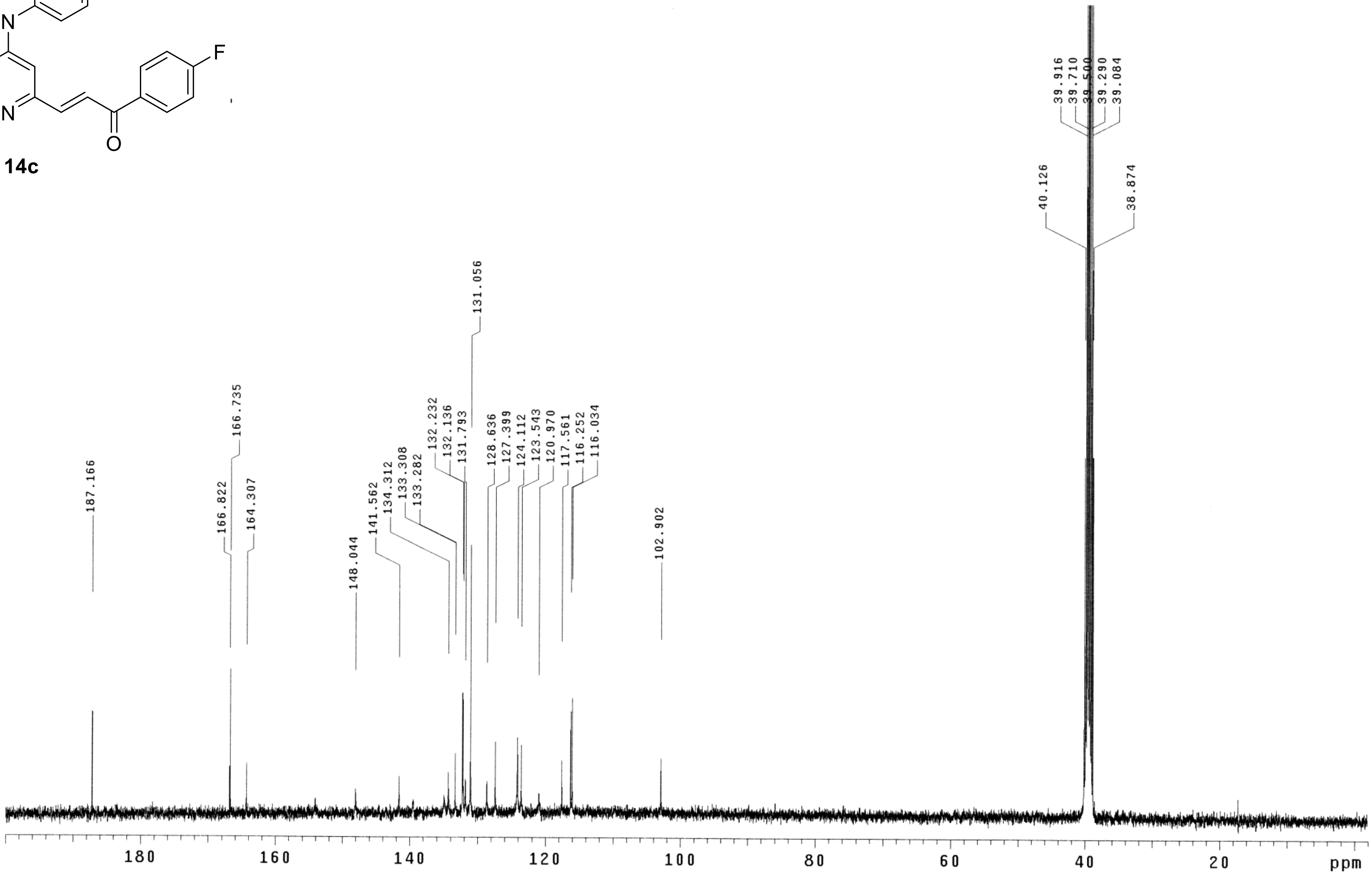
187.166
166.822
166.735
164.307
148.044
141.562
134.312
133.308
133.282
132.232
132.136
131.793
131.056
128.636
127.399
124.112
123.543
120.970
117.561
116.252
116.034
102.902
40.126
39.916
39.710
39.500
39.290
39.084
38.874
180
160
140
120
100
80
60
40
20
ppm

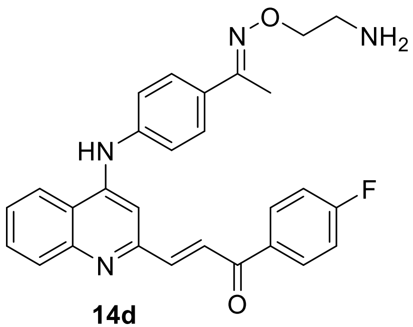
N
O
NH2
HN
F
N
O

14d

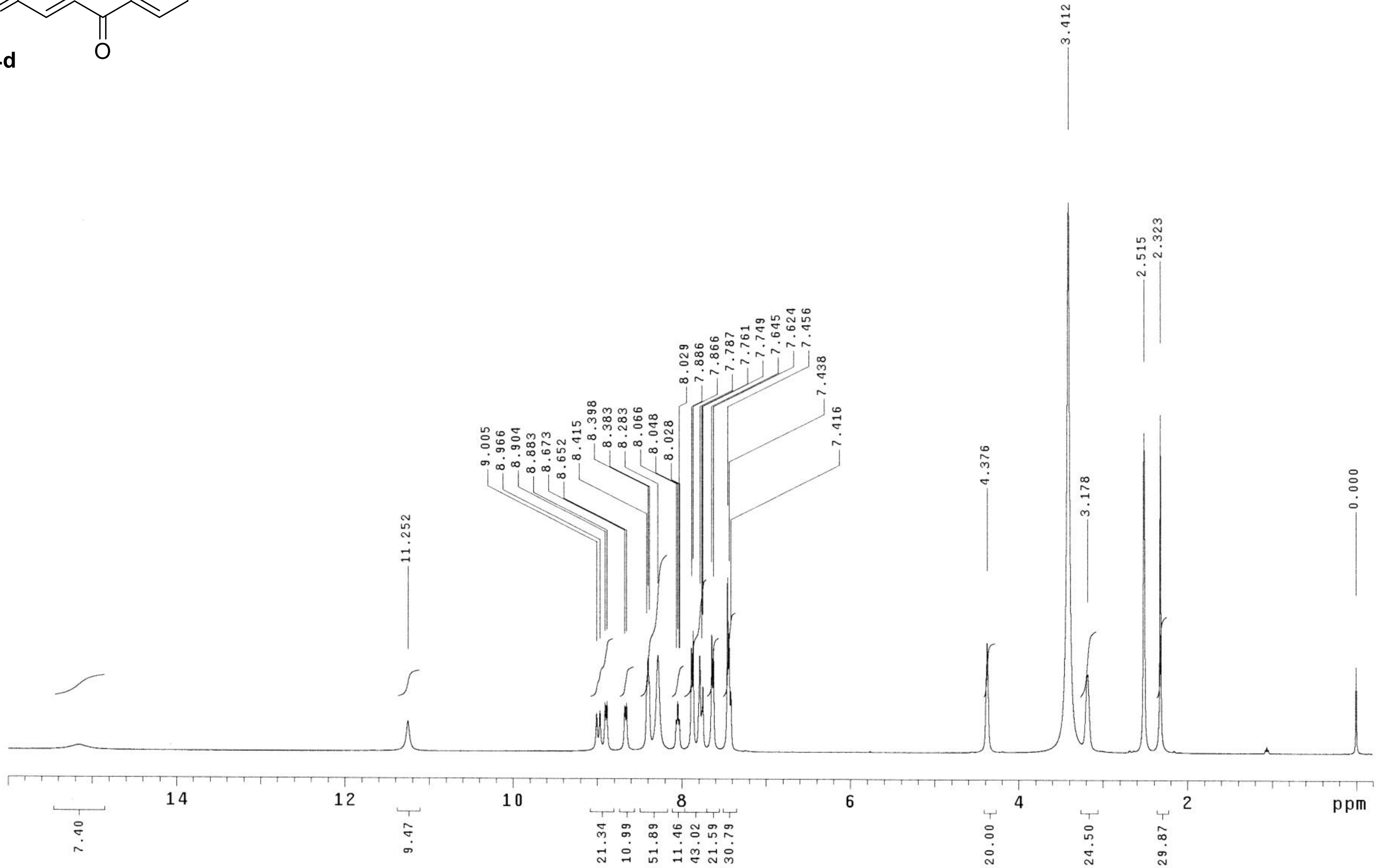
11.252
9.005
8.966
8.904
8.883
8.673
8.652
8.415
8.398
8.383
8.283
8.066
8.048
8.028
8.029
7.886
7.866
7.787
7.761
7.749
7.645
7.624
7.456
7.438
7.416
4.376
3.412
3.178
2.515
2.323
0.000
14
12
10
8
6
4
2
ppm
7.40
9.47
21.34
10.99
51.89
11.46
43.02
21.59
30.79
20.00
24.50
29.87

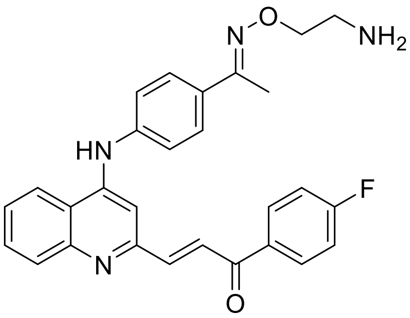
N
O
NH2
HN
F
N
O

14d

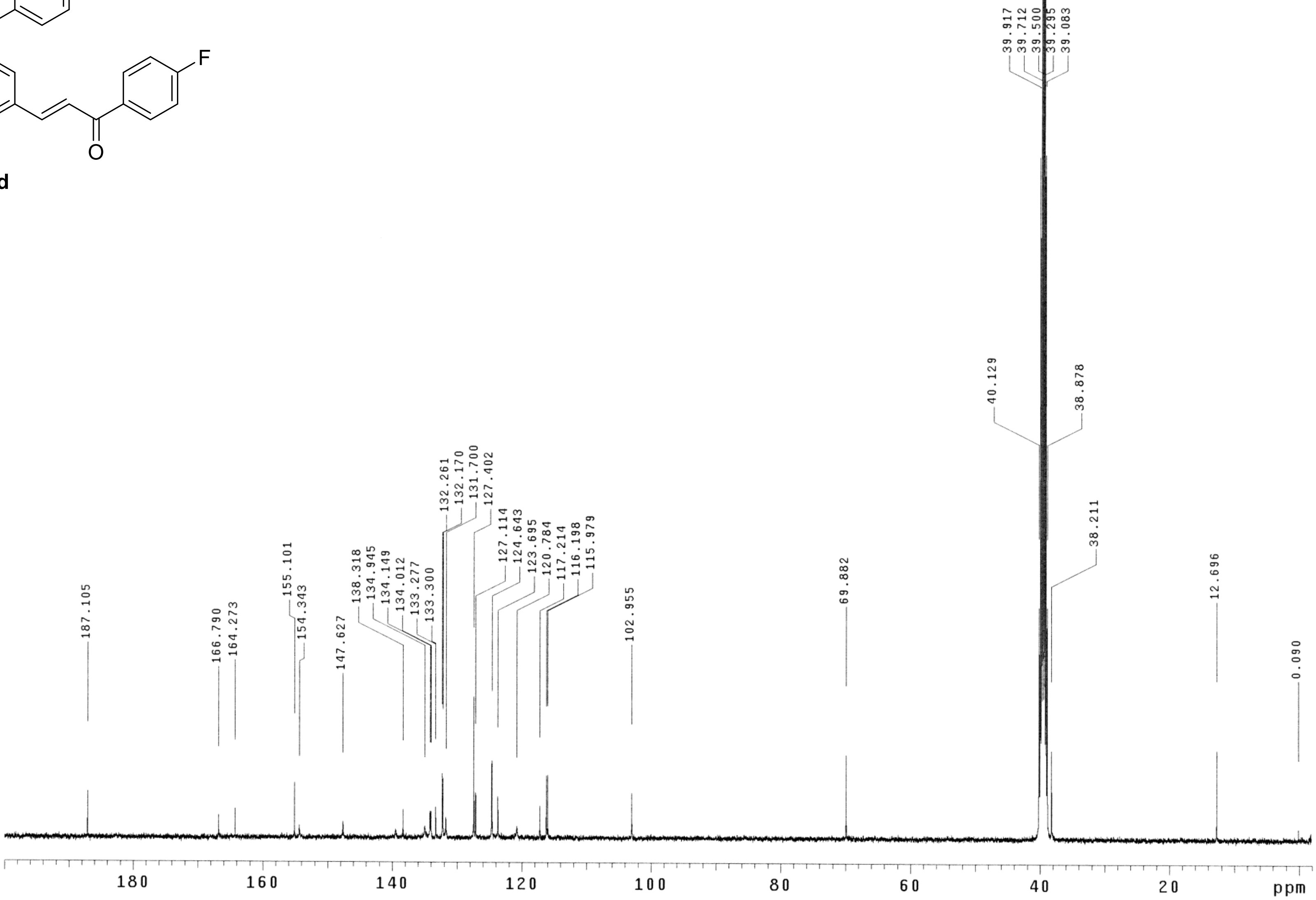
39.917
39.712
39.500
39.295
39.083
40.129
38.878
132.261
132.170
131.700
127.402
127.114
124.643
123.695
120.784
117.214
116.198
115.979
38.211
155.101
154.343
138.318
134.945
134.149
134.012
133.277
133.300
187.105
166.790
164.273
147.627
102.955
69.882
12.696
0.090
180
160
140
120
100
80
60
40
20
ppm

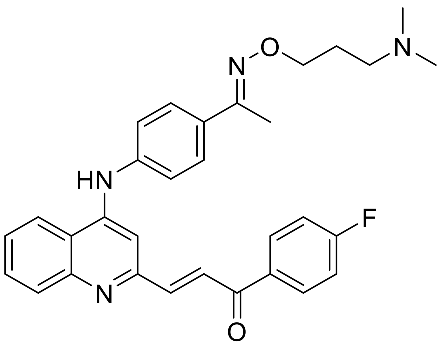

N
O
N
HN
F
N
O

14e

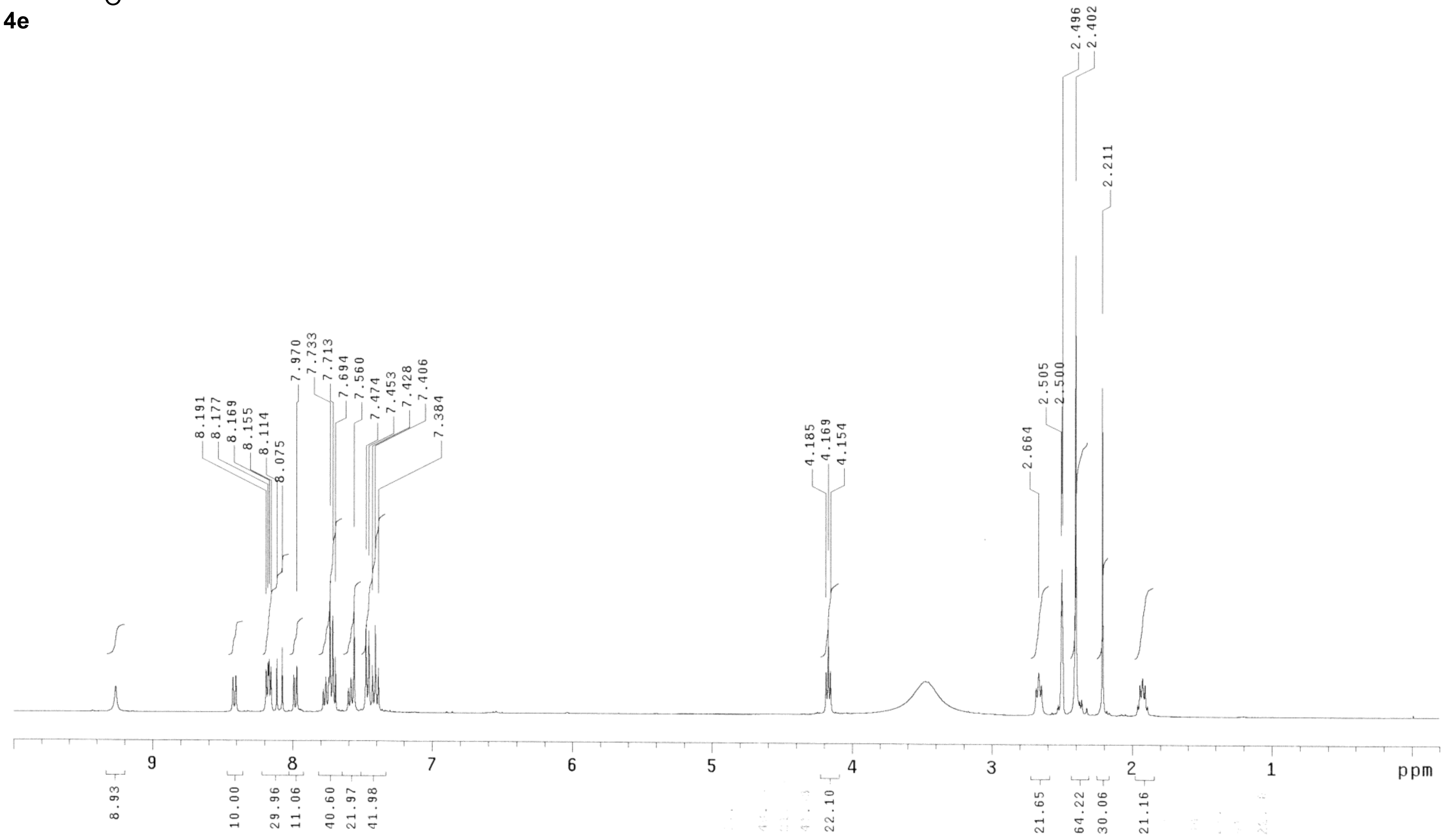

8.191
8.177
8.169
8.155
8.114
8.075
7.970
7.733
7.713
7.694
7.560
7.474
7.453
7.428
7.406
7.384
4.185
4.169
4.154
2.664
2.505
2.500
2.496
2.402
2.211
9
8
7
6
5
4
3
2
1
ppm
8.93
10.00
29.96
11.06
40.60
21.97
41.98
22.10
21.65
64.22
30.06
21.16

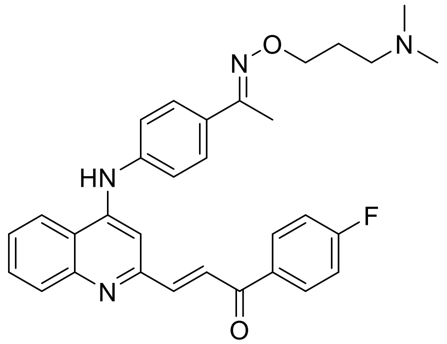

14e

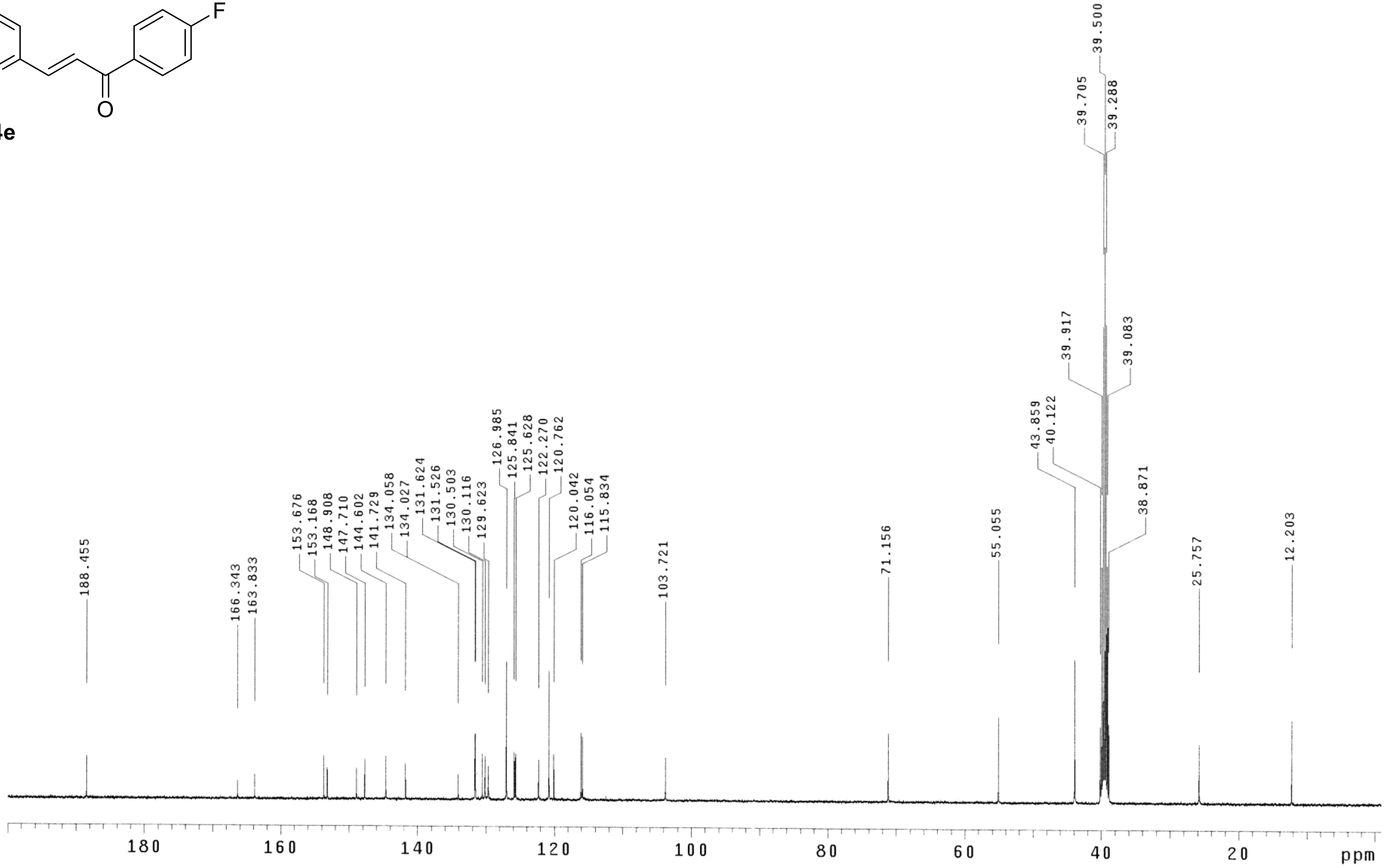
188.455
166.343
163.833
153.676
153.168
148.908
147.710
144.602
141.729
134.058
134.027
131.624
131.526
130.503
130.116
129.623
126.985
125.841
125.628
122.270
120.762
120.042
116.054
115.834
103.721
71.156
55.055
43.859
40.122
39.917
39.705
39.500
39.288
39.083
38.871
25.757
12.203
180
160
140
120
100
80
60
40
20
ppm

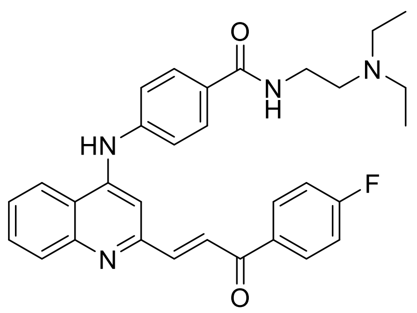

O
N
H
N
HN
F
N
O

14f

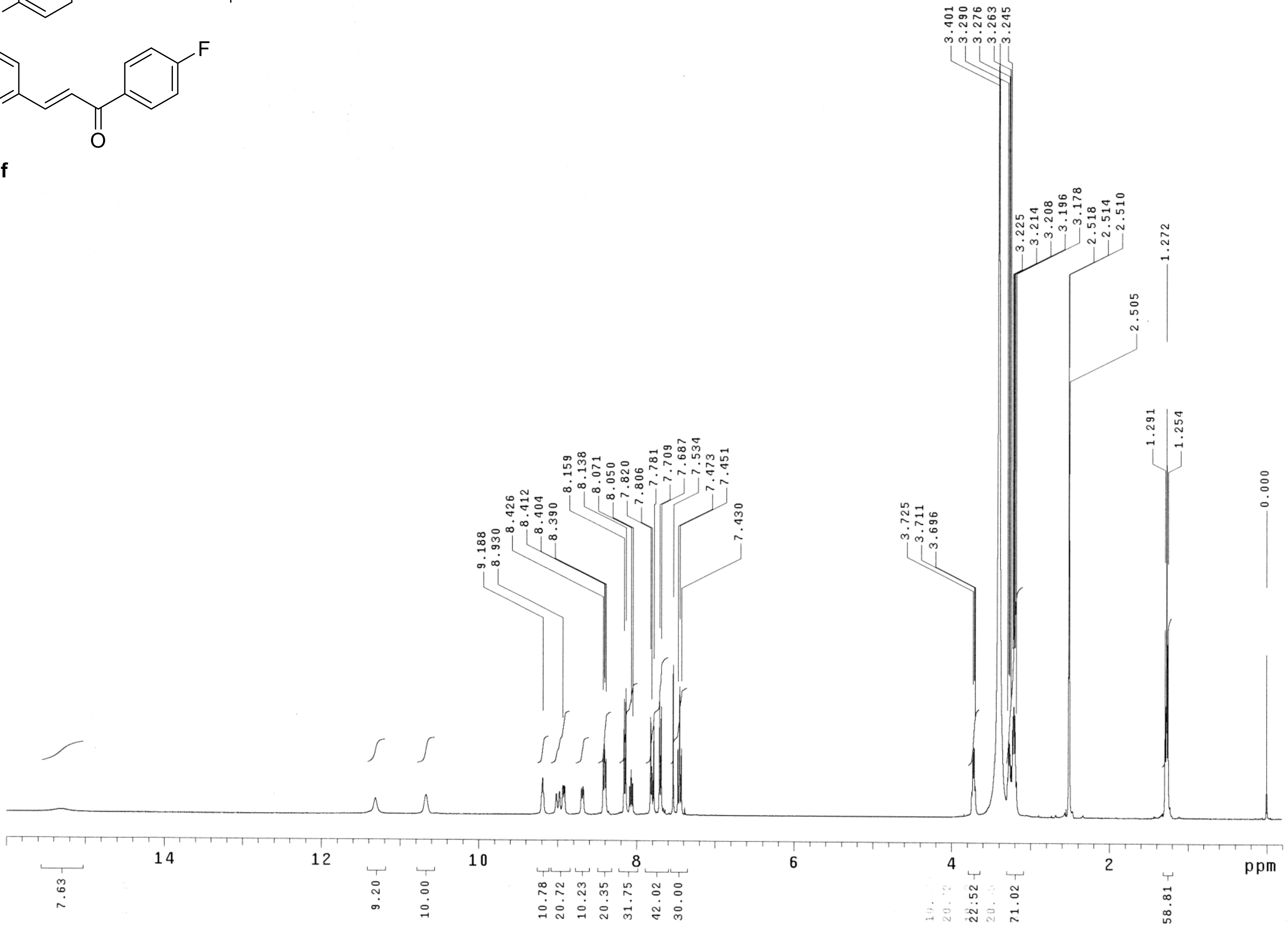

3.401
3.290
3.276
3.263
3.245
3.225
3.214
3.208
3.196
3.178
2.518
2.514
2.510
2.505
1.272
1.291
1.254
0.000
9.188
8.930
8.426
8.412
8.404
8.390
8.159
8.138
8.071
8.050
7.820
7.806
7.781
7.709
7.687
7.534
7.473
7.451
7.430
3.725
3.711
3.696
14
12
10
8
6
4
2
ppm
7.63
9.20
10.00
10.78
20.72
10.23
20.35
31.75
42.02
30.00
22.52
71.02
58.81

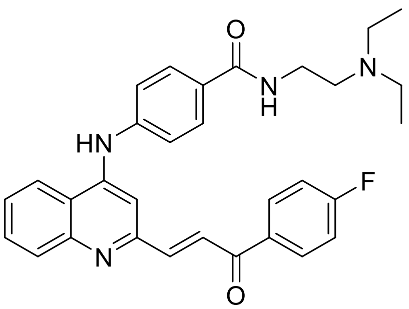

14f

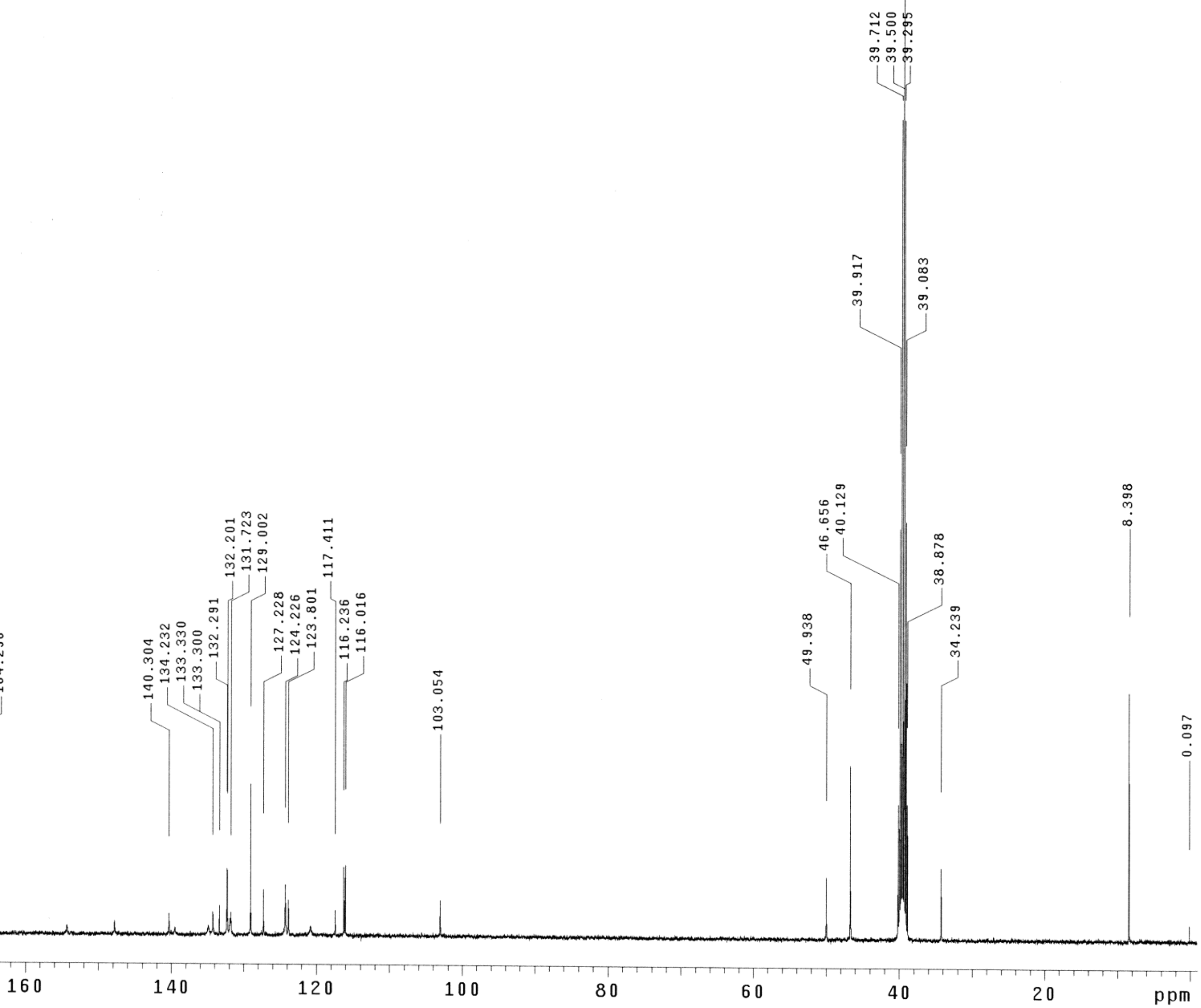

187.120
166.813
165.736
164.296
140.304
134.232
133.330
133.300
132.291
132.201
131.723
129.002
127.228
124.226
123.801
117.411
116.236
116.016
103.054
49.938
46.656
40.129
39.917
39.712
39.500
39.295
39.083
38.878
34.239
8.398
0.097
180
160
140
120
100
80
60
40
20
ppm

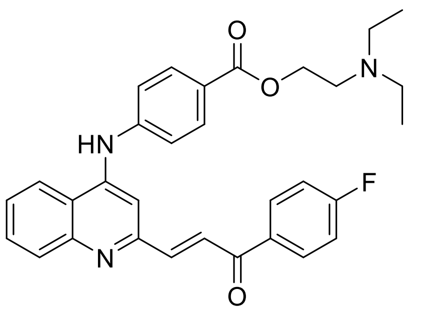

HN
N
F
O
O
O
N
O

14g

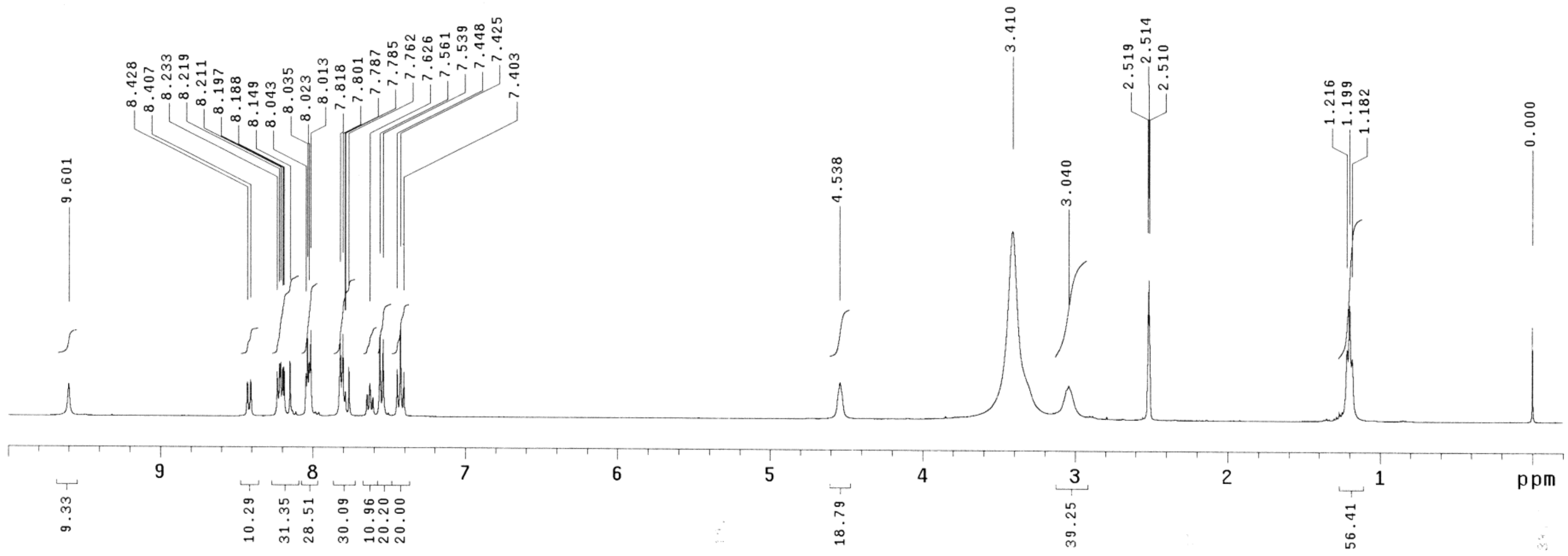

9.601
8.428
8.407
8.233
8.219
8.211
8.197
8.188
8.149
8.043
8.035
8.023
8.013
7.818
7.801
7.787
7.785
7.762
7.626
7.561
7.539
7.448
7.425
7.403
4.538
3.410
3.040
2.519
2.514
2.510
1.216
1.199
1.182
0.000
ppm
9.33
10.29
31.35
28.51
30.09
10.96
20.20
20.00
18.79
39.25
56.41

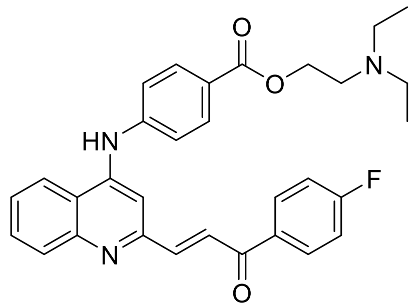

14g

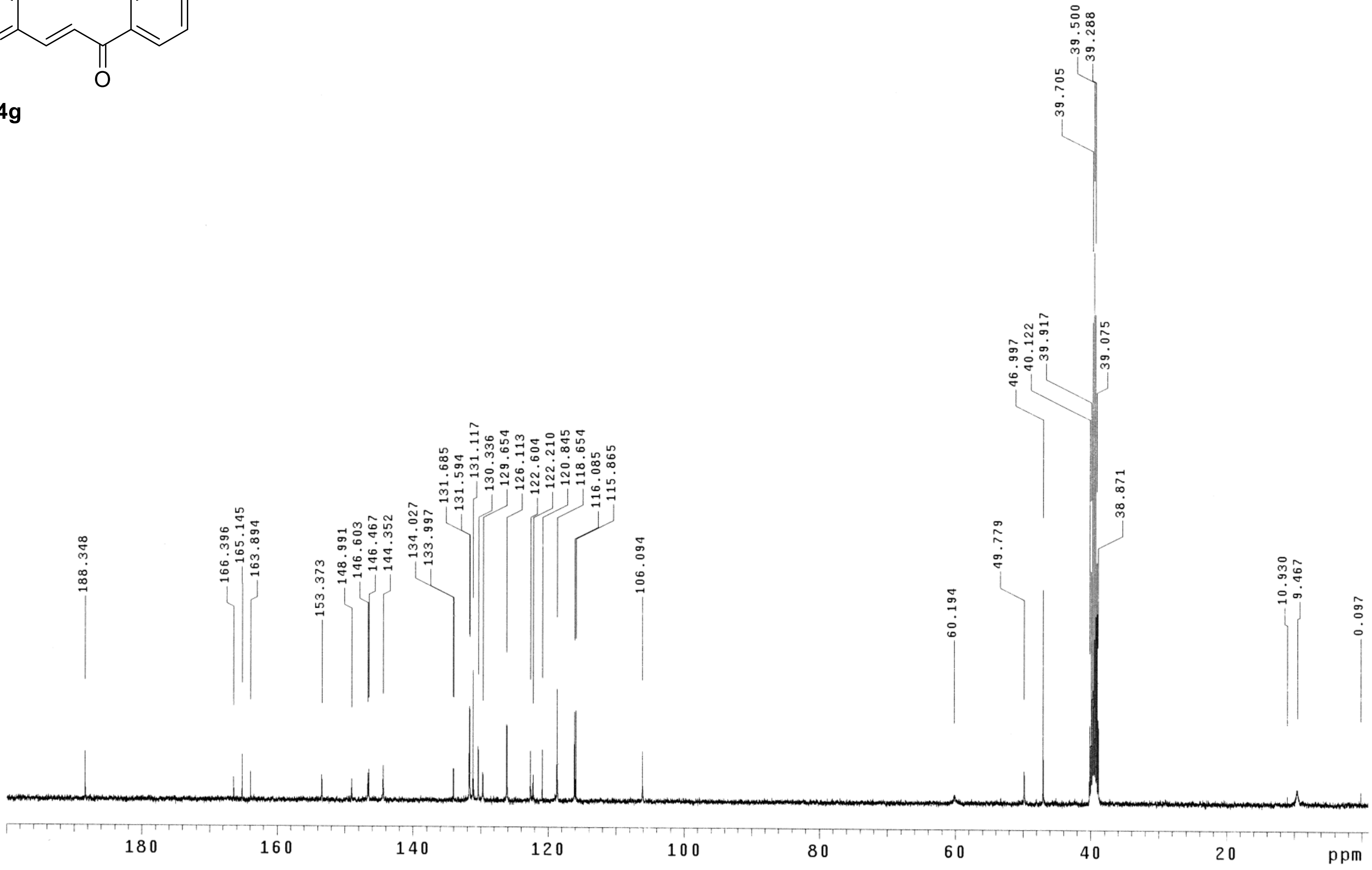
188.348
166.396
165.145
163.894
153.373
148.991
146.603
146.467
144.352
134.027
133.997
131.685
131.594
131.117
130.336
129.654
126.113
122.604
122.210
120.845
118.654
116.085
115.865
106.094
60.194
49.779
46.997
40.122
39.917
39.705
39.500
39.288
39.075
38.871
10.930
9.467
0.097
180
160
140
120
100
80
60
40
20
ppm
